# Intramolecular C-N Bond Formation via Thermal Arene C-H Bond Activation Supported by Au(III) Complexes

**DOI:** 10.3390/ma14071676

**Published:** 2021-03-29

**Authors:** Julianna Mruk, Agata J. Pacuła-Miszewska, Leszek Pazderski, Joanna Drogosz-Stachowicz, Anna E. Janecka, Jacek Ścianowski

**Affiliations:** 1 Department of Organic Chemistry, Faculty of Chemistry, Nicolaus Copernicus University, 7 Gagarin Street, 87-100 Torun, Poland; julianna@doktorant.umk.pl (J.M.); pacula@umk.pl (A.J.P.-M.); 2 Department of Analytical Chemistry and Applied Spectroscopy, Faculty of Chemistry, Nicolaus Copernicus University, 7 Gagarin Street, 87-100 Torun, Poland; leszek.pazderski@umk.pl; 3 Department of Biomolecular Chemistry, Faculty of Medicine, Medical University of Lodz, Mazowiecka 6/8, 92-215 Lodz, Poland; joanna.drogosz@stud.umed.lodz.pl (J.D.-S.); anna.janecka@umed.lodz.pl (A.E.J.)

**Keywords:** C-H activation, C-N bond formation, antioxidant activity, antiproliferative activity

## Abstract

One of the main tactics to access C-N bonds from inactivated C-H functionalities is direct transition metal-supported aminations. Due to the often harsh reaction conditions, the current goal in the field is the search for more mild and sustainable transformations. Herein, we present the first solvent-free thermally induced C-N bond formation driven by Au(III) salts. The general structure of the products was confirmed by ^1^H, ^13^C, ^15^N NMR, TGA-DTA and ATR/FT-IR analysis. Additionally, all derivatives were tested as catalysts in a three-component coupling reaction between phenylacetylene, benzaldehyde and piperidine and as anticancer agents on HL-60 and MCF-7 cell lines.

## 1. Introduction

**Scheme 1:**
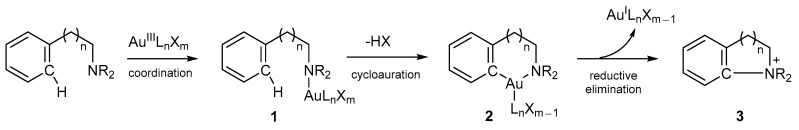
C_aryl_-N bond formation through cyclometallation.

Direct functionalization of an inactive and highly stable C-H bond, feasible in mild reaction conditions, remains a challenge in the field of organometallic reactions. Over the years, transition metals such as Pd, Pt, Rh, Ir and Ru have been widely utilized as metal centers in catalytic systems and have enabled the design of various transformation protocols [1,2]. Among various transition metals Au also started to be recognized as a promising constituent in organic synthesis. Particularly Au(III), isoelectronic to the widely used Pd(II) and Pt(II), can be easily applied in constructing analogous metal catalysts, additionally more available due to its higher abundance [3]. Gold complexes had been applied in numerous reactions enabling the formation of new C-C and C-X (X=O, N, S, halogen) bonds [4,5,6,7,8]. Most of these procedures rely on the Lewis acidity of Au, resulting in the activation of carbon–carbon π-bonds towards nucleophiles. More recently, the ability of Au(I)/Au(III) catalytic systems to perform cross-coupling transformations and arene C-H activation were also highlighted. Au(III), in the form of AuBr_3_, HAuCl_4_ and NaAuCl_4_ can be efficiently applied in nitrogen-assisted C_ary_-H activation through cyclometalation [9,10,11]. Cleavage and functionalization of the C-H bonds can be facilitated by prior nitrogen functional group coordination that directs the regiochemistry of the process. Several examples of *N*-donor ligands, like monodentate phenylpyridines [12], *N*, *C*, *N*-tridentate 1,3-(2-pirydyl) benzene [13] and *N*-confused porphirines [14], can undergo facile cycloauration to form various stable cyclometalated Au(III) species [10]. In this process, the nitrogen donor first coordinates the Au center to form the σ-complex **1**. Further intramolecular C_aryl_-H bond activation leads to the metallacycle **2**. If the nitrogen donor is also the coupling partner towards the pseudo-nucleophilic metallated carbon, it enables, through the reductive elimination of Au(I) species, a straightforward transformation of a sp^2^C-H bond to a sp^2^C-N bond (structure 3, Scheme 1).

**Scheme 2:**
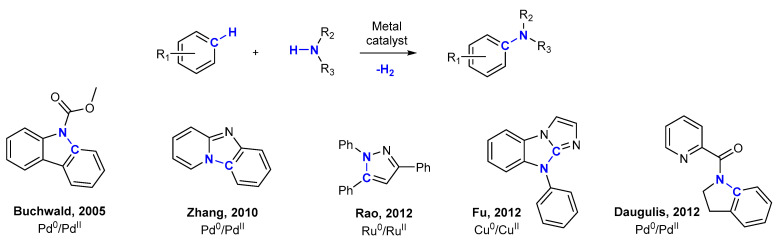
Examples of heterocycles obtained by sp^2^C-sp^2^N bond construction through CDC amination.

An ideal direct sp^2^C-N bond formation is one that can be performed through a coupling of C-H with N-H with no pre-activation of either reaction partner, defined as a cross dehydrogenative coupling (CDC) amination [15]. Until now, several research groups constructed various *N*-heterocycles by intramolecular CDC amination using Cu, Pd or Ru catalysts (Scheme 2) [16,17,18,19,20].

**Scheme 3:**
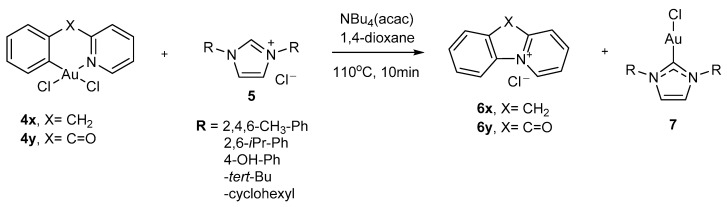
Example of sp^2^C-sp^2^N bond construction through CDC amination (carbene-induced reductive elimination) in Au-catalytic systems.

In this field, the applicability of Au-catalytic systems is limited and just starting to develop. Recently, Kim and co-workers [21] presented the first direct intramolecular sp^2^C-sp^2^N bond formation through carbene-induced reductive elimination from two Au(III) dichloride organometallics containing C(2′)-deprotonated, N(1),C(2′)-chelating 2-benzylpyridine* or 2-benzoylpyridine* anionic ligands ([Au^III^(2-benzylpyridine*)Cl_2_]—**4x**; [Au^III^(2-benzoylpyridine*)Cl_2_]—**4y**). Heating the **4x** or **4y** metallacycles with various imidazolium-derived cations **5** (1,3-bis(2,4,6-trimethylphenyl)-, 1,3-bis(2,6-diisopropylphenyl)-, 1,3-bis(4-hydroxophenyl)-, 1,3-di-*tert*-butyl- and 1,3-dicyclohexyl imidazolium; all in the chloride or tetrafluoroborate salts) in 1,4-dioxane and upon the presence of NBu_4_(acac), resulted in the formation of heterocyclic cations **6x** or **6y** (with acac^-^ counterions) and the respective Au(I)-(imidazol-2-ylidene) monochloride complexes **7**, with simultaneous HCl elimination (Scheme 3). The X-ray structure of polymeric (**6y**)_n_^n+^[AgCl_2_]_n_^n−^ salt (ROFWUH) [22], as well as the ^1^H and ^13^C NMR spectra (but not the ^15^N NMR ones) of **6x** and **6y** were also reported [21].

## 2. Materials and Methods

### 2.1. General

HAuCl_4_ was prepared by dissolving gold in aqua regia, while NaAuCl_4_ by the reaction of HAuCl_4_ with NaHCO_3_, followed by solvent evaporation. Benzaldehyde was purchased from Ubichem, phenylacetylene from TCI, and all other reagents and solvents from Sigma-Aldrich. Column chromatography was performed using Merck 40-63D 60 Å silica gel.

Elemental analyses were performed on a Vario MACRO CHN analyzer (Elementar Analysensysteme GmbH, Langenselbold, Germany). Melting points were measured with a Büchi Tottoli SPM-20 heating unit (Büchi Labortechnik AG, Flawil, Switzerland) and remained uncorrected. Thermogravimetric analysis was performed on SDT 2960 thermoanalizer (TA Instruments, New Castle, DE, USA). Far-IR spectra were measured on FT-IR Vertex 70V spectrometer (Bruker, Karlsruhe, Germany). Mass spectrum was collected on a Shimadzu High Performance Liquid Chromatograph/Mass Spectrometer LCMS-8030 (Shimadzu, Kyoto, Japan). ^1^H and ^13^C NMR spectra (including ^1^H-^13^C HSQC and HMBC) were measured by a Bruker Avance III 400 MHz NMR spectrometer, while the ^1^H-^15^N HMBC ones by a Bruker Avance III 700 MHz spectrometer, at 295–300 K, in CDCl_3_ or DMSO-d_6_ (Bruker, Karlsruhe, Germany). The ^1^H and ^13^C chemical shifts were referenced to TMS (with residual ^1^H and ^13^C solvent signals as primary references—CDCl_3_: 7.24 ppm and 77.2 ppm; DMSO-d_6_: 2.50 ppm and 39.5 ppm), while those of ^15^N—to external neat nitromethane (CH_3_NO_2_). All NMR spectra were carried out using ACD/NMR Processor Academic Edition (product version 12.01), and the most important are presented in Supplementary Materials (Figures S1–S43).

Human promyelocytic leukemia cell line (HL-60) and a solid tumor-derived human breast adenocarcinoma cell line (MCF-7) were obtained from the European Collection of Cell Cultures (ECACC, Porton Down, UK). Leukemia cells were grown in RPMI 1640 plus GlutaMax I medium (Gibco/Life Technologies, Carlsbad, CA, USA). MCF-7 cells were maintained in Minimum Essential Medium Eagle (Sigma Aldrich, St. Louis, MO, USA) and supplemented with 2 mM glutamine and Men Non-essential amino acid solution (Sigma Aldrich, St. Louis, MO, USA). Both media were supplemented with 10% heat-inactivated fetal bovine serum (Biological Industries, Beit-Haemek, Israel) and antibiotics (100 U/mL penicillin and 100 μg/mL streptomycin) (Sigma-Aldrich, St. Louis, MO, USA). The nontumorigenic mammary gland/breast MCF-10A cell line was purchased from the American Type Culture Collection (ATCC). For MCF-10A cells MEGM Mammary Epithelial Bullet Kit was used. Cells were maintained at 37 °C in a 5% CO_2_ humidified atmosphere and were grown until 80% confluent.

### 2.2. Procedures

#### 2.2.1. General Procedure for the Synthesis of Ligands **8**–**15**

This general procedure can be exemplified by the synthesis of **8**. A mixture of 2-bromopyridine (5.0 mmol), benzenethiol (5.0 mmol) and K_2_CO_3_ (10.0 mmol) in dimethyl sulfoxide (DMSO; 4 mL) was stirred at 110 °C for 24 h. It was cooled to room temperature, diluted with water (10 mL) and extracted with dichloromethane (4 × 5 mL). The organic phase was washed with water (3 × 20 mL), dried over anhydrous MgSO_4_, filtered and concentrated under vacuum. The residue was purified by column chromatography on silica gel with dichloromethane/hexane 8:2 as eluent to afford the sulfide. The **9**–**15** heterocycles were obtained similarly, using various derivatives of 2-bromopyridine (6-methyl-, 5-methyl-, 4-methyl- for **9**, **10**, **11**, respectively) or benzenethiol (4-methyl-, 4-*tert*-butyl-, 4-bromo-, 4-nitro- for **12**, **13**, **14**, **15**, respectively). The yields were as follows: **8**—78%, **9**—82%, **10**—75%, **11**—88%, **12**—79%, **13**—77%, **14**—72%, **15**—70%. The ^1^H, ^13^C and ^15^N NMR spectra of **8** and **12**–**15** were already reported and assigned in our recent paper [23], while those of **9**–**11** are described in Supplementary Data (Figures S1–S12).

#### 2.2.2. General Procedure for the Synthesis of Au(III) Trichloride Complexes **8a**–**15a**

A total of 1 mmol of NaAuCl_4_ in 18 mL of water was added to 1 mmol of the corresponding ligand **8**–**15** in 30 mL of methanol and stirred at 50 °C for 24 h. The yellow/orange/red crystals were filtered on a Büchner funnel and dried on air. This method produced Au(III) trichloride complexes **8a**–**15a** with the following yields: **8a**—78%, **9a**—86%, **10a**—88%, **11a**—86%, **12a**—69%, **13a**—79%, **14a**—77%, **15a**—72%. The ^1^H, ^13^C and ^15^N NMR spectra of **8a** and **12a**–**15a** were already reported and assigned in our recent paper [23], while those of **9a**–**11a** are given in Supplementary Data (Figures S13–S18).

#### 2.2.3. General Procedure for the Synthesis of Tetrachloroaurate(III) Salts **8b**–**15b**

The 0.20 mmol of the corresponding Au(III) trichloride complex **8a**–**15a** was placed in one-neck RBF under vacuum (0.1 mm Hg) and heated at certain temperature (**8a**—180 °C, **9a**—150 °C, **10a**—190 °C, **11a**—180 °C, **12a**—165 °C, **13a**—170 °C, **14a**—195 °C, **15a**—190 °C) for 6 h. Then, the resulting solid mixture was allowed to cool to room temperature and washed with CHCl_3_. The solid residue was dissolved in MeOH and filtered, and the solvent removed under reduced pressure. This method produced tetrachloroaurate(III) salts **8b**-**15b** with the following yields: **8b**—69%, **9b**—54%, **10b**—71%, **11b**—82%, **12b**—66%, **13b**—75%, **14b**—53%, **15b**—65%. The ^1^H, ^13^C and ^15^N NMR spectra of **8b**–**15b** are given in Supplementary Data (Figures S19–S43).

### 2.3. Studies Of Catalytic Properties of **8b**–**15b**

A mixture of catalyst (**8b**–**15b**, 0.01 mmol), benzaldehyde **19** (106 mg, i.e., 1.0 mmol), piperidine **20** (93.7 mg, i.e., 1.1 mmol) and phenylacetylene **18** (153 mg, i.e., 1.5 mmol) was stirred at 40–55 °C for 24–48 h. The yields of **19** conversions were determined by ^1^H NMR analysis of crude reaction mixtures, using 10.05 and 4.83 ppm signals integration areas.

### 2.4. Metabolic Activity Assay

The MTT (3-(4,5-dimethyldiazol-2-yl)-2,5 diphenyl tetrazolium bromide) assay, which measures activity of cellular dehydrogenases, was used to determine the cytotoxicity of new compounds [22]. In brief, cells were seeded on 96-well plates in 100 µL of culture medium and left to grow for 24 h. Tested compounds were dissolved in DMSO and diluted with the complete culture medium. To each well, 100 µL of such prepared dilution was added to obtain concentration range from 10^−7^ to 10^−3^ M. After 24 h of treatment, MTT (5 mg/mL in PBS) was added, and cells were incubated for an additional 2 h. Then, the medium was removed and insoluble blue formazan crystals were dissolved in 100 µL of DMSO. The absorbance of the formazan product was measured at 540 nm using FlexStation 3 Multi-Mode Microplate Reader (Molecular Devices, LLC., San Jose, CA, USA). The untreated cells were used as a control. The data were expressed as mean ± SEM of three independent experiments.

## 3. Results and Discussion

### 3.1. Synthesis and Characterization

Herein, we report that the C-H activation with the formation of intramolecular *sp^2^*C-*sp^2^*N bond can be conducted in solvent-free conditions, just by heating the Au(III) σ-complexes **8a**–**15a** below the particular melting point. 

**Scheme 4:**
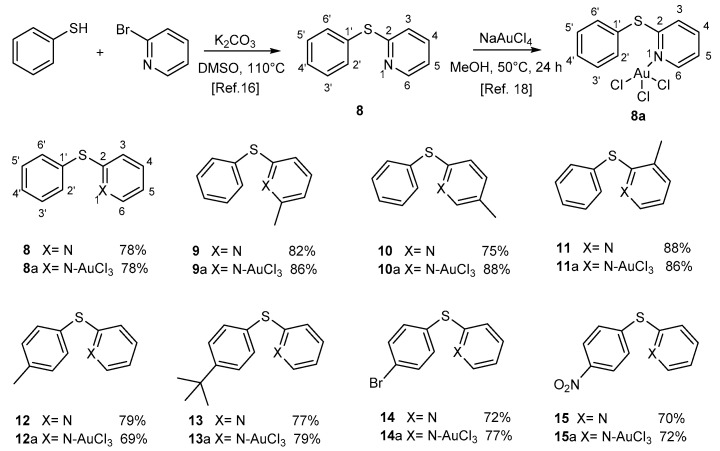
Structures of ligands **8**–**15** and Au(III) trichloride complexes **8a**–**15a**.

In the first step, we have prepared a series of 2-phenylsulfanylpyridine ligands **8**–**15** and transformed them into the Au(III) trichloride complexes **8a**–**15a** by the methodology presented previously by Fuchita et al. [24], and later modified in our research group (Scheme 4) [23]; the details of these syntheses are given below.

The species **8**, **12**–**15** and **8a**, **12a**–**15a** were already spectroscopically (^1^H, ^13^C, ^15^N NMR), and structurally (single crystal X-ray study of **8a**—DUCYEI and **14a**—DUCYIM) [21] characterized [22], while the data of **9**–**11** and **9a**–**11a** are described in Supplementary Data (Figures S1–S18).

**Scheme 5:**
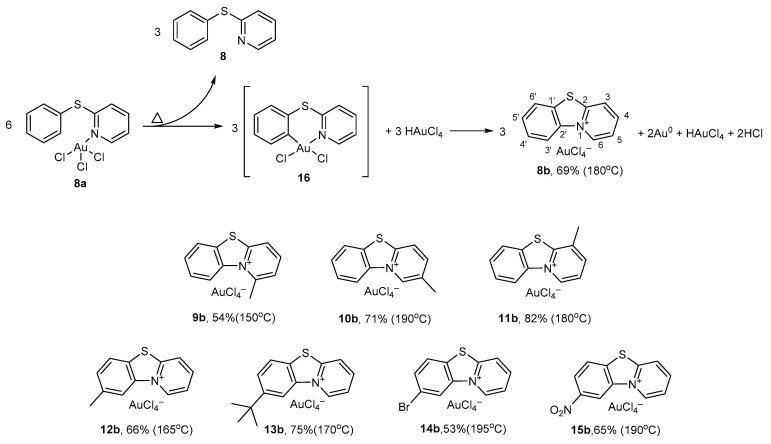
Structures of tetrachloroaurate(III) salts **8b**–**15b** containing heterocyclic cations obtained by the thermally induced C-H activation.

In the second step, the Au(III) trichloride complexes **8a**–**15a** were converted via heating to the corresponding tetrachloroaurate(III) salts **8b**–**15b**, which contain heterocyclic cations analogous to those of **6x** and **6y,** described by Kim et al. [21]. This procedure was effective for the simplest complex **8a** and its analogues, based on substrates functionalized on either the pyridine or the phenyl ring (**9a**–**11a** and **12a**–**15a**, respectively; Scheme 5). It was successfully applied in the gram-scale synthesis of **8b** (1 mmol), leading to 86% yield of the corresponding product. The spectroscopic characterization (IR, ^1^H, ^13^C, ^15^N NMR) of **8b**–**15b** is given in Supplementary Data (Figures S19–S43). We assume that the mechanism of **8a** → **8b** reaction includes the formation of the intermediate Au(III) dichloride organometallic **16** (following HCl elimination), which is transformed to the heterocyclic cation present in the tetrachloroaurate(III) salt **8b**. Moreover, a part of **8a** is also decomposed to the 2-phenylsulfanylpyridine ligand **8**. 

**Figure 1:**
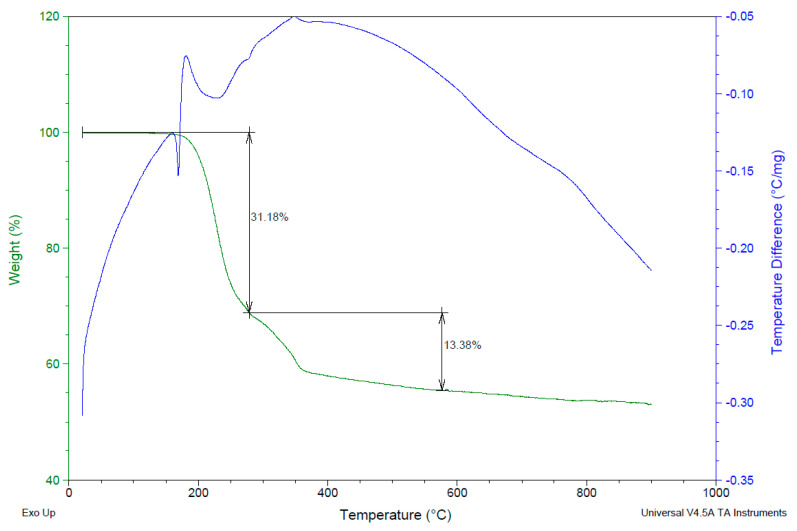
TGA–DTA analysis of the Au(III) trichloride complex **8a**.

The elimination of compound **8** was confirmed by thermogravimetric analysis TGA-DTA. Upon heating when the temperature reached ca. 180 °C, the complex **8a** decomposed in two steps. The first degradation step, ca. 31% decrease of weight, indicated the **8a** → **8** reaction. Next, at elevated temperature, the second step revealed the elimination of HAuCl_4_ species (Figure 1).

The analogous reactions **9a**–**15a** → **9b**–**15b** occur probably according to the same mechanism.

The assumed structural formulae of the cations present in the **8b**–**15b** salts are in agreement with the fully assigned (by ^1^H-^13^C and ^1^H-^15^N HMQC/HMBC techniques) NMR spectra. The respective ^1^H, ^13^C and ^15^N chemical shifts, and the comparison of these chemical shifts to those for the corresponding parent heterocycles **8**–**15,** allows us to determine the relevant differences, which reveal characteristic patterns similar for all studied cations thus, confirming their analogous structures (Tables S7–S9, part S2, Supplementary Data). 

The most characteristic phenomenon is large deshielding (by ca. 0.4–2.2 ppm) of all heterocyclic protons, expressed especially for the H(3) and H(3′) atoms (Δ^1H^ = ca. 1.6–2.2 ppm and ca. 1.3–1.7 ppm, respectively), as well as for the nitrogen-adjacent H(6) proton (Δ^1H^ = ca. 1.6–1.9 ppm); in the latter case this effect results in unusually high δ_cat_^1H^ values (>10 ppm). 

The heterocyclic carbons are either deshielded (C(3), C(4), C(5), C(2′)) or shielded (C(2), C(6), C(1′), C(3′), C(6′)), but also nearly unaffected (C(4′), C(5′)). The heterocyclic nitrogens are significantly shielded (by ca. 89–92 ppm), this effect reflecting their quaternary character (=N<^+^).

The ^1^H–^13^C long-range (via two or three bonds) correlations inside the pyridine or phenyl moieties, exhibited by the ^1^H–^13^C HMBC spectra of the Au(III) trichloride complexes **8a**–**15a** and the cations present in the tetrachloroaurate(III) salts **8b**–**15b**, are typical for such six-membered aromatic rings, and are analogous to those recently reported for the heterocycles **8** and **12**–**15 [23]** (and presently for **9**–**11**). 

However, in case of **8b** and **10b**–**15b** also, another correlation between H(6) and C(2′) atoms (*via* three bonds) is observed, exhibiting the presence of the new N(1)–C(2′) bonding (obviously, it is not observed for **9b** which has no H(6) proton); otherwise, i.e., if this additional connection between pyridine and phenyl rings was absent, there would be no possibility for any ^1^H–^3^C spin-spin interactions involving H(6) and C(2′). This H(6) –C(2′) correlation, being a crucial proof for the tricyclic structure of the cations present in **8b**–**15b** salts, has been graphically emphasized (with a circle and an arrow) at the respective ^1^H-^13^C HMBC spectra, reprinted in Supplementary Data (Figures S21, S28, S31, S34, S37, S40, S43). 

Additionally, the presence of tetrachloroaurate(III) counterions in the salts **8b**–**15b** was confirmed by far-IR spectra. One strong band at 350–355 cm^−1^ (exemplified for **8b** at Figure 2; see also Supplementary Data, Schemes S7–S14) and corresponds to the well-known asymmetric Au-Cl stretching vibration in the AuCl_4_^−^ anion [25].

Moreover, the mass spectrum of the tetrachloroaurate(III) salt **8b** exhibits the main peak at 186.1, which corresponds well to that calculated for the respective heterocyclic cation C_11_H_8_AuCl_4_NS^+^ (186.0; see Supplementary Data, Figure S22).

### 3.2. Catalytic Properties

**Scheme 6:**
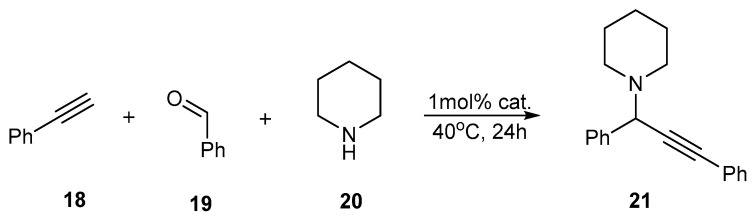
The coupling reaction between phenylacetylene **18**, benzaldehyde **19** and piperidine **20.**

In the past, some Au(III)–salen complexes and the [Au^III^(2-phenylpyridine*)Cl_2_] organometallic were exhibited to catalyze three-component coupling reactions between aldehydes, amines and alkynes with high yields; when chiral prolinol derivatives were used as substrates, excellent diastereoselectivities were reached (up to 99:1) [26,27]. Similarly, we have recently observed high catalytic activity of the Au(III) trichloride complexes **8a** and **12a**–**15a** in the coupling reaction between phenylacetylene **18**, benzaldehyde **19** and piperidine **20**, to form 1-(1,3-diphenylprop-2-yn-1-yl)piperidine **21** (yields 84–99%) (Scheme 6) [23]. 

The above facts prompted us to evaluate the catalytic properties of the presently studied tetrachloroaurate(III) salts **8b**–**15b**, in the same **18** + **19** + **20** three-component reaction; the yields at 55 °C were as high as 97–99% (Table 1). It is worth noting, that lowering the temperature to 40 °C resulted in a significant yield decrease, below 70%—as exemplified by the species **15b**. The same reaction catalyzed by sodium tetrachloroaurate(III) enabled us to obtain the final piperidine derivative, yet the yield was lower than for the used catalysts **8b**–**15b**. This indicates that the presence of the organic cation enhances the reactions efficiency.

**Scheme 7:**
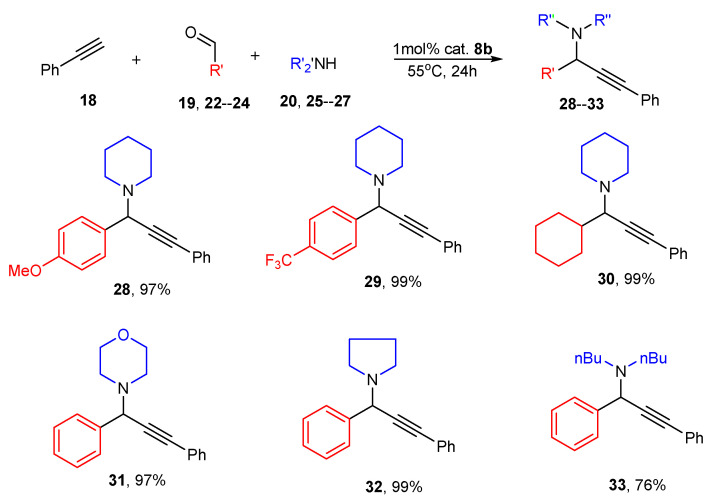
The coupling reaction between phenylacetylene **18** and adlehydes **19**, **22**–**24** and amines **20**, **25**–**27**.

In the next step, using catalyst **8b**, we tested various aldehydes (*p*-anisaldehyde **22**, 4-(trifluoromethyl) benzaldehyde **23**, cyclohexanecarboaldehyde **24**) and amines (pyrrolidine **25**, morpholine **26**, dibutylamine **27**) for three-component reactions. Corresponding products **28**–**33** were obtained with very high yields (Scheme 7).

### 3.3. Biological Activity

Due to the known anticancer properties of Au(I) and Au(III) complexes, or organometallics, e.g., with thiolates, thioureas, N-heterocyclic carbines, phosphanes and alkynes [28], the in vitro cytotoxicity of the tetrachloroaurate(III) salts **8b**–**15b** was tested against leukemia HL-60 and breast cancer MCF-7 cell lines. The cells were exposed to the increasing concentrations of analogues for 48 h and investigated using conventional MTT assay [29]. Cisplatin was used as a positive control. The most cytotoxic on both cell lines was **13b**, with the half maximal inhibitory concentration (IC_50_) of 20.9 ± 0.9 and 16.9 ± 0.8 µM on HL-60 and MCF-7 cells, respectively (Table 2). In MCF-7 cells this compound exhibited 1.4-fold higher inhibitory activity than Cisplatin. Two most cytotoxic for MCF-7 cells compounds, **12b** and **13b,** were also tested on a non-tumorigenic MCF-10A cell line, to evaluate their influence on normal cells. Analogue **12b** was equally active on MCF-7 and MCF-10A cells, while **13b** was 1.2-fold less toxic for normal than for breast cancer cells. 

## 4. Conclusions

In this article we present the first thermal C-N bond formation supported by Au(III) salts, performed in solvent-free conditions. This type of cross dehydrogenative coupling amination is one of the first examples efficiently conducted using gold complexes. The structure of the obtained compounds was confirmed by ^1^H, ^13^C, ^15^N NMR, TGA-DTA, and ATR/FT-IR analysis with a detailed discussion concerning the differences in the chemical shifts of cations in salts and their parent ligands. The mechanism of the reaction was also investigated and indicated the formation of a metallacycle as an intermediate. Additionally, all Au(III) salts were tested as catalysts in a multicomponent synthesis of propargylic amine. The reaction was almost quantitative in each case. We observed that organic cation enhanced the efficiency of the reaction. The anticancer activity was evaluated, showing a promising antiproliferative potential of the *tert*-butyl salt.

## Figures and Tables

**Figure 2:**
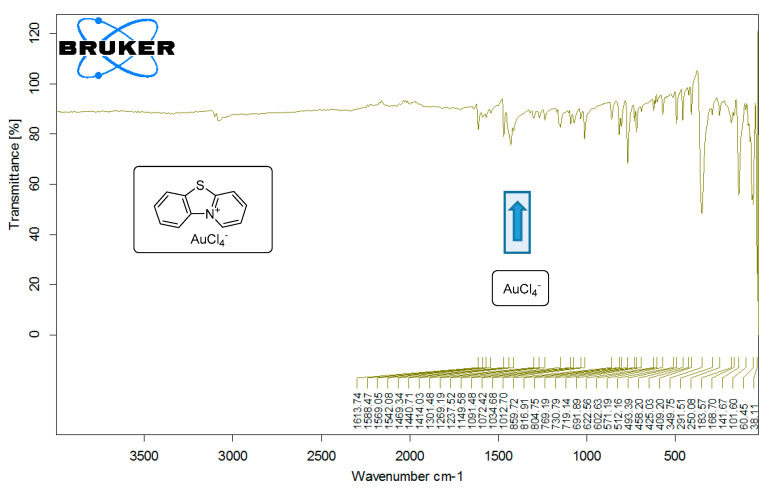
Far–IR spectrum of the tetrachloroaurate(III) salt **8b.**

**Table 1:** Yields of three-component reaction between phenylacetylene, benzaldehyde and piperidine catalyzed by tetrachloroaurate(III) salts **8b**–**15b.**

Catalyst	t [h]	T [°C]	Yield [%]
**8b**	24	55	99
**9b**	24	55	97
**10b**	24	55	97
**11b**	24	55	99
**12b**	24	55	99
**13b**	24	55	99
**14b**	24	55	98
**15b**	24	40	58
**15b**	48	40	66
**15b**	24	55	99
**NaAuCl_4_**	24	55	84

**Table 2:** Cytotoxic activity of tetrachloroaurate(III) salts **8b**–**15b.**

Compound	IC_50_ [μM] ^a^			
	HL-60	MCF-7	MCF-10A	Selectivity Index MCF-10A/MCF-7
**8b**	>100	>100		
**9b**	42.7 ± 1.4	76.9 ± 4.4		
**10b**	>100	71.6 ± 6.7		
**11b**	36.5 ± 2.2	74.0 ± 0.3		
**12b**	41.2 ± 1.8	21.3 ± 2.0	21.8 ± 2.3	1.02
**13b**	20.9 ± 0.9	16.9 ± 0.8	20.7 ± 0.5	1.20
**14b**	75.8 ± 6.8	41.5 ± 1.7		
**15b**	>100	>100		
**Cisplatin**	4.9 ± 0.1	23.6 ± 1.3		

^a^ Compound concentration required to inhibit metabolic activity by 50%. Data are expressed as the mean ± SEM from the concentration response curves of at least three experiments.

## Data Availability

Not applicable.

## Supplementary Materials

The following are available online at https://www.mdpi.com/article/10.3390/ma14071676/s1. Yields, melting points, elemental analysis, ^1^H, ^13^C, ^15^N and ^1^H-^13^C HMBC NMR spectra of A. Ligands **9**–**11** (Figures S1–S12), B. Au(III) trichloride complexes **9a**–**11a** (Figures S13–S18), C. Tetrachloroaurate(III) salts **8b**–**15b** (Figures S19–S43), and ^1^H, ^13^C and ^15^N NMR assignments for **9a**–**11a**, based on ^1^H-^13^C HSQC, HMBC and ^1^H-^15^N HMBC, and differences between ^1^H, ^1^3C or ^15^N chemical shifts for the same atom in the molecules of complexes **9a**–**11a** and ligands **9**–**11** are presented (Tables S1–S6). Additionally, ^1^H, ^13^C and ^15^N chemical shifts of salts **8b**–**15b** and parent heterocycles **8**–**15** are presented (Tables S7–S9).
