# Supplementary material for: Intramolecular C-N Bond Formation via Thermal Arene C-H Bond Activation Supported by Au(III) Complexes
Source: Materials (Basel). 2021 Mar 29;14(7):1676. doi: 10.3390/ma14071676 (PMC8036695; doi:10.3390/ma14071676)
Supplement: Supplementary file 1 [file materials-14-01676-s001.pdf]

materials

MDPI

Supplementary Materials

Intramolecular C-N Bond Formation via Thermal Arene C-H Bond Activation Supported by Au(III) Complexes

Julianna Mruk [1], Agata J. Pacuła-Miszewska [1], Leszek Pazderski [2], Joanna Drogosz-Stachowicz [3], Anna E. Janecka [3] and Jacek Ścianowski [1,*]

[1] Department of Organic Chemistry, Faculty of Chemistry, Nicolaus Copernicus University, 7 Gagarin Street, 87-100 Torun, Poland; julianna@doktorant.umk.pl (J.M.) pacula@umk.pl (A.J.P.-M.)
[2] Department of Analytical Chemistry and Applied Spectroscopy, Faculty of Chemistry, Nicolaus Copernicus University, 7 Gagarin Street, 87-100 Torun, Poland; leszek.pazderski@umk.pl
[3] Department of Biomolecular Chemistry, Faculty of Medicine, Medical University of Lodz, Mazowiecka 6/8, 92-215 Lodz, Poland; joanna.drogosz@stud.umed.lodz.pl (J.D.-S.); anna.janecka@umed.lodz.pl (A.E.J.)
* Correspondence: jsch@umk.pl

Citation: Mruk, J.; Pacuła-Miszewska, A.J.; Pazderski, L.; Drogosz-Stachowicz, J.; Janecka, A.E.; Ścianowski, J. Intramolecular C-N Bond Formation via Thermal Arene C-H Bond Activation Supported by Au(III) Complexes. *Materials* **2021**, *14*, 1676. https://doi.org/10.3390/ma14071676

Academic Editor: Daniela Iannazzo and Víctor A. de la Peña O'Shea

Received: 2 February 2021
Accepted: 23 March 2021
Published: 29 March 2021

Publisher's Note: MDPI stays neutral with regard to jurisdictional claims in published maps and institutional affiliations.

Copyright: © 2021 by the authors. Licensee MDPI, Basel, Switzerland. This article is an open access article distributed under the terms and conditions of the Creative Commons Attribution (CC BY) license (http://creativecommons.org/licenses/by/4.0/).

TABLE OF CONTENTS

`**S1.** Yields, melting points, elemental analysis, ^{1}H, ^{13}C, ^{15}N and ^{1}H-^{13}C HMBC NMR spectra of:

A. Ligands **9-11**…..……………………………………………………………………………………………..….3

B. Au(III) trichloride complexes **9a-11a**…………………………………………………………………………..11

C. Tetrachloroaurate(III) salts **8b-15b**…………………………………..……………………………………..........16

S2. ^{1}H, ^{13}C and ^{15}N chemical shifts of salts **8b-15b** and parent heterocycles **8-15** ……..33

S1. Yields, elemental analysis and ^{1}H, ^{13}C, ^{15}N NMR spectra

A. Ligands 9-11

Scheme S1. 2-phenylsulfanyl-6-methylpyridine **9**.

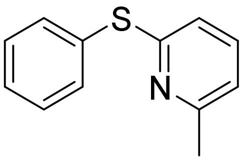

Yield: 82%; light yellow oil; Anal. Calcd for $C_{12}H_{11}NS$: C, 71.61; H, 5.51; N, 6.96. Found C, 71.91; H, 6.80; N, 7.02.

^{1}H NMR (700 MHz, $CDCl_3$) δ 7.57 (m, 2H, 2 × CH_{ar}), 7.39 (m, 1H, CH_{ar}), 7.38 (m, 2H, 2 × CH_{ar}), 7.30 (t, *J* = 7.7 Hz, 1H, CH_{ar}), 6.83 (d, *J* = 7.7 Hz, 1H, CH_{ar}), 6.59 (d, *J* = 8.4 Hz, 1H, CH_{ar}), 2.49 (s, 3H, CH_3); ^{1}H NMR (700 MHz, DMSO-d_6) δ 7.58 (m, 2H, 2 × CH_{ar}), 7.53 (t, *J* = 7.7 Hz, 1H, CH_{ar}), 7.49 (m, 3H), 7.01 (d, *J* = 7.7 Hz, 1H, CH_{ar}), 6.65 (d, *J* = 7.7 Hz, 1H, CH_{ar}), 2.40 (s, 3H, CH_3); ^{13}C NMR (101 MHz, $CDCl_3$) δ 161.0 (C_{ar}), 158.4 (C_{ar}), 137.2 (CH_{ar}), 135.0 (2 x CH_{ar}), 131.2 (C_{ar}), 129.6 (2 x CH_{ar}), 129.1 (CH_{ar}), 119.5 (CH_{ar}), 118.3 (CH_{ar}), 24.1 (CH_3); ^{13}C NMR (101 MHz, DMSO-d_6) δ 159.2 (C_{ar}), 158.2 (C_{ar}), 137.6 (CH_{ar}), 134.3 (2 x CH_{ar}), 130.6 (C_{ar}), 129.8 (2 x CH_{ar}), 129.1 (CH_{ar}), 119.8 (CH_{ar}), 118.1 (CH_{ar}), 23.7 (CH_3); ^{15}N NMR (71 MHz, $CDCl_3$) δ -79.7 ppm; ^{15}N NMR (71 MHz, DMSO-d_6) δ -78.0 ppm.

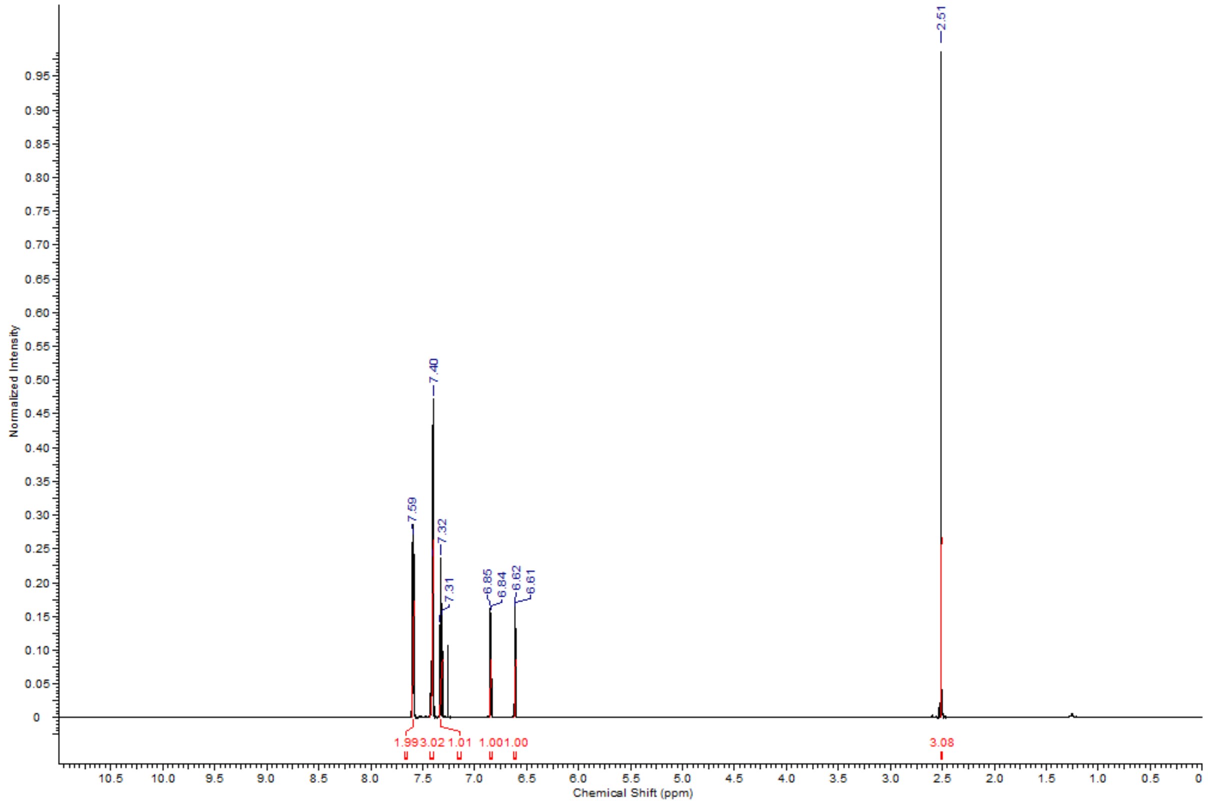

Figure S1. ^{1}H NMR spectrum of **9** in $CDCl_3$.

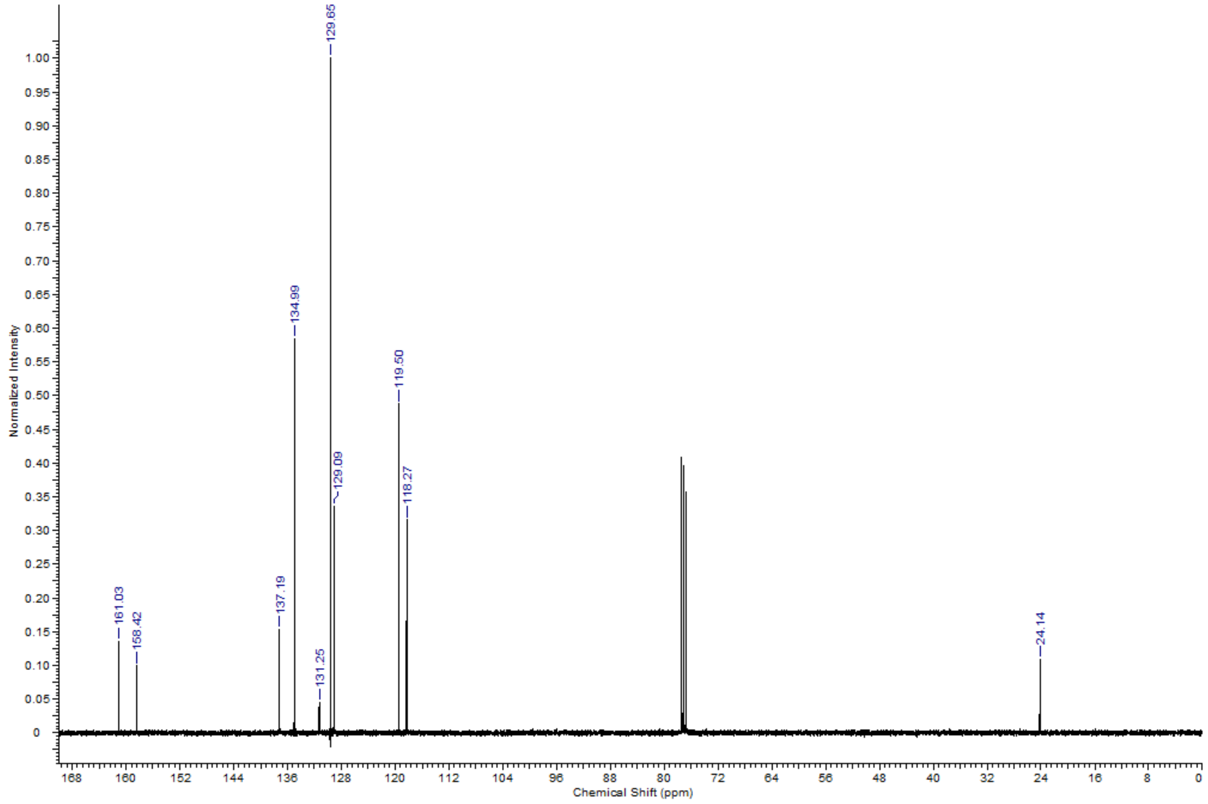

Figure S2. ^{13}C NMR spectrum of **9** in $CDCl_3$.

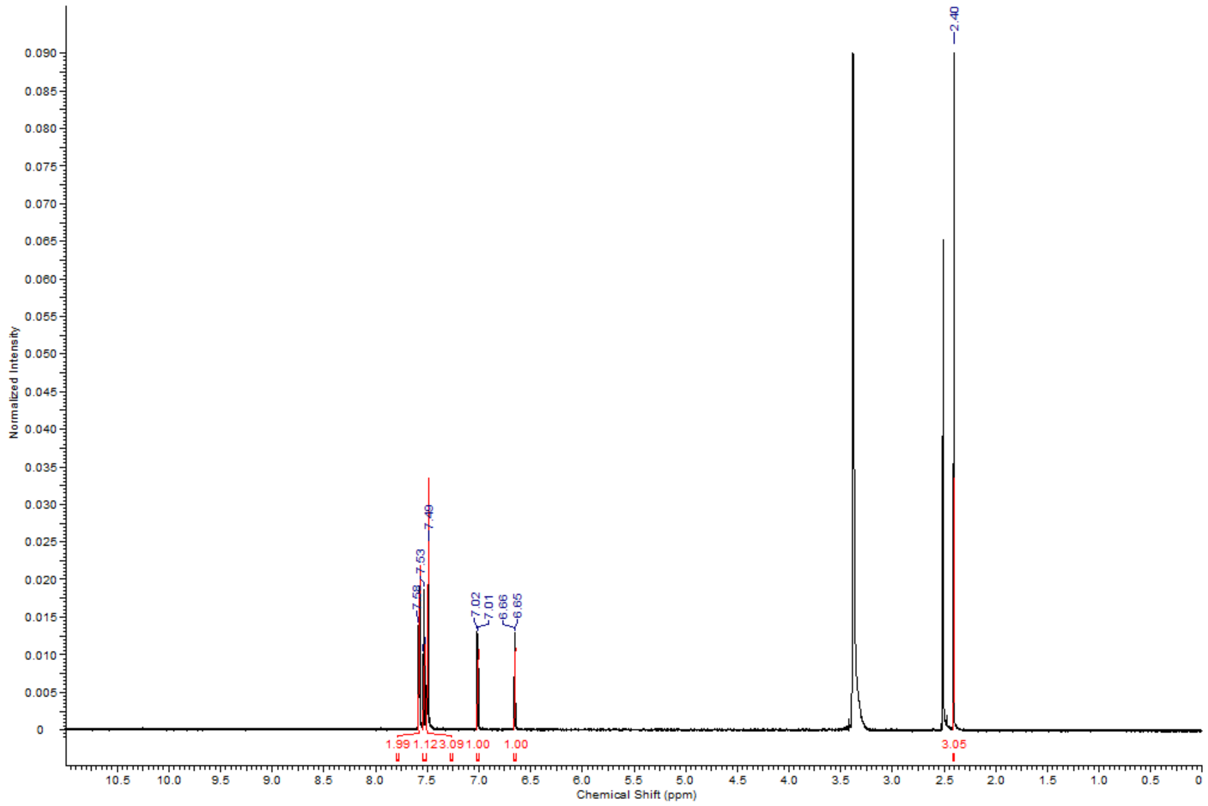

Figure S3. ^{1}H NMR spectrum of **9** in DMSO-d_6.

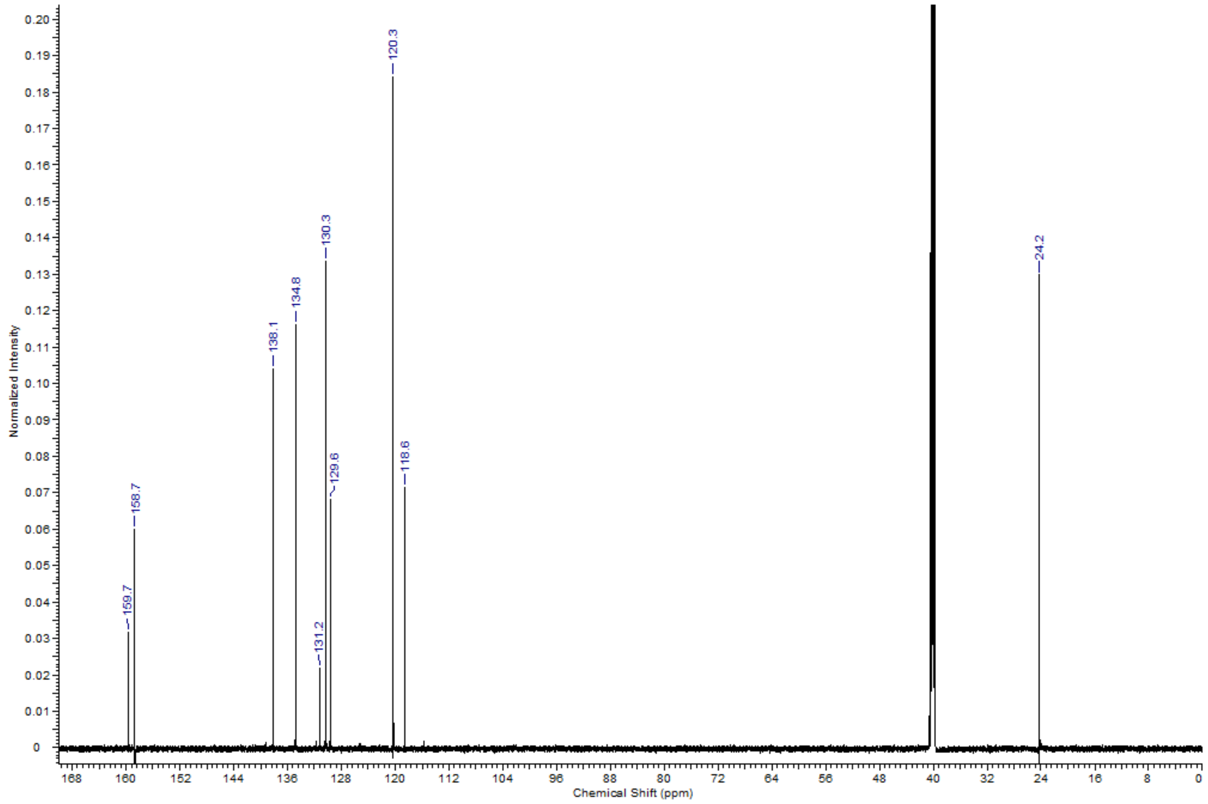

Figure S4. ^{13}C NMR spectrum of **9** in DMSO-d_6.

The ^{1}H and ^{13}C NMR assignments for **9**, based on ^{1}H-^{13}C HSQC and HMBC spectra (and confirmed by ^{1}H-^{15}N HMBC) are as follows:

^{1}H NMR of **9** in $CDCl_3$: H(3) 6.59, H(4) 7.30, H(5) 6.83, H(2′/6′) 7.57, H(3′/5′) 7.38, H(4′) 7.39, CH_3 2.49 ppm

^{13}C NMR of **9** in $CDCl_3$: C(2) 161.0, C(3) 119.5, C(4) 137.2, C(5) 118.3, C(6) 158.4, C(1′) 131.2, C(2′/6′) 135.0, C(3′/5′) 129.6, C(4′) 129.1, CH_3 24.1 ppm

^{1}H NMR of **9** in DMSO-d_6: H(3) 6.65, H(4) 7.53, H(5) 7.01, H(2′/6′) 7.58, H(3′/5′) 7.49, H(4′) 7.49, CH_3 2.40 ppm

^{13}C NMR of **9** in DMSO-d_6: C(2) 159.2, C(3) 118.1, C(4) 137.6, C(5) 119.8, C(6) 158.2, C(1′) 130.6, C(2′/6′) 134.3, C(3′/5′) 129.8, C(4′) 129.1, CH_3 23.7 ppm

Scheme S2. 2-phenylsulfanyl-5-methylpyridine **10**.

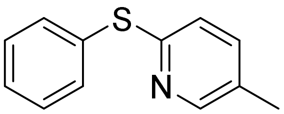

Yield: 75%; light yellow oil; Anal. Calcd for $C_{12}H_{11}NS$: C, 71.61; H, 5.51; N, 6.96. Found C, 71.44; H, 6.57; N, 7.08.

^{1}H NMR (700 MHz, $CDCl_3$) δ 8.26 (dd, *J* = 2.1, 0.7 Hz, 1H, N-CH_{ar}), 7.54 (m, 2H, 2 x CH_{ar}), 7.37 (m, 3H, 3 x CH_{ar}), 7.28 (dd, *J* = 8.4, 2.1 Hz, 1H, CH_{ar}), 6.84 (d, *J* = 7.7 Hz, 1H, CH_{ar}), 2.24 (s, 3H, CH_3) ppm; ^{1}H NMR (700 MHz, DMSO-d_6) δ 8.25 (dd, *J* = 2.1, 0.7 Hz, 1H, N-CH_{ar}), 7.51 (m, 2H, 2 x CH_{ar}), 7.49 (m, 1H, CH_{ar}), 7.45 (m, 3H, 3 x CH_{ar}), 6.90 (d, *J* = 7.7 Hz, 1H, CH_{ar}), 2.22 (s, 3H, CH_3) ppm; ^{13}C NMR (101 MHz, $CDCl_3$) δ 157.7 (C_{ar}), 149.8 (CH_{ar}), 138.1 (CH_{ar}), 134.6 (2 x CH_{ar}), 132.0 (C_{ar}), 130.1 (C_{ar}), 129.8 (2 x CH_{ar}), 129.0 (CH_{ar}), 122.1 (CH_{ar}), 18.0 (CH_3) ppm; ^{13}C NMR (101 MHz, DMSO-d_6) δ 156.0 (C_{ar}), 149.7 (CH_{ar}), 138.2 (CH_{ar}), 134.0 (2 x CH_{ar}), 131.2 (C_{ar}), 130.3 (C_{ar}), 129.8 (2 x CH_{ar}), 129.0 (CH_{ar}), 121.6 (CH_{ar}), 17.3

(CH_3) ppm; ^{15}N NMR (71 MHz, $CDCl_3$) δ -78.9 ppm; ^{15}N NMR (71 MHz, DMSO-d_6) δ -70.6 ppm.

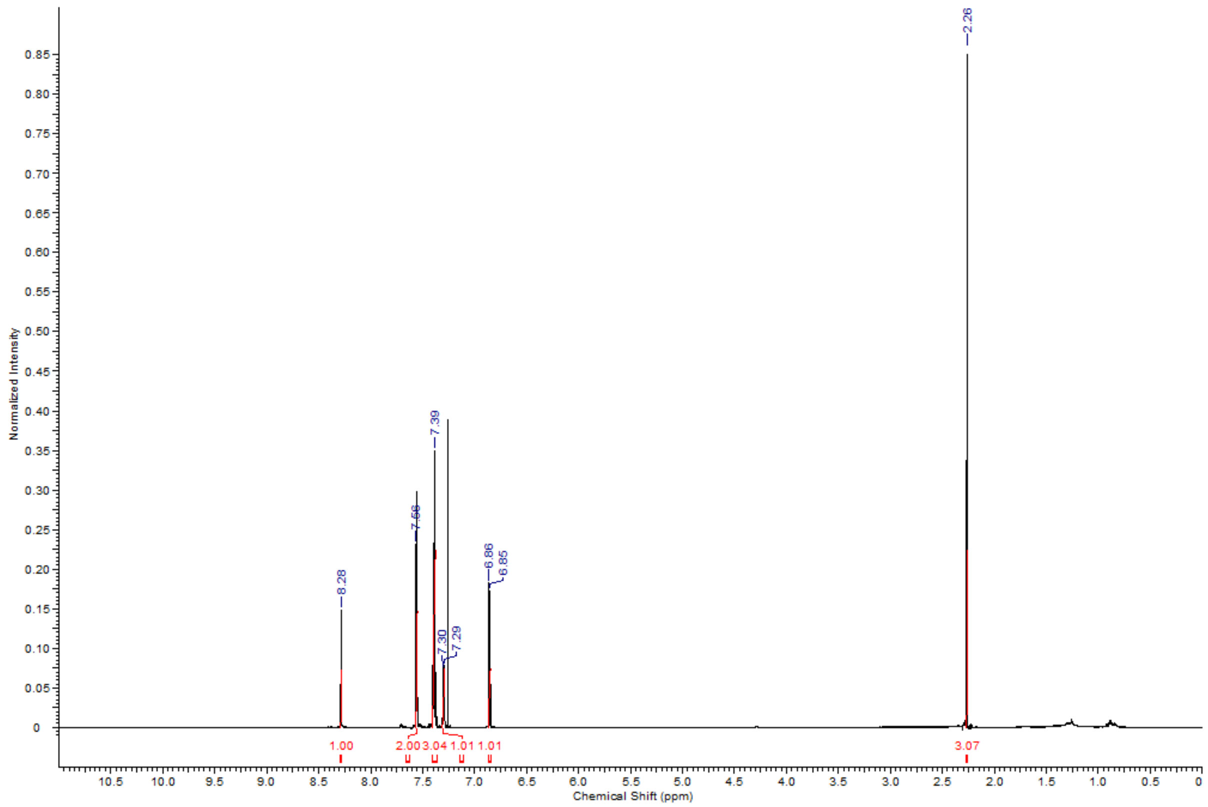

Figure S5. ^1H NMR spectrum of **10** in $CDCl_3$.

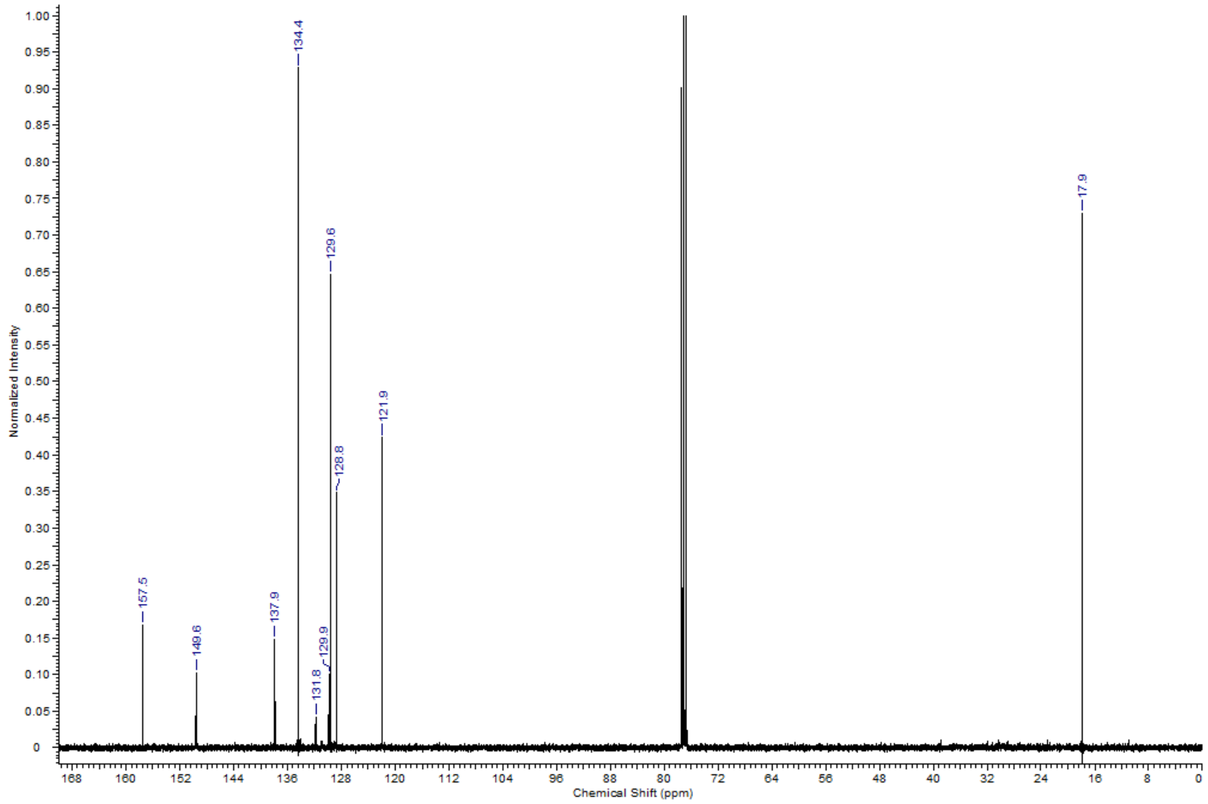

Figure S6. ^{13}C NMR spectrum of **10** in $CDCl_3$.

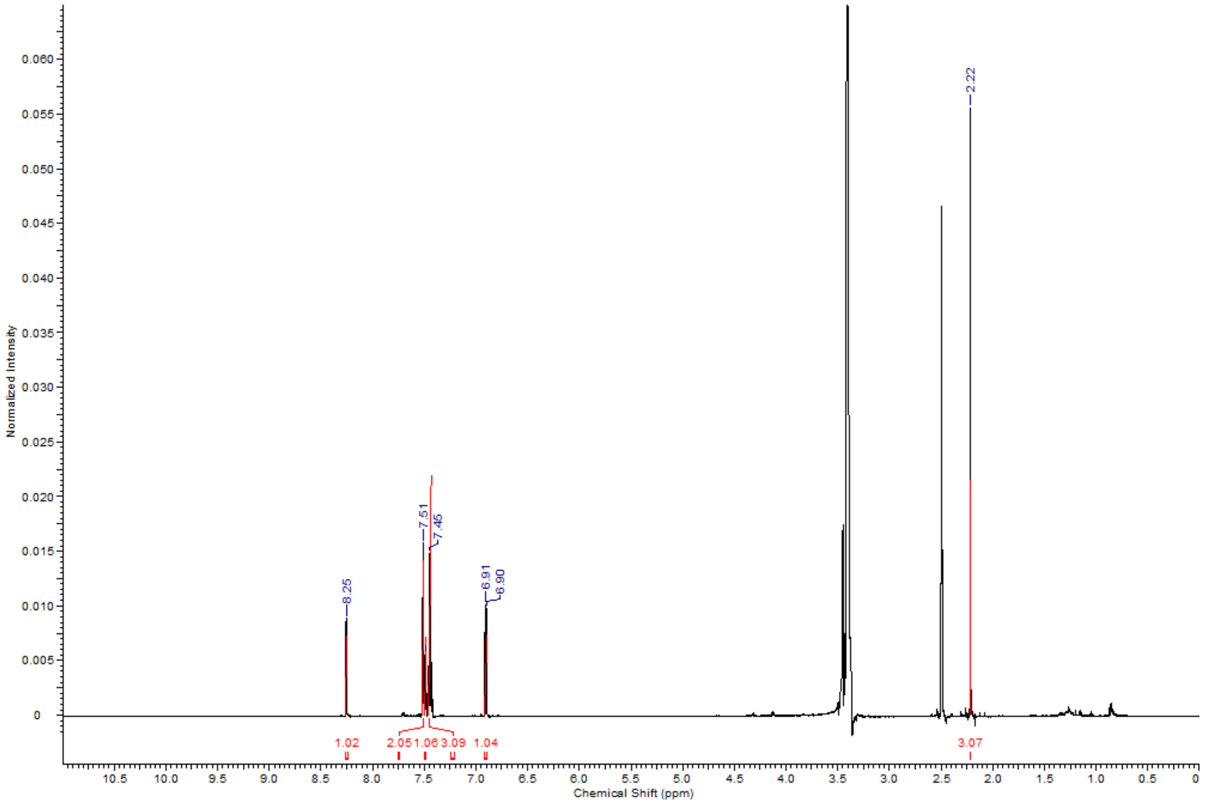

Figure S7. ^{1}H NMR spectrum of **10** in DMSO-d_6.

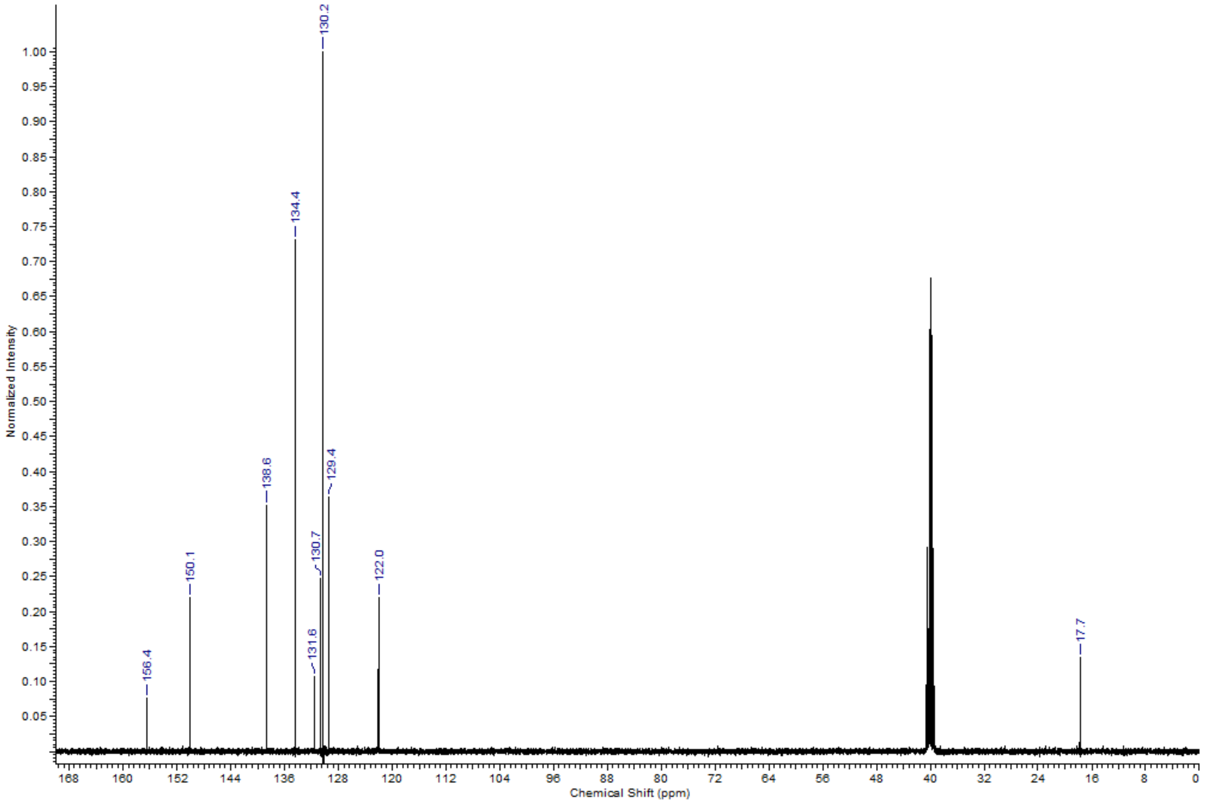

Figure S8. ^{13}C NMR spectrum of **10** in DMSO-d_6.

The ^{1}H and ^{13}C NMR assignments for **10**, based on ^{1}H-^{13}C HSQC and HMBC spectra (and confirmed by ^{1}H-^{15}N HMBC) are as follows:
^{1}H NMR of **10** in $CDCl_3$: H(3) 6.84, H(4) 7.28, H(6) 8.26, H(2′/6′) 7.54, H(3′/5′) 7.37, H(4′) 7.37, CH_3 2.24 ppm
^{13}C NMR of **10** in $CDCl_3$: C(2) 157.7, C(3) 122.1, C(4) 138.1, C(5) 130.1, C(6) 149.8, C(1′) 132.0, C(2′/6′) 134.6, C(3′/5′) 129.8, C(4′) 129.0, CH_3 18.0 ppm
^{1}H NMR of **10** in DMSO-d_6: H(3) 6.90, H(4) 7.49, H(6) 8.25, H(2′/6′) 7.51, H(3′/5′) 7.45, H(4′) 7.45, CH_3 2.22 ppm
^{13}C NMR of **10** in DMSO-d_6: C(2) 156.0, C(3) 121.6, C(4) 138.2, C(5) 130.3, C(6) 149.7, C(1′) 131.2, C(2′/6′) 134.0, C(3′/5′) 129.8, C(4′) 129.0, CH_3 17.3 ppm

Scheme S3. 2-phenylsulfanyl-3-methylpyridine **11**.

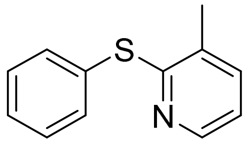

Yield: 88%; light yellow oil; Anal. Calcd for $C_{12}H_{11}NS$: C, 71.61; H, 5.51; N, 6.96. Found C, 69.87; H, 5.42; N, 7.23.
^{1}H NMR (700 MHz, $CDCl_3$) δ 8.24 (d, *J* = 4.2 Hz, 1H, N-CH_{ar}), 7.49 (m, 2H, 2 x CH_{ar}), 7.39 (m, 1H, CH_{ar}), 7.36 (m, 2H, 2 x CH_{ar}), 7.33 (m, 1H, CH_{ar}), 6.97 (dd, *J* = 7.7, 4.9 Hz, 1H, CH_{ar}), 2.35 (s, 3H, CH_3) ppm; ^{1}H NMR (700 MHz, DMSO-d_6) δ 8.15 (d, *J* = 3.5 Hz, 1H, N-CH_{ar}), 7.53 (d, *J* = 7.0 Hz, 1H, CH_{ar}), 7.45 (d, *J* = 7.0 Hz, 2H, 2 x CH_{ar}), 7.37 (m, 3H, 3 x CH_{ar}), 7.07 (dd, *J* = 7.7, 4.9 Hz, 1H, CH_{ar}), 2.28 (s, 3H, CH_3) ppm; ^{13}C NMR (101 MHz, $CDCl_3$) δ 157.5 (C_{ar}), 147.2 (CH_{ar}), 137.4 (CH_{ar}), 133.9 (2 x CH_{ar}), 132.1 (C_{ar}), 131.6 (C_{ar}), 129.1 (2 x CH_{ar}), 128.1 (CH_{ar}), 120.7 (CH_{ar}), 19.2 (CH_3) ppm; ^{13}C NMR (101 MHz, DMSO-d_6) δ 156.5 (C_{ar}), 146.9 (CH_{ar}), 137.5 (CH_{ar}), 134.2 (2 x CH_{ar}), 131.1 (C_{ar}), 130.5 (C_{ar}), 129.1 (2 x CH_{ar}), 128.3 (CH_{ar}), 120.9 (CH_{ar}), 18.3 (CH_3) ppm; ^{15}N NMR (71 MHz, $CDCl_3$) δ -73.7 ppm; ^{15}N NMR (71 MHz, DMSO-d_6) δ -72.4 ppm.

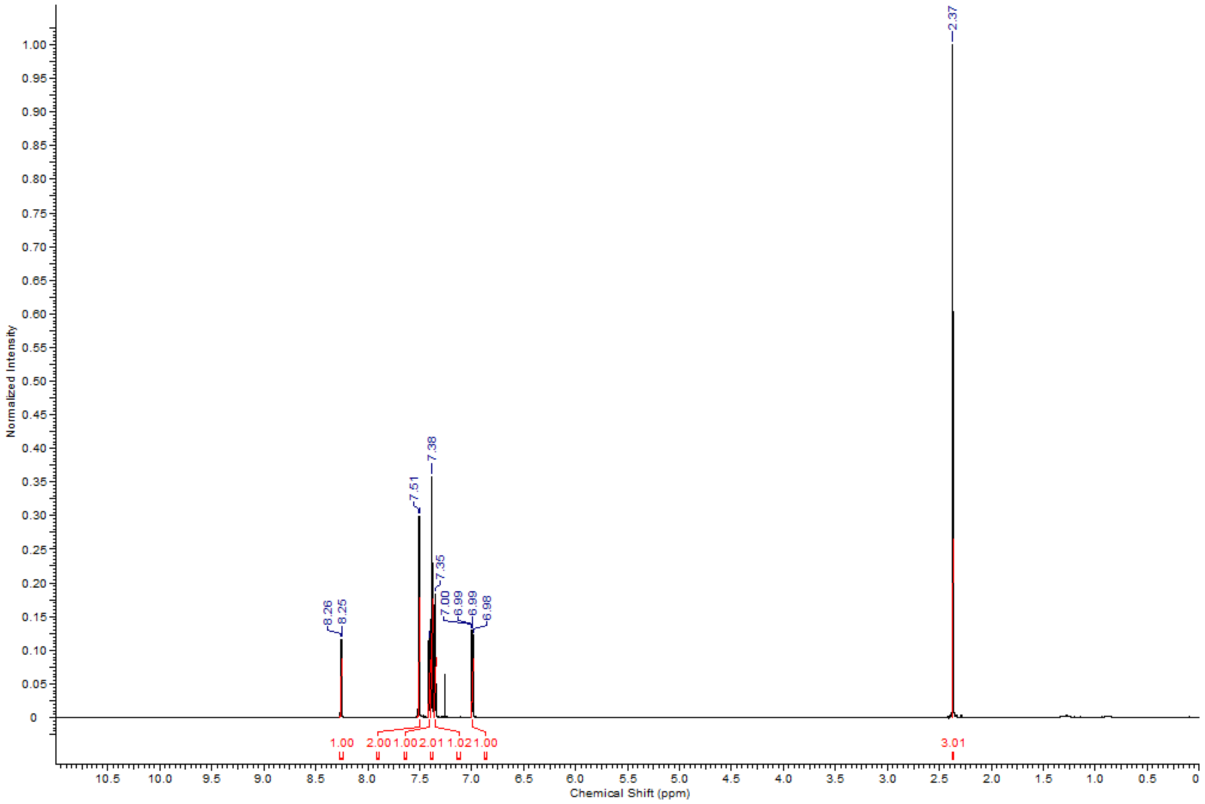

Figure S9. ^{1}H NMR spectrum of **11** in $CDCl_3$.

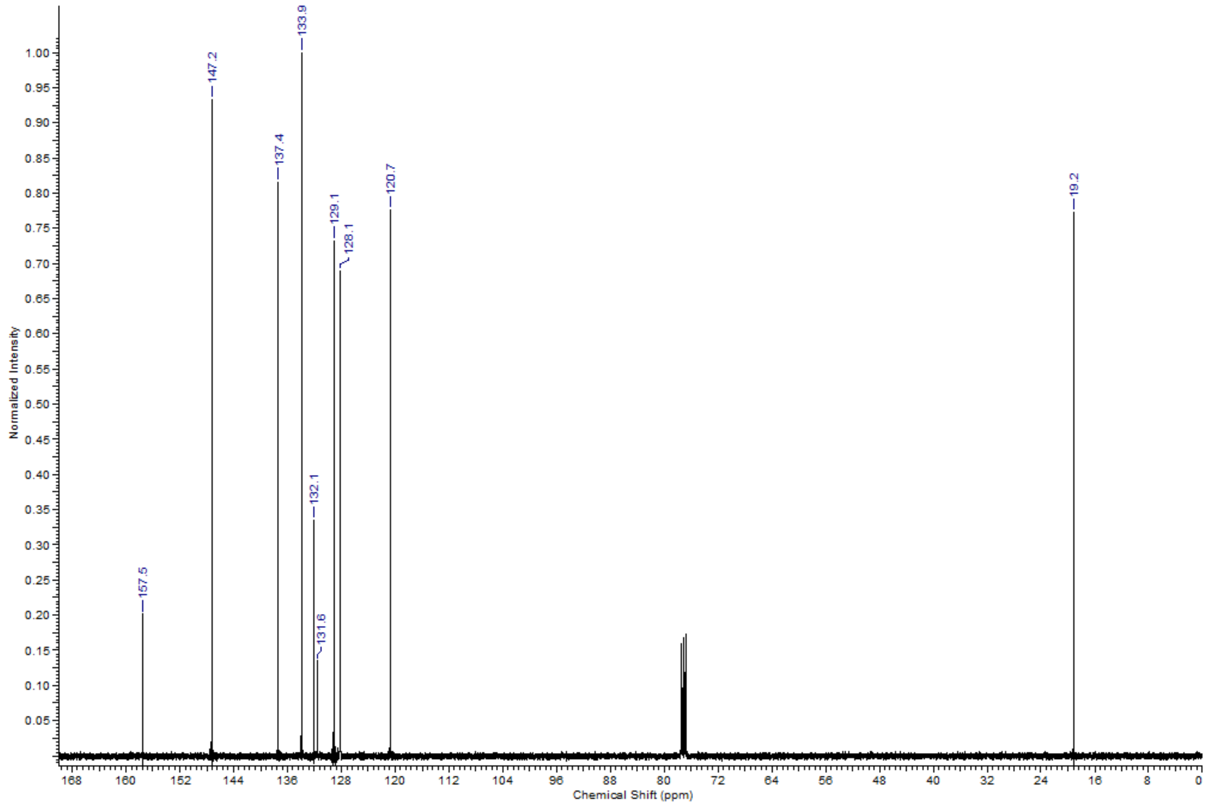

Figure S10. ^{13}C NMR spectrum of **11** in $CDCl_3$.

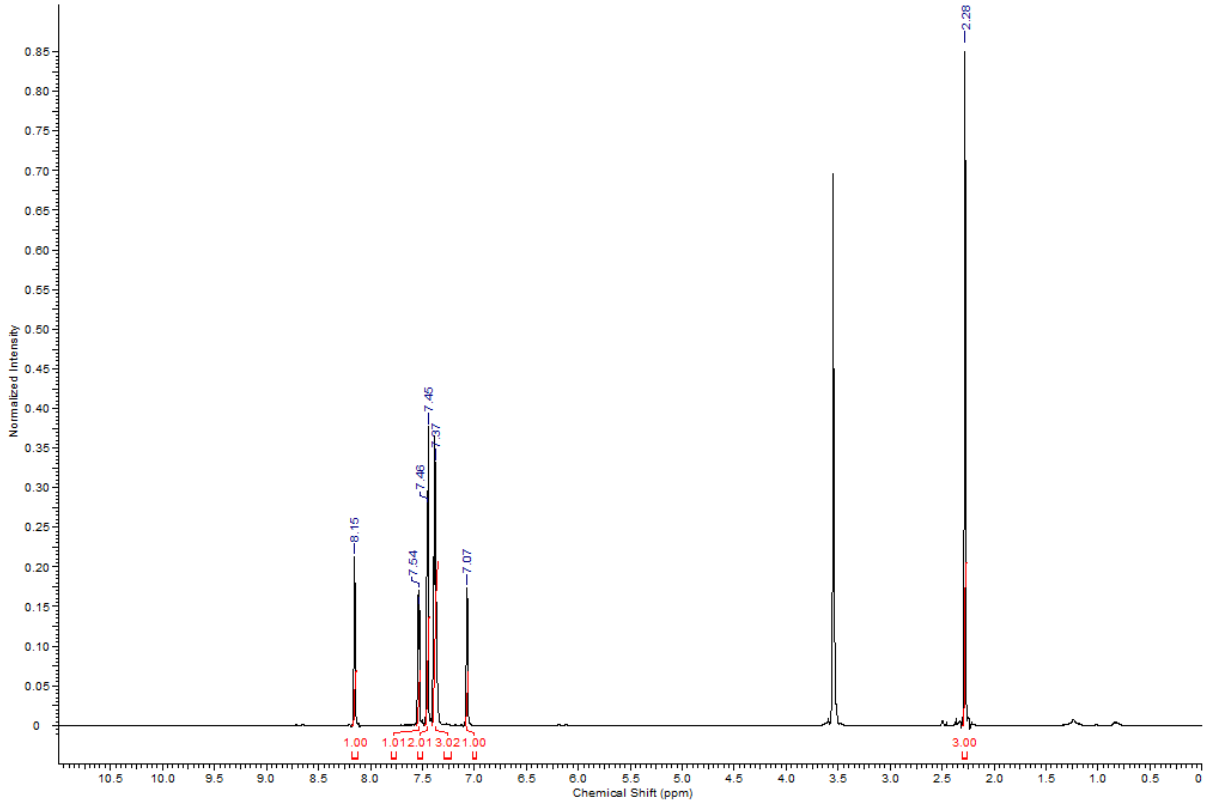

Figure S11. ^{1}H NMR spectrum of **11** in DMSO-d_6.

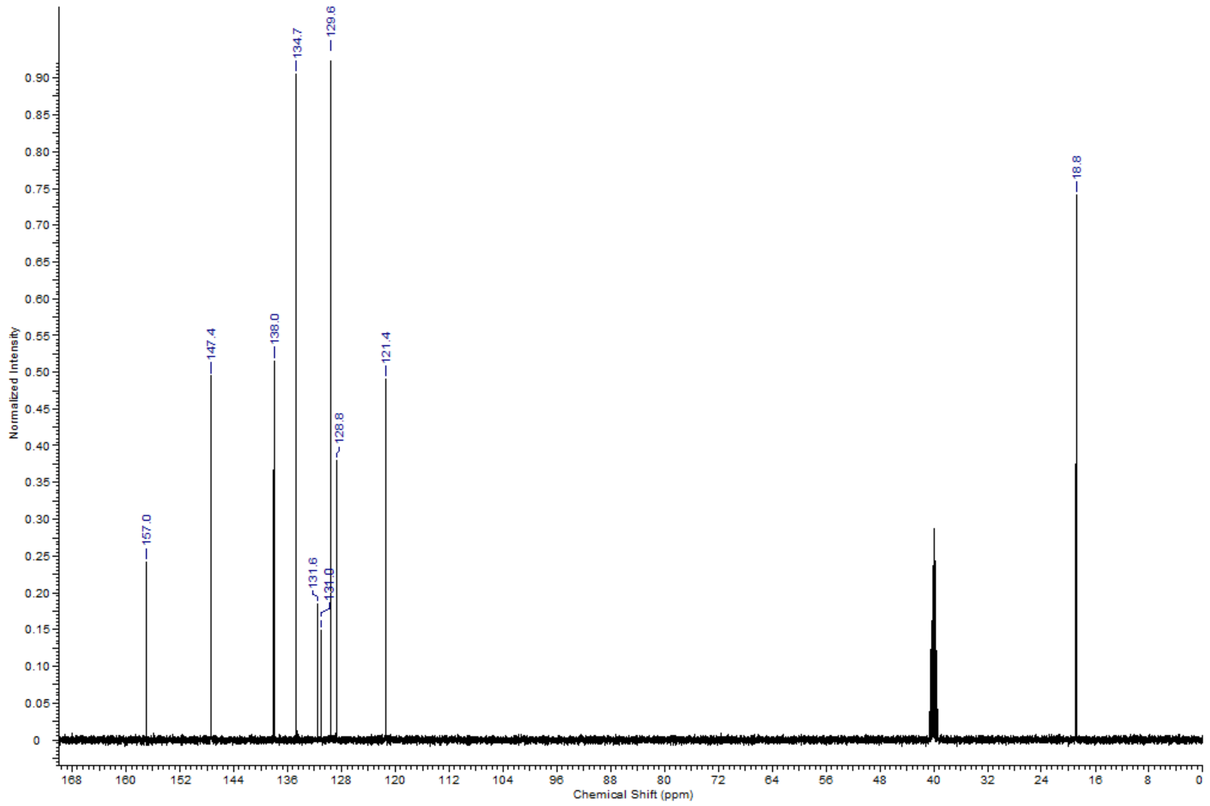

Figure S12. ^{13}C NMR spectrum of **11** in DMSO-d$_6$.

The ^{1}H and ^{13}C NMR assignments for **11**, based on ^{1}H-^{13}C HSQC and HMBC spectra (and confirmed by ^{1}H-^{15}N HMBC) are as follows:

^{1}H NMR of **11** in CDCl$_3$: H(4) 7.39, H(5) 6.97, H(6) 8.24, H(2′/6′) 7.49, H(3′/5′) 7.36, H(4′) 7.33, CH$_3$ 2.35 ppm

^{13}C NMR of **11** in CDCl$_3$: C(2) 157.5, C(3) 132.1, C(4) 137.4, C(5) 120.7, C(6) 147.2, C(1′) 131.6, C(2′/6′) 133.9, C(3′/5′) 129.1, C(4′) 128.1, CH$_3$ 19.2 ppm

^{1}H NMR of **11** in DMSO-d$_6$: H(4) 7.53, H(5) 7.07, H(6) 8.15, H(2′/6′) 7.45, H(3′/5′) 7.37, H(4′) 7.37, CH$_3$ 2.28 ppm

^{13}C NMR of **11** in DMSO-d$_6$: C(2) 156.5, C(3) 131.1, C(4) 137.5, C(5) 120.9, C(6) 146.9, C(1′) 130.5, C(2′/6′) 134.2, C(3′/5′) 129.1, C(4′) 128.3, CH$_3$ 18.3 ppm

B. Au(III) trichloride complexes 9a-11a

Scheme S4. [Au(**9**)Cl$_3$] **9a**.

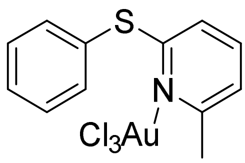

Yield: 86%; yellow powder; m.p. 137-139 °C; Anal. Calcd for $C_{12}H_{11}AuCl_3NS$: C, 28.56; H, 2.20; N, 2.78. Found C, 28.70; H, 2.53; N, 2.60.

^{1}H NMR (700 MHz, CDCl$_3$) δ 7.65 (m, 2H, 2 x CH$_{ar}$), 7.64 (m, 1H, CH$_{ar}$), 7.52 (m, 1H, CH$_{ar}$), 7.48 (m, 2H, 2 x CH$_{ar}$), 7.26 (d, *J* = 7.0 Hz, 1H, CH$_{ar}$), 6.98 (d, *J* = 8.4 Hz, 1H, CH$_{ar}$), 3.06 (s, 3H, CH$_3$); ^{13}C NMR (101 MHz, CDCl$_3$) δ 164.2 (C$_{ar}$), 158.4 (C$_{ar}$), 141.1 (CH$_{ar}$), 135.5 (2 x CH$_{ar}$), 131.4 (CH$_{ar}$), 130.8 (2 x CH$_{ar}$), 129.0 (C$_{ar}$), 125.7 (CH$_{ar}$), 125.1 (CH$_{ar}$), 26.3 (CH$_3$); ^{15}N NMR (71 MHz, CDCl$_3$) δ -149.8 ppm.

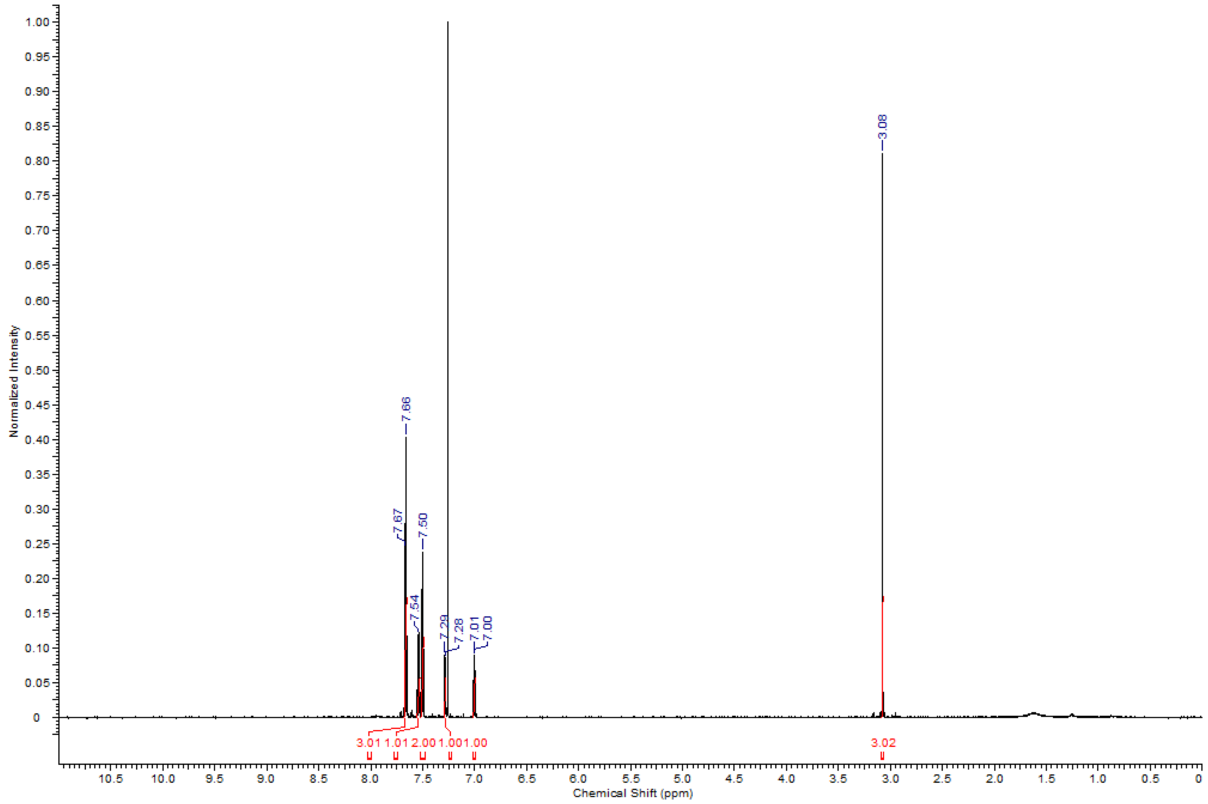

Figure S13. ^{1}H NMR spectrum of **9a** in $CDCl_3$.

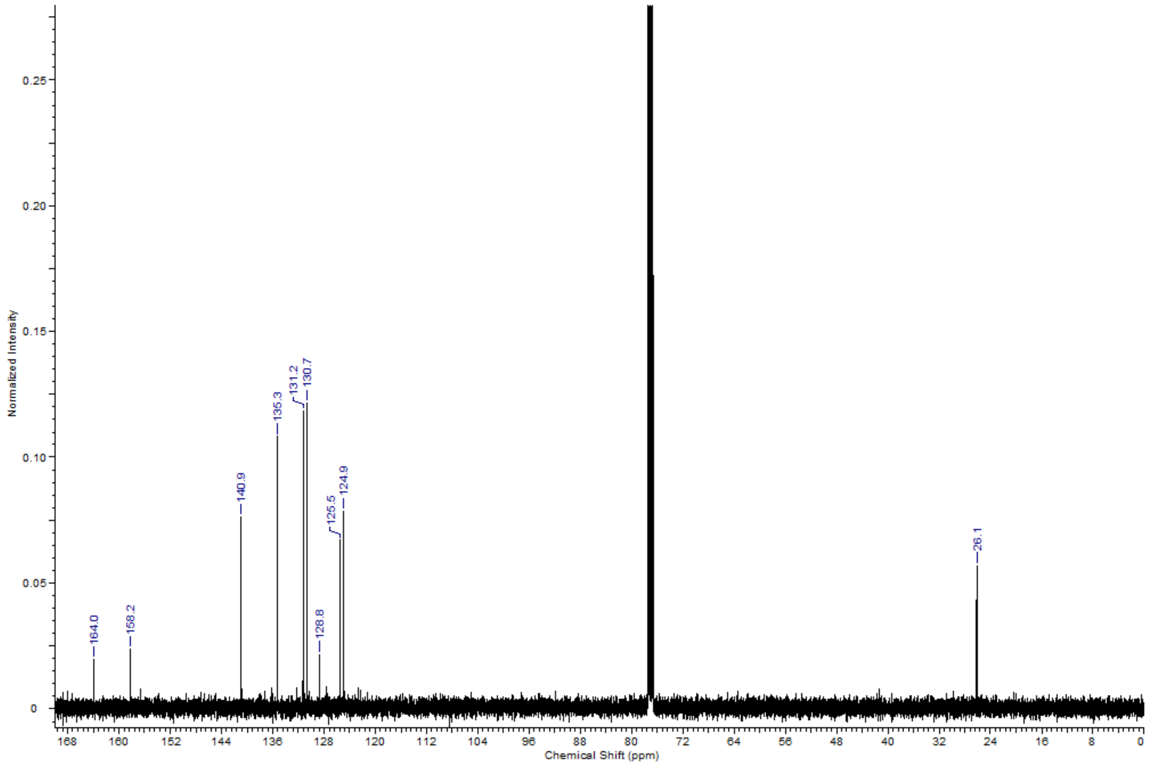

Figure S14. ^{13}C NMR spectrum of **9a** in $CDCl_3$.

Table S1. ^{1}H NMR assignments for **9a**, based on ^{1}H-^{13}C HSQC and HMBC spectra in $CDCl_3$ and differences between ^{1}H chemical shifts for the same atom in the molecules of complex **9a** and ligand **9** (in parentheses).

H(3)	H(4)	H(5)	H(2′)/H(6′)	H(3′)/H(5′)	H(4′)	CH_3
6.98	7.64	7.26	2x7.65	2x7.48	7.52	3.06
(+0.39)	(+0.34)	(+0.43)	(+0.08)	(+0.10)	(+0.13)	(+0.57)

Table S2. ^{13}C and ^{15}N NMR assignments for **9a**, based on ^{1}H-^{13}C HSQC, HMBC and ^{1}H-^{15}N HMBC spectra in $CDCl_3$, and differences between ^{13}C or ^{15}N chemical shifts for the same atom in the molecules of complex **9a** and ligand **9** (in parentheses).

C(2)	C(3)	C(4)	C(5)	C(6)	C(1′)	C(2′)/C(6′)	C(3′)/C(5′)	C(4′)	CH_3	N(1)
164.2	125.7	141.1	125.1	158.4	129.0	2x135.5	2x130.8	131.4	26.3	-149.8
(+3.2)	(+6.2)	(+3.9)	(+6.8)	(0.0)	(-2.2)	(+0.5)	(+1.2)	(+2.3)	(+2.2)	(-70.1)

Scheme S5. [Au(10)Cl_3] 10a.

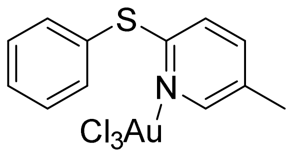

Yield: 88%; orange powder; m.p. 182-184 °C; Anal. Calcd. for $C_{12}H_{11}AuCl_3NS$: C, 28.56; H, 2.20; N, 2.78. Found C, 28.09; H, 2.50; N, 2.99.
^{1}H NMR (700 MHz, $CDCl_3$) δ 8.32 (dd, *J* = 1.4, 0.7 Hz, 1H, N-CH_{ar}), 7.63 (m, 2H, 2 x CH_{ar}), 7.55 (d, *J* = 8.4 Hz, 1H, CH_{ar}), 7.51 (m, 1H, CH_{ar}), 7.47 (m, 2H, 2 x CH_{ar}), 7.08 (d, *J* = 8.4 Hz, 1H, CH_{ar}), 2.41 (s, 3H, CH_3); ^{13}C NMR (101 MHz, $CDCl_3$) δ 160.7 (C_{ar}), 148.5 (CH_{ar}), 142.0 (CH_{ar}), 135.3 (C_{ar}), 135.2 (CH_{ar}), 131.1 (CH_{ar}), 130.6 (2 x CH_{ar}), 128.9 (C_{ar}), 128.3 (CH_{ar}), 18.2 (CH_3); ^{15}N NMR (71 MHz, $CDCl_3$) δ -153.5 ppm.

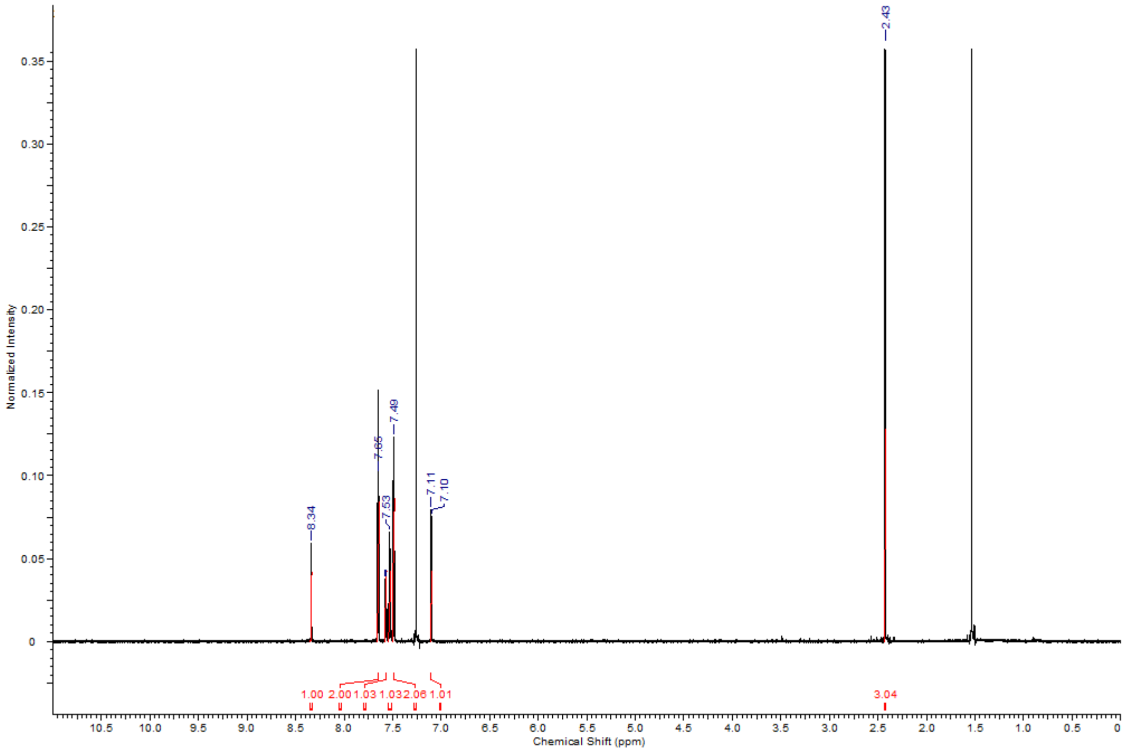

Figure S15. ^{1}H NMR spectrum of **10a** in $CDCl_3$.

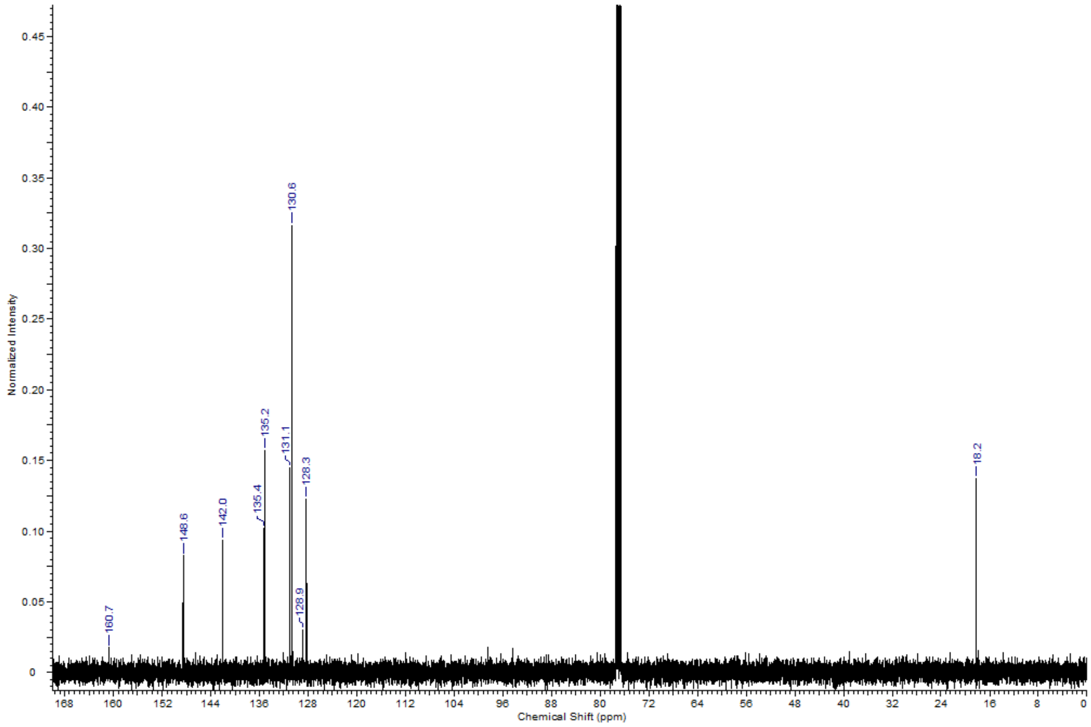

Figure S16. ^{13}C NMR spectrum of **10a** in $CDCl_3$.

Table S3. ^{1}H NMR assignments for **10a**, based on ^{1}H-^{13}C HSQC and HMBC spectra in $CDCl_3$ and differences between ^{1}H chemical shifts for the same atom in the molecules of complex **10a** and ligand **10** (in parentheses).

H(3)	H(4)	H(6)	H(2′)/H(6′)	H(3′)/H(5′)	H(4′)	CH_3
7.08	7.55	8.32	2 × 7.63	2 × 7.47	7.51	2.41
(+0.24)	(+0.27)	(+0.06)	(+0.09)	(+0.10)	(+0.14)	(+0.17)

Table S4. ^{13}C and ^{15}N NMR assignments for **10a**, based on ^{1}H-^{13}C HSQC, HMBC and ^{1}H-^{15}N HMBC spectra in $CDCl_3$, and differences between ^{13}C or ^{15}N chemical shifts for the same atom in the molecules of complex **10a** and ligand **10** (in parentheses).

C(2)	C(3)	C(4)	C(5)	C(6)	C(1′)	C(2′)/C(6′)	C(3′)/C(5′)	C(4′)	CH_3	N(1)
160.7	128.3	142.0	135.3	148.5	128.9	2v135.2	2 × 130.6	131.1	18.2	−153.5
(+3.0)	(+6.2)	(+3.9)	(+5.2)	(−1.3)	(−3.1)	(+0.6)	(+0.8)	(+2.1)	(+0.2)	(−74.6)

Scheme S6. [Au(11)Cl_3] 11a.

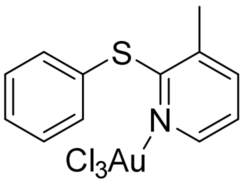

Yield: 86%; orange powder; m.p. 171-173 °C; Anal. Calcd for $C_{12}H_{11}AuCl_3NS$: C, 28.56; H, 2.20; N, 2.78. Found C, 27.96; H, 2.53; N, 3.01.

^{1}H NMR (700 MHz, $CDCl_3$) δ 8.55 (dd, *J* = 5.6, 0.7 Hz, 1H, N-CH_{ar}), 7.85 (dd, *J* = 7.7, 0.7 Hz, 1H, CH_{ar}), 7.60 (dd, *J* = 7.7, 6.3 Hz, 1H, CH_{ar}), 7.38 (m, 2H, 2 x CH_{ar}), 7.35 (m, 3H, 3 x CH_{ar}), 2.24 (s, 3H, CH_3); ^{13}C NMR (101 MHz, $CDCl_3$) δ 157.2 (C_{ar}), 148.3 (CH_{ar}), 144.3 (C_{ar}), 143.7 (CH_{ar}), 131.1 (2 x CH_{ar}), 130.5 (C_{ar}), 130.0 (2 x CH_{ar}), 129.1 (CH_{ar}), 127.2 (CH_{ar}), 21.5 (CH_3); ^{15}N NMR (71 MHz, $CDCl_3$) δ -136.6 ppm.

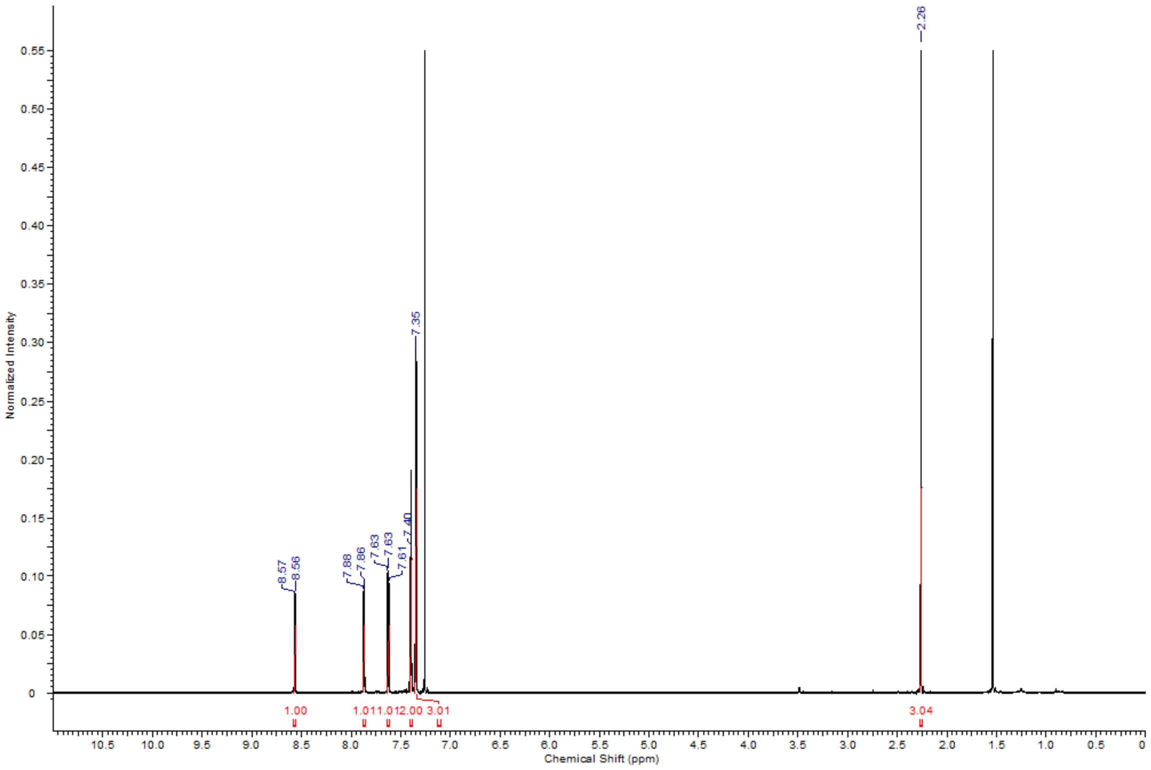

Figure S17. ^{1}H NMR spectrum of **11a** in $CDCl_3$.

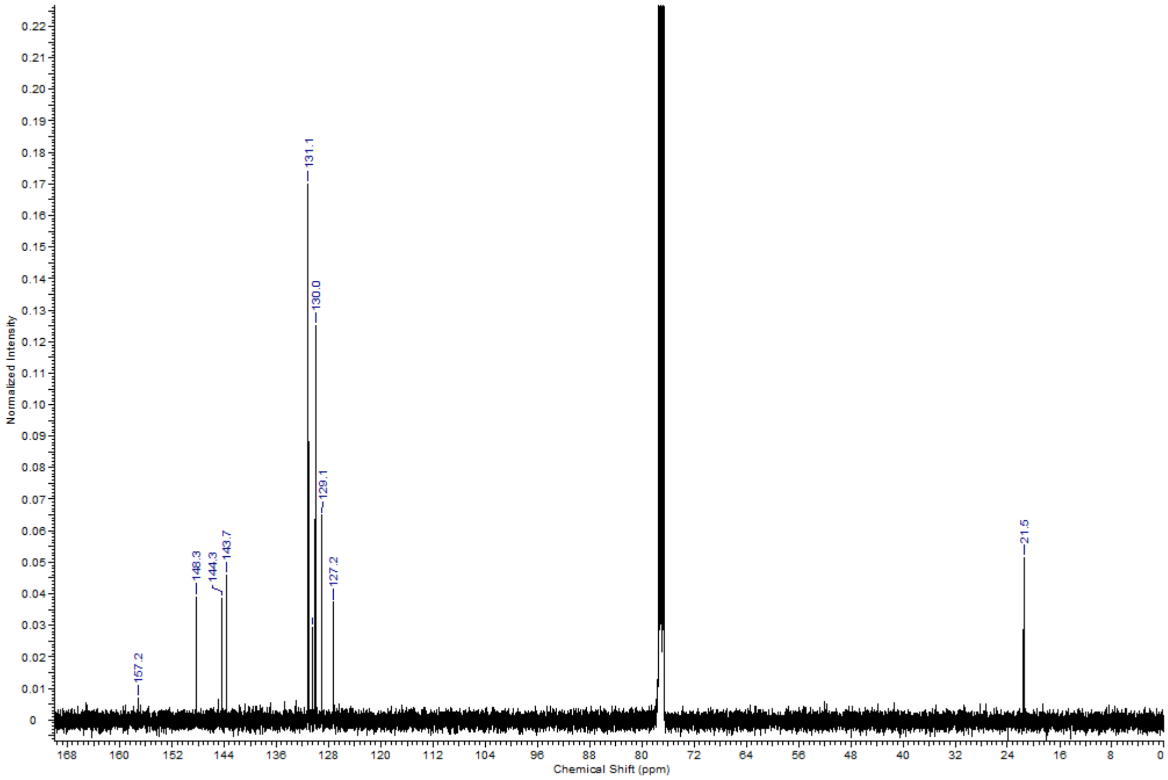

Figure S18. ^{13}C NMR spectrum of **11a** in $CDCl_3$.

Table S5. ^{1}H NMR assignments for **11a**, based on ^{1}H-^{13}C HSQC and HMBC spectra in $CDCl_3$ and differences between ^{1}H chemical shifts for the same atom in the molecules of complex **11a** and ligand **11** (in parentheses).

H(4)	H(5)	H(6)	H(2′)/H(6′)	H(3′)/H(5′)	H(4′)	CH_3
7.85	7.60	8.55	2 × 7.38	2 × 7.35	7.35	2.24
(+0.46)	(+0.63)	(+0.31)	(−0.11)	(−0.01)	(+0.02)	(−0.11)

Table S6. ^{13}C and ^{15}N NMR assignments for **11a**, based on ^{1}H-^{13}C HSQC, HMBC and ^{1}H-^{15}N HMBC spectra in $CDCl_3$, and differences between ^{13}C or ^{15}N chemical shifts for the same atom in the molecules of complex **11a** and ligand **11** (in parentheses).

C(2)	C(3)	C(4)	C(5)	C(6)	C(1′)	C(2′)/C(6′)	C(3′)/C(5′)	C(4′)	CH_3	N(1)
157.2	144.3	143.7	127.2	148.3	130.5	2 × 131.1	2 × 130.0	129.1	21.5	−136.6
(−0.3)	(+12.2)	(+6.3)	(+6.5)	(+1.1)	(−1.1)	(−2.8)	(+0.9)	(+1.0)	(+2.3)	(−62.9)

*C. Tetrachloroaurate(III) salts **8b-15b***

Scheme S7. Benzo[4,5]thiazolo[3,2-α]pyridin-10-ium tetrachloroaurate(III) **8b.**

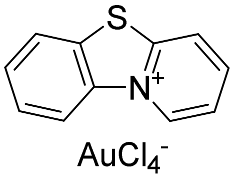

Yield: 69%; yellow powder; m.p. 257-259 °C; IR (ATR) $\tilde{\upsilon}$ 351 cm^{-1}; MS (ESI): Mass calcd for [M$^+$] $C_{11}H_8AuCl_4NS^+$: 186.0; found 186.1. Anal. Calcd for $C_{11}H_8AuCl_4NS$: C, 25.17; H, 1.54; N, 2.67; Cl, 27.01. Found C, 25.61; H, 1.99; N, 2.76; Cl, 24.56.

^{1}H NMR (700 MHz, DMSO-d_6) δ 10.10 (d, *J* = 7.0 Hz, 1H, N-CH_{ar}), 8.94 (m, 1H, CH_{ar}), 8.88 (m, 1H, CH_{ar}), 8.53 (m, 1H, CH_{ar}), 8.52 (m, 1H, CH_{ar}), 8.16 (m, 1H, CH_{ar}), 7.96 (m, 1H, CH_{ar}), 7.94 (m, 1H, CH_{ar}) ppm; ^{13}C NMR (101 MHz, DMSO-d_6) δ 154.3 (C_{ar}), 140.0 (CH_{ar}), 136.7 (C_{ar}), 135.1 (CH_{ar}), 130.3 (CH_{ar}), 129.1 (C_{ar}), 128.5 (CH_{ar}), 124.9 (CH_{ar}), 124.2 (CH_{ar}), 122.3 (CH_{ar}), 116.7 (CH_{ar}) ppm; ^{15}N NMR (71 MHz, DMSO-d_6) δ -168.9 ppm.

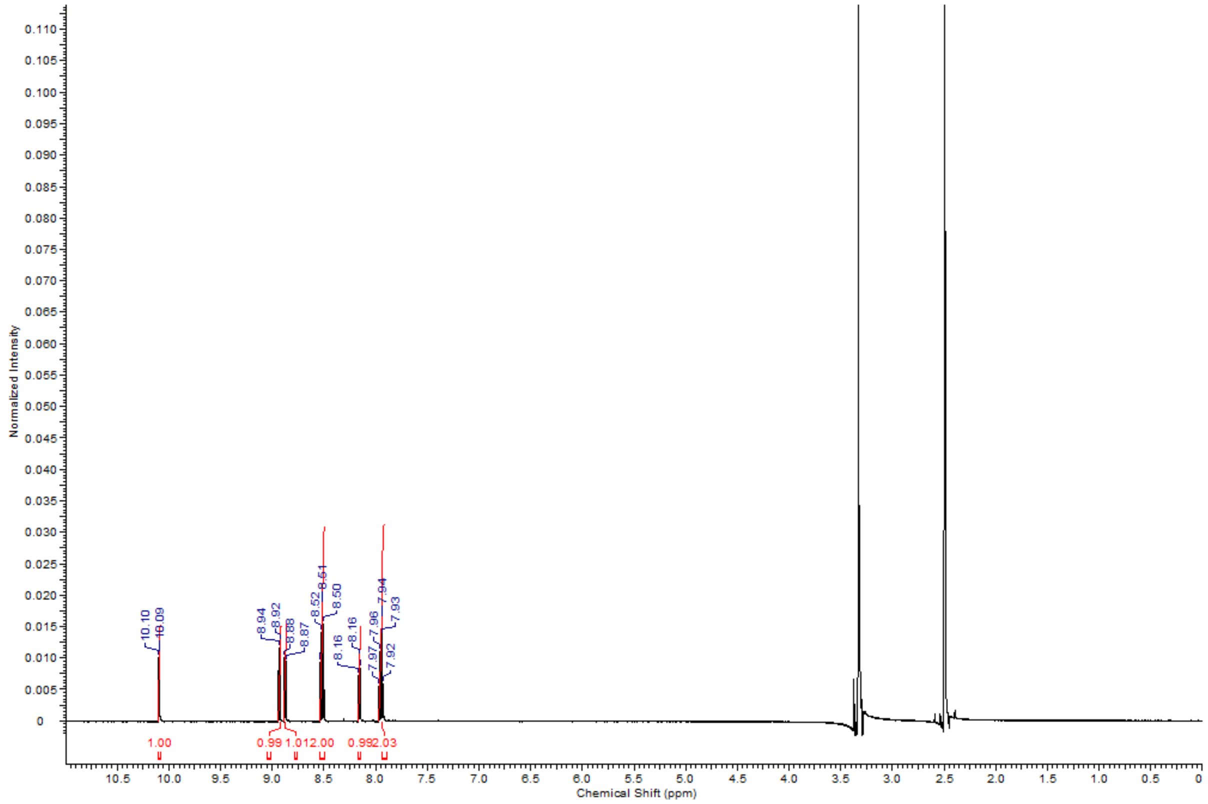

Figure S19. ¹H NMR spectrum of **8b** in DMSO-d6.

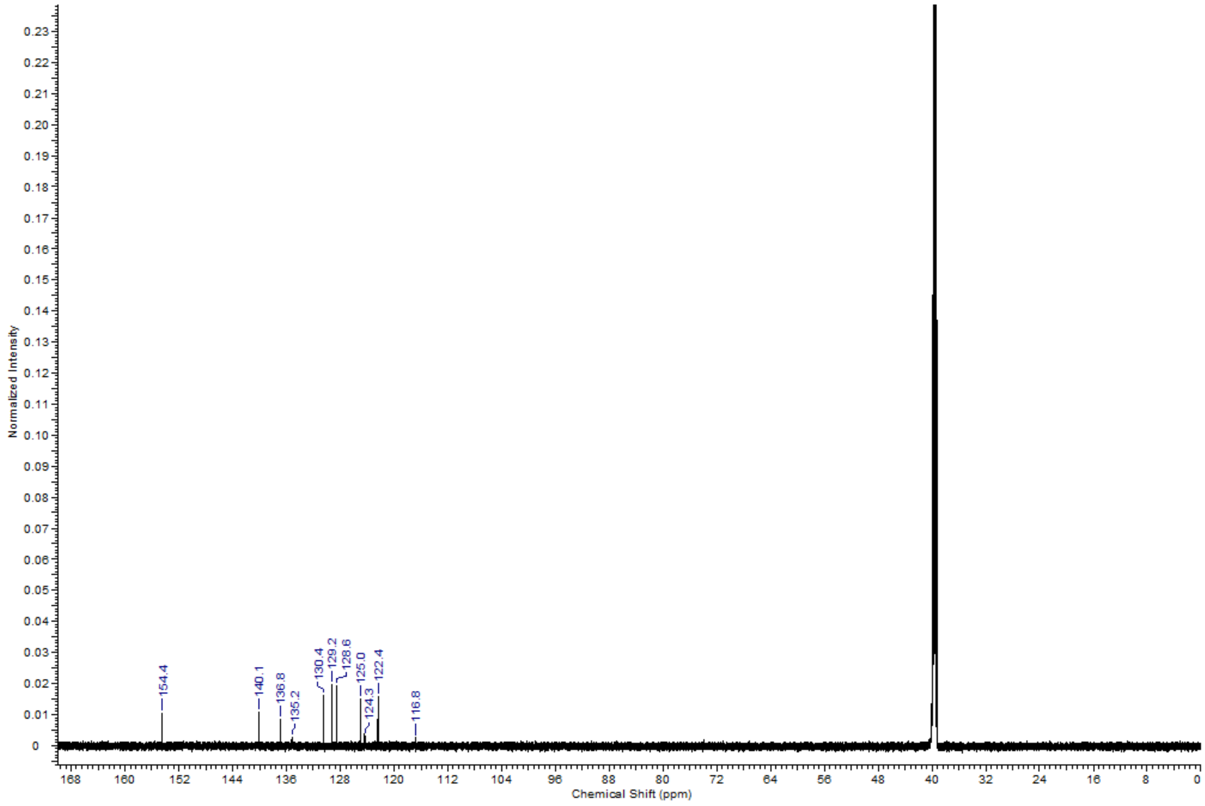

Figure S20. ¹³C NMR spectrum of **8b** in DMSO-d6.

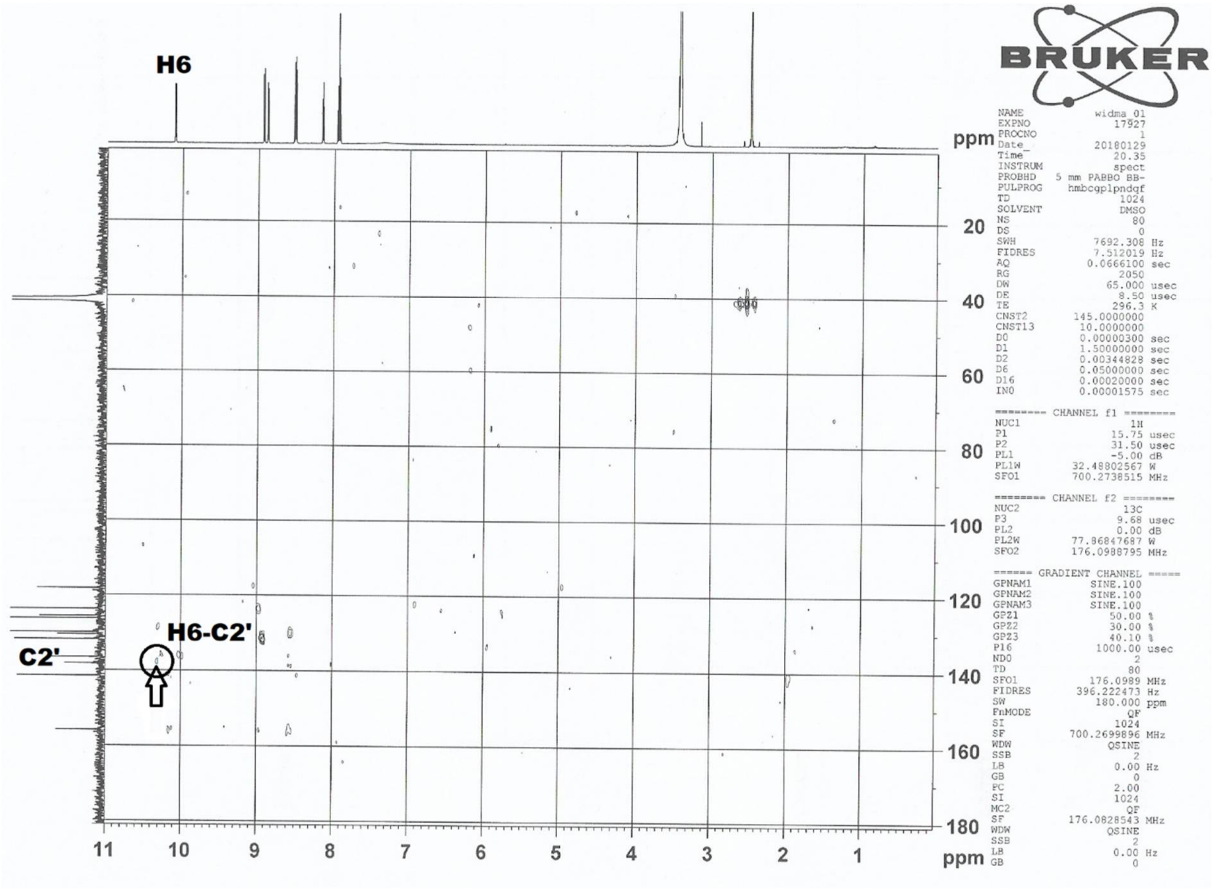

Figure S21. ^1H-^{13}C HMBC NMR spectrum of **8b** in DMSO-d$_6$.

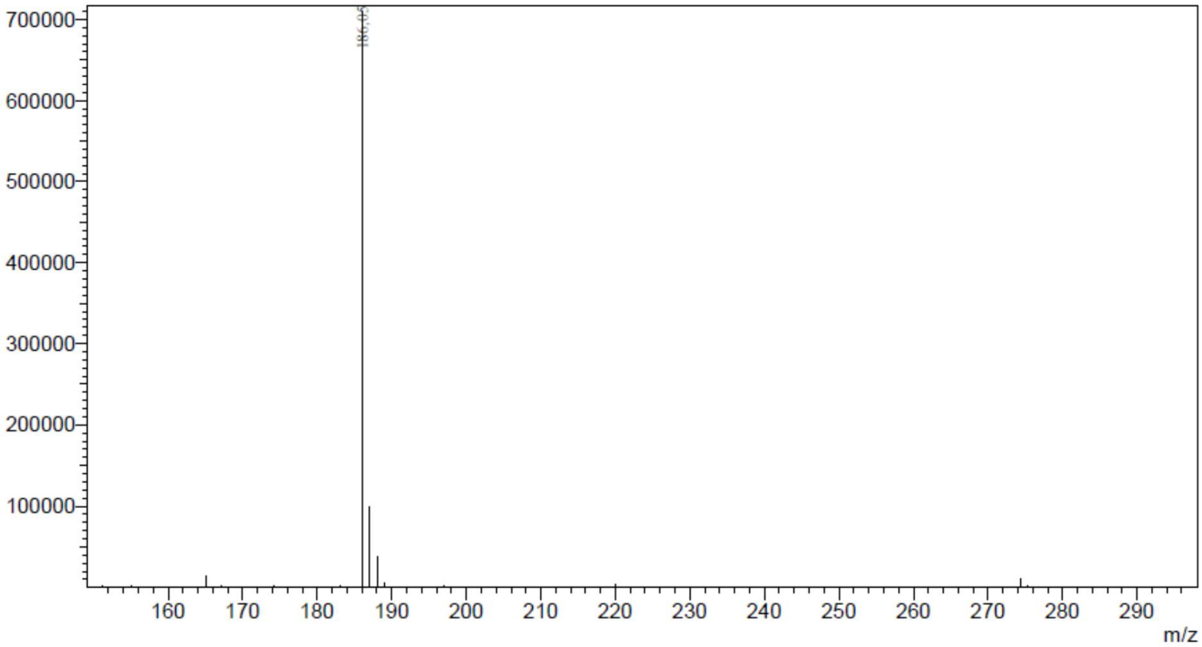

Figure S22. Mass spectrum of **8b.**

Scheme S8. 1-Methylbenzo[4,5]thiazolo[3,2-*a*]pyridin-10-ium tetrachloroaurate(III) **9b.**

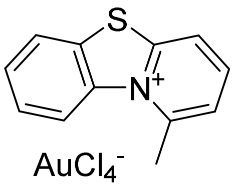

Yield: 54%; brown powder; m.p. 180-182 °C; IR (ATR) $\tilde{\upsilon}$ 355 cm^{-1}; Anal. Calcd for $C_{12}H_{10}AuCl_4NS$: C, 26.74; H, 1.87; N, 2.60. Found C, 27.09; H, 2.05; N, 2.45.
^1H NMR (700 MHz, DMSO-d_6) δ 8.90 (d, *J* = 8.4 Hz, 1H, CH_{ar}), 8.82 (dd, *J* = 8.4, 0.7 Hz, 1H, CH_{ar}), 8.53 (m, 1H, CH_{ar}), 8.41 (dd, *J* = 8.4, 7.7 Hz, 1H, CH_{ar}), 7.99 (m, 1H, CH_{ar}), 7.93 (m, 1H, CH_{ar}), 7.88 (m, 1H, CH_{ar}), 3.40 (s, 3H, CH_3) ppm; ^{13}C NMR (101 MHz, DMSO-d_6) δ 155.2 (C_{ar}), 150.9 (C_{ar}), 139.2 (CH_{ar}), 138.5 (C_{ar}), 129.7 (CH_{ar}), 129.3 (C_{ar}), 128.2 (CH_{ar}), 125.4 (CH_{ar}), 124.6 (CH_{ar}), 121.9 (CH_{ar}), 121.6 (CH_{ar}), 24.0 (CH_3) ppm; ^{15}N NMR (71 MHz, DMSO-d_6) δ -167.6 ppm.
^1H and ^{13}C NMR assignments are in Table 1 and 2 in the main text.

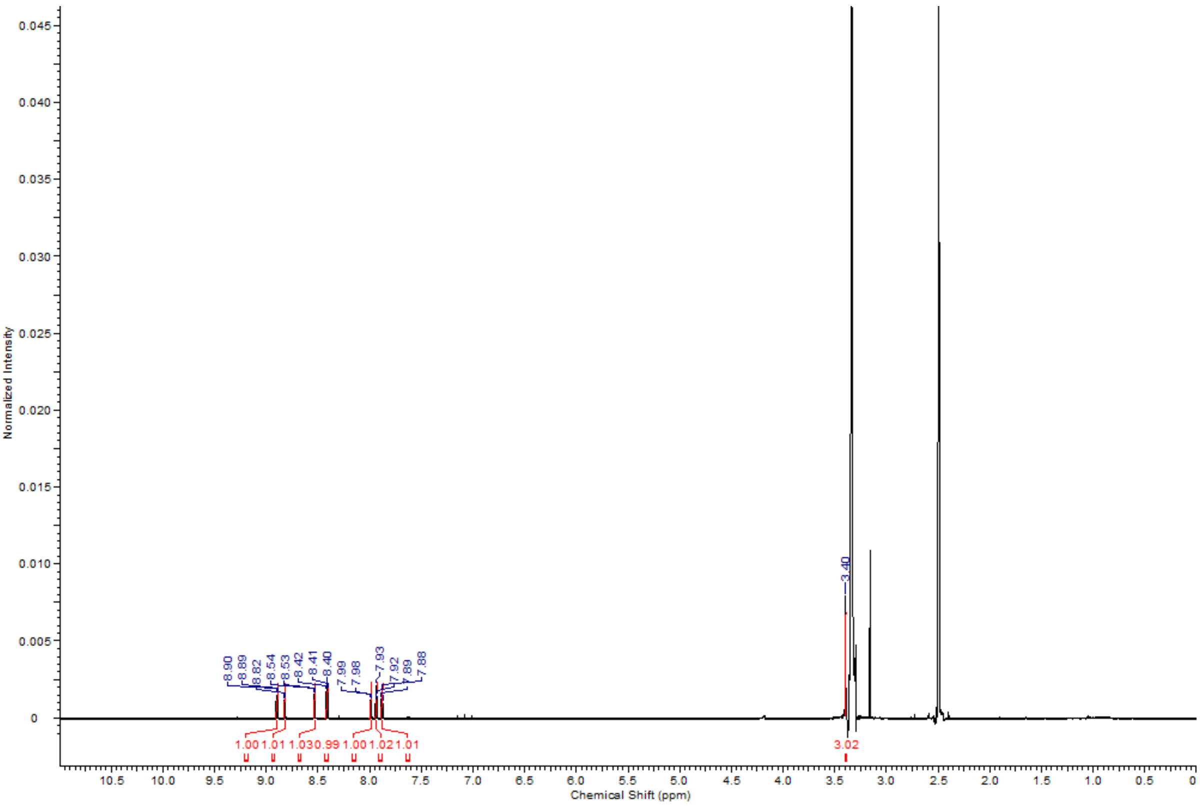

Figure S23. ^1H NMR spectrum of **9b** in DMSO-d_6.

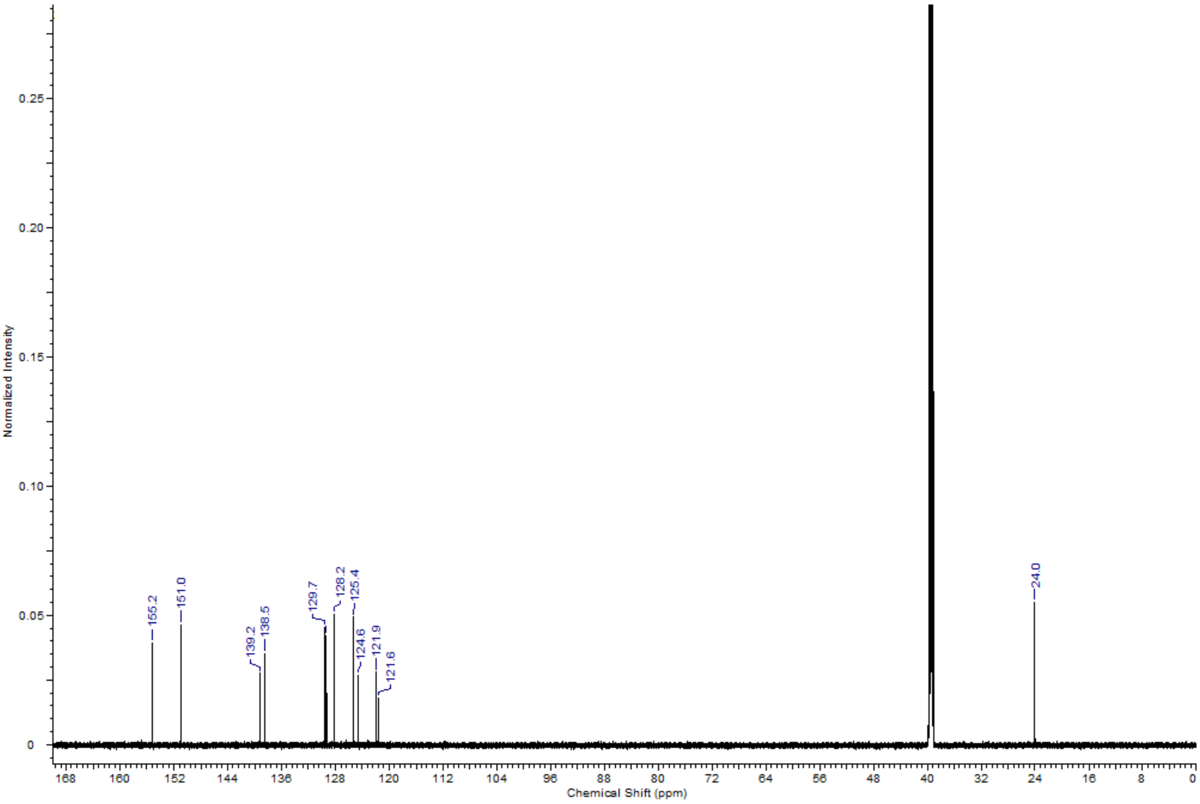

Figure S24. ^{13}C NMR spectrum of **9b** in DMSO-d$_6$.

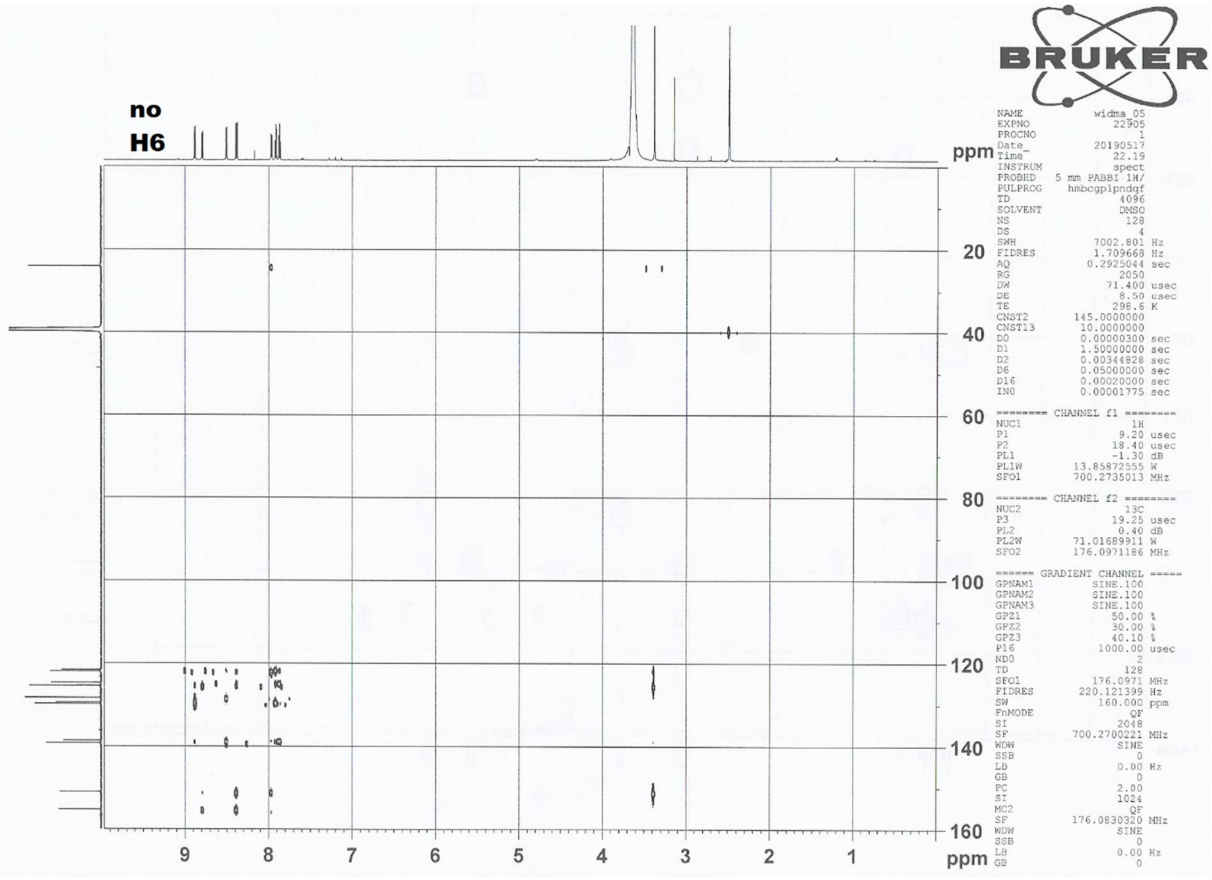

Figure S25. ^{1}H-^{13}C HMBC NMR spectrum of **9b** in DMSO-d$_6$.

Scheme S9. 2-Methylbenzo[4,5]thiazolo[3,2-*a*]pyridin-10-ium tetrachloroaurate(III) **10b.**

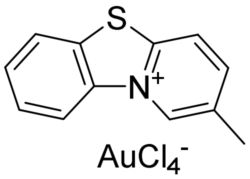

Yield: 71%; yellow powder; m.p. 147-149 °C; IR (ATR) $\tilde{\upsilon}$ 354 cm^{-1}; Anal. Calcd for $C_{12}H_{10}AuCl_4NS$: C, 26.74; H, 1.87; N, 2.60. Found C, 27.93; H, 1.96; N, 2.60.
^{1}H NMR (700 MHz, DMSO-d_6) δ 10.01 (s, 1H, N-CH_{ar}), 8.83 (dd, *J* = 8.4, 5.6 Hz, 2H, 2 x CH_{ar}), 8.49 (m, 1H, CH_{ar}), 8.41 (m, 1H, CH_{ar}), 7.95 (m, 1H, CH_{ar}), 7.91 (m, 1H, CH_{ar}), 2.62 (s, 3H, CH_3) ppm; ^{13}C NMR (101 MHz, DMSO-d_6) δ 151.7 (C_{ar}), 141.8 (CH_{ar}), 136.5 (C_{ar}), 133.5 (CH_{ar}), 133.1 (C_{ar}), 130.2 (CH_{ar}), 129.3 (C_{ar}), 128.4 (CH_{ar}), 124.9 (CH_{ar}), 123.3 (CH_{ar}), 116.5 (CH_{ar}), 17.6 (CH_3) ppm; ^{15}N NMR (71 MHz, DMSO-d_6) δ -168.1 ppm.

^{1}H and ^{13}C NMR assignments are in Tab. 1-2 in the main text.

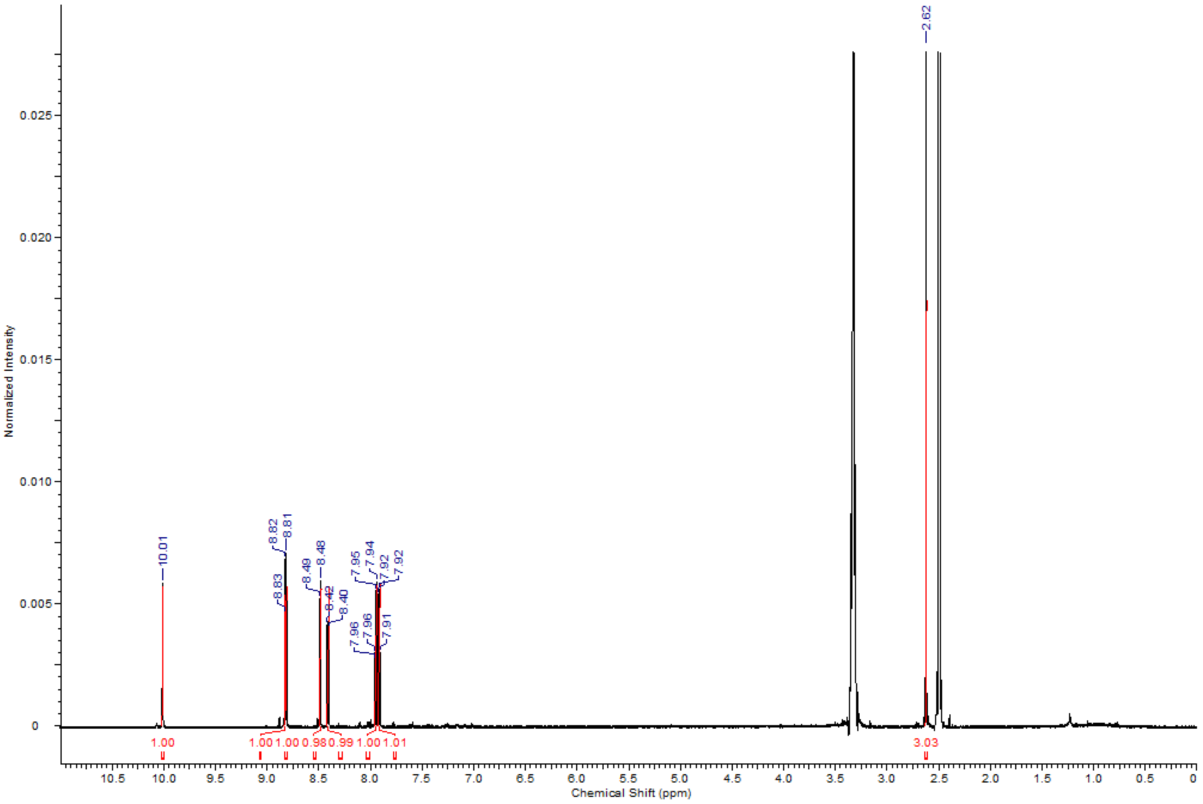

Figure S26. ^{1}H NMR spectrum of **10b** in DMSO-d_6.

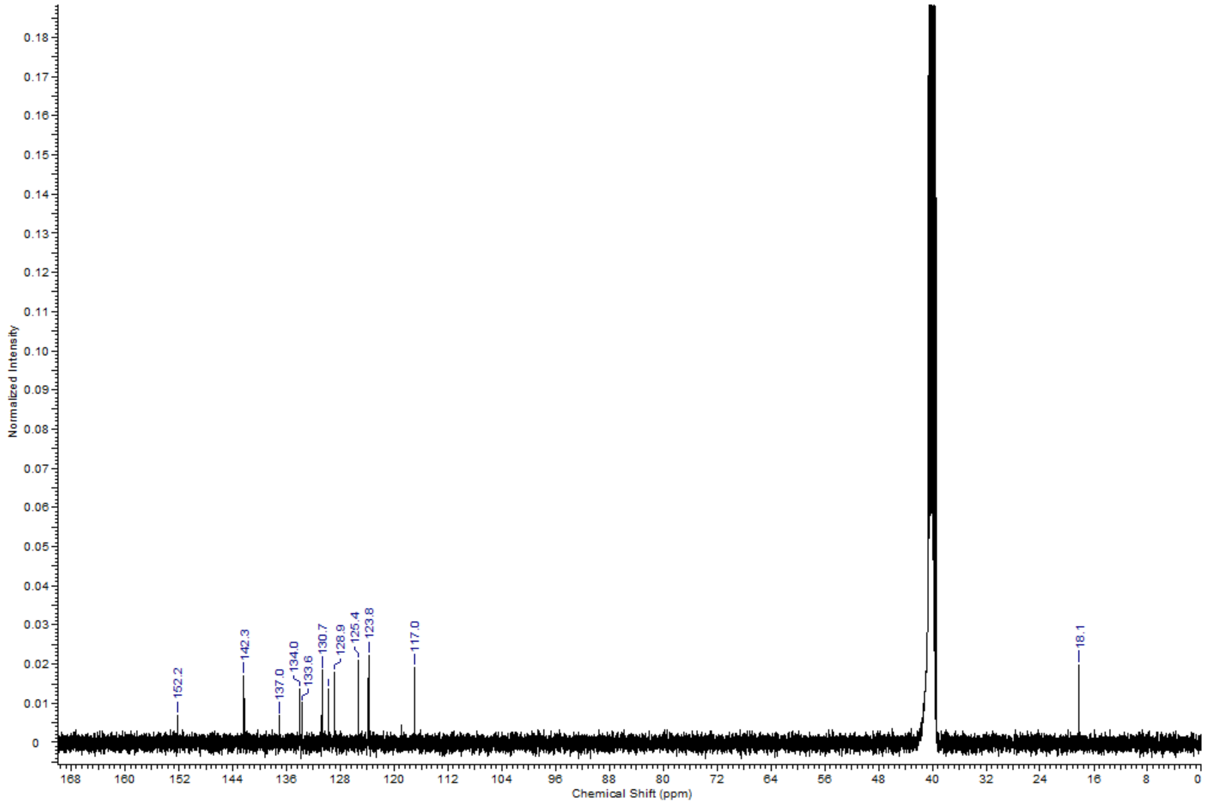

Figure S27. ^{13}C NMR spectrum of **10b** in DMSO-d_6.

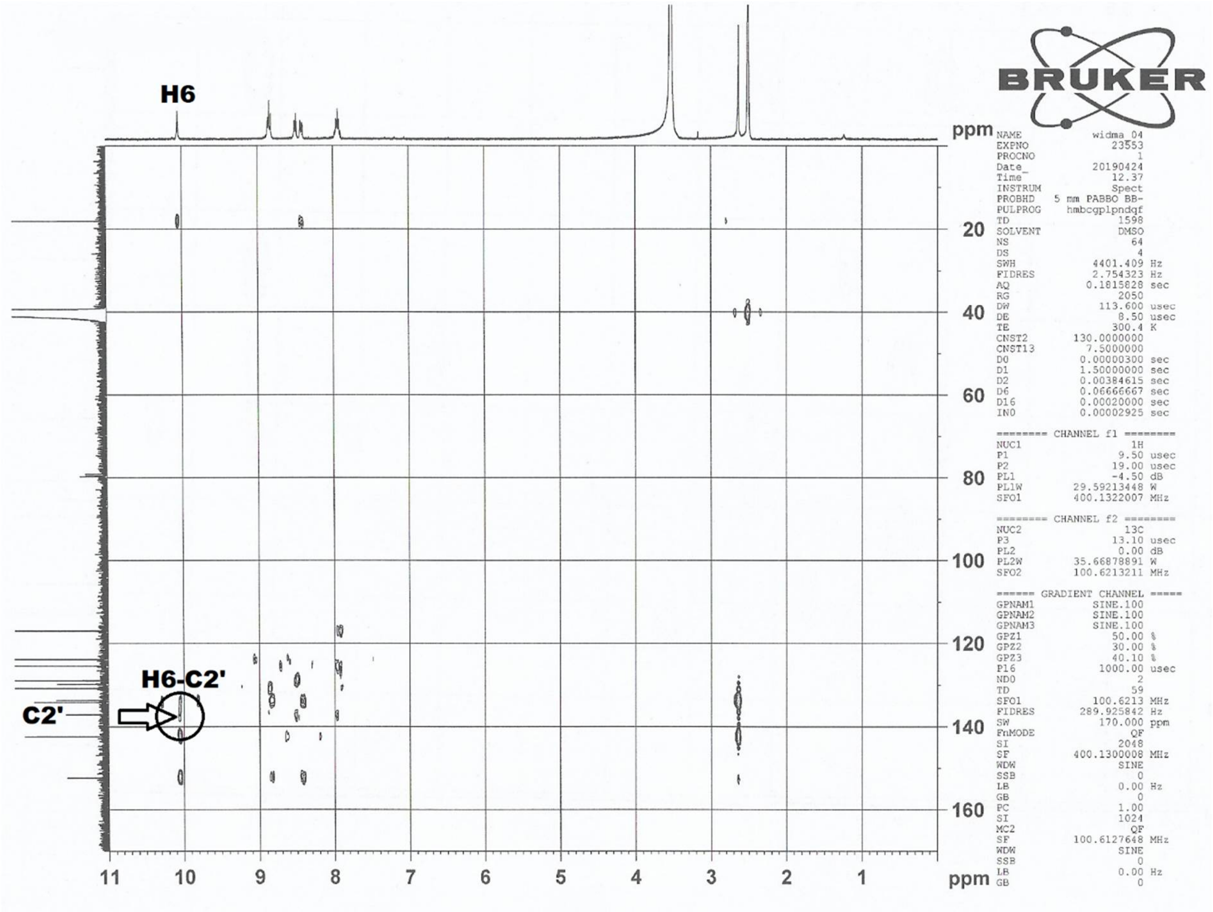

Figure S28. ^{1}H-^{13}C HMBC NMR spectrum of **10b** in DMSO-d$_6$.

Scheme S10. 4-Methylbenzo[4,5]thiazolo[3,2-*a*]pyridin-10-ium tetrachloroaurate(III) **11b.**

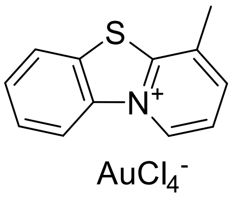

Yield: 82%; yellow powder; m.p. 154-156 °C; IR (ATR) $\tilde{\upsilon}$ 352 cm^{-1}; Anal. Calcd for $C_{12}H_{10}AuCl_4NS$: C, 26.74; H, 1.87; N, 2.60. Found C, 26.17; H, 2.02; N, 2.70.
^{1}H NMR (700 MHz, DMSO-*d*$_6$) δ 10.00 (d, *J* = 7.0 Hz, 1H, N-CH$_{ar}$), 8.88 (m, 1H, CH$_{ar}$), 8.54 (m, 1H, CH$_{ar}$), 8.42 (m, 1H, CH$_{ar}$), 8.14 (m, 1H, CH$_{ar}$), 7.98 (m, 1H, CH$_{ar}$), 7.95 (m, 1H, CH$_{ar}$), 2.74 (s, 3H, CH$_3$) ppm; ^{13}C NMR (101 MHz, DMSO-*d*$_6$) δ 154.1 (C$_{ar}$), 139.5 (CH$_{ar}$), 137.5 (C$_{ar}$), 133.6 (C$_{ar}$), 132.9 (CH$_{ar}$), 130.4 (CH$_{ar}$), 128.7 (CH$_{ar}$), 128.2 (C$_{ar}$), 124.9 (CH$_{ar}$), 122.4 (CH$_{ar}$), 117.0 (CH$_{ar}$), 18.6 (CH$_3$) ppm; ^{15}N NMR (71 MHz, DMSO-*d*$_6$) δ -168.8 ppm.

^{1}H and ^{13}C NMR assignments are in Tab. 1-2 in the main text.

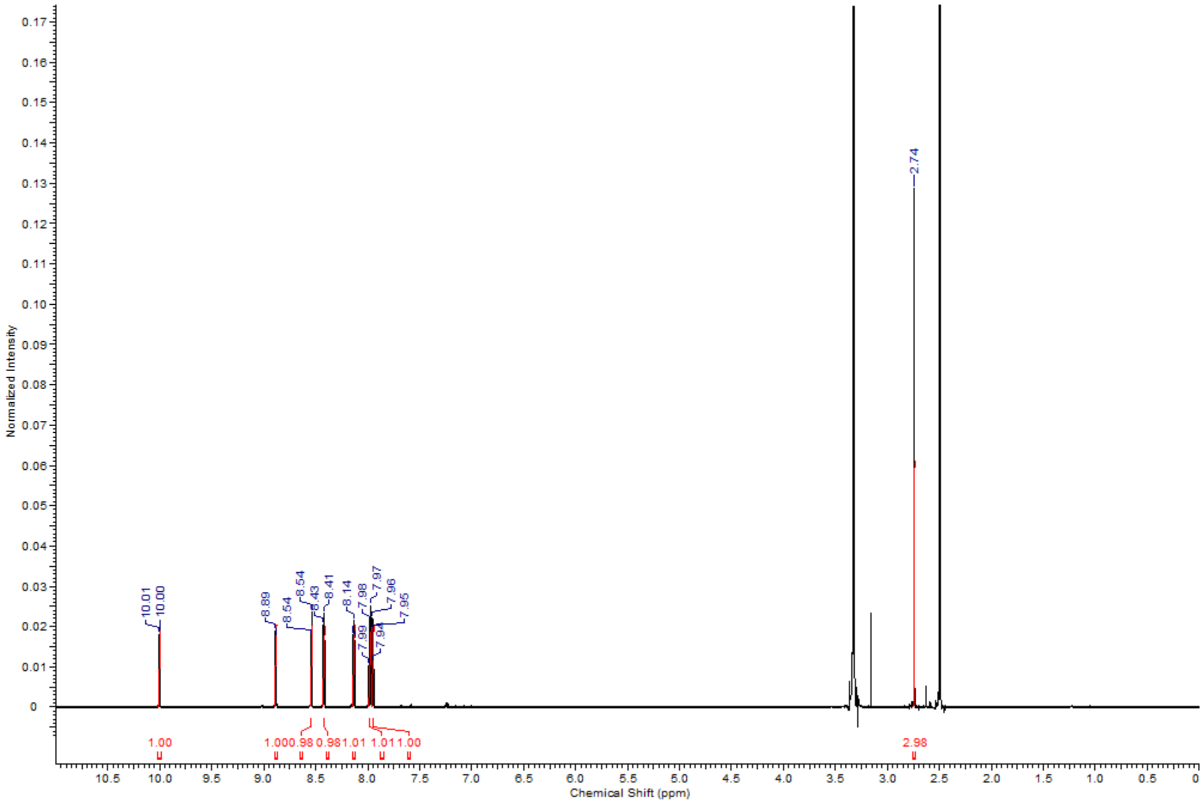

Figure S29. ^{1}H NMR spectrum of **11b** in DMSO-d_6.

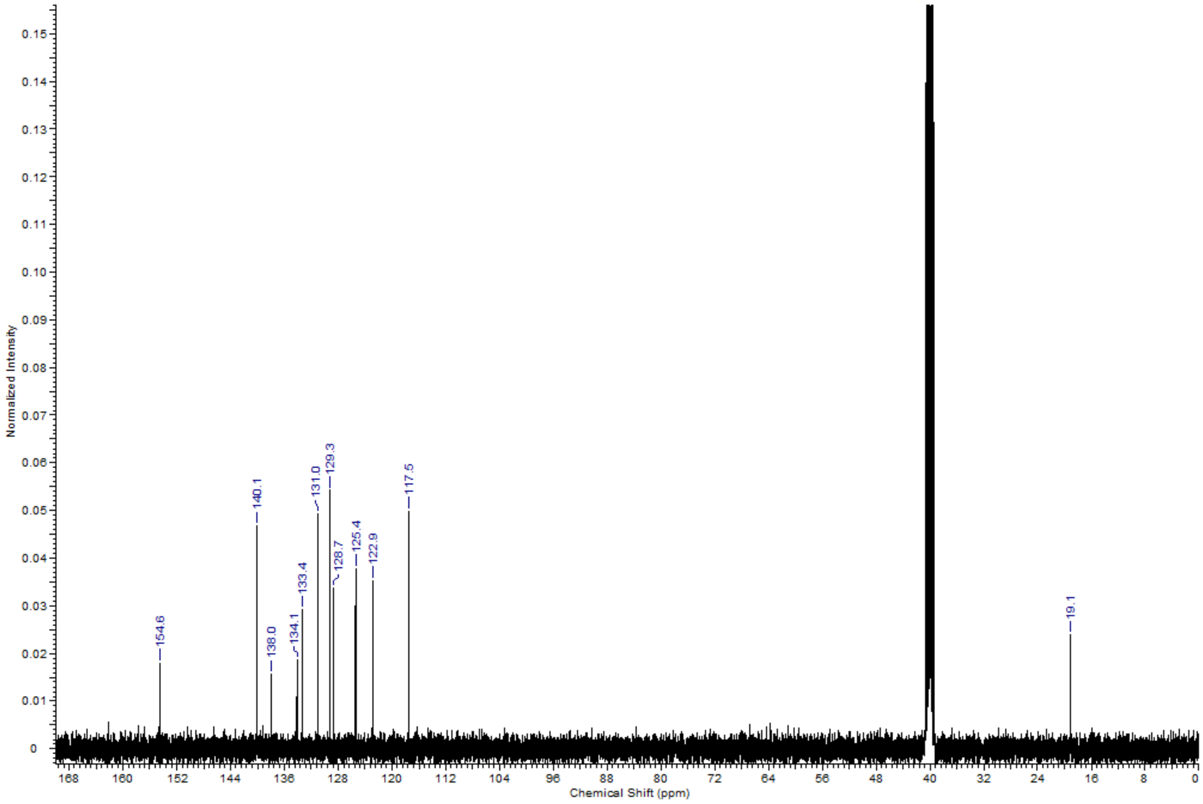

Figure S30. ^{13}C NMR spectrum of **11b** in DMSO-d_6.

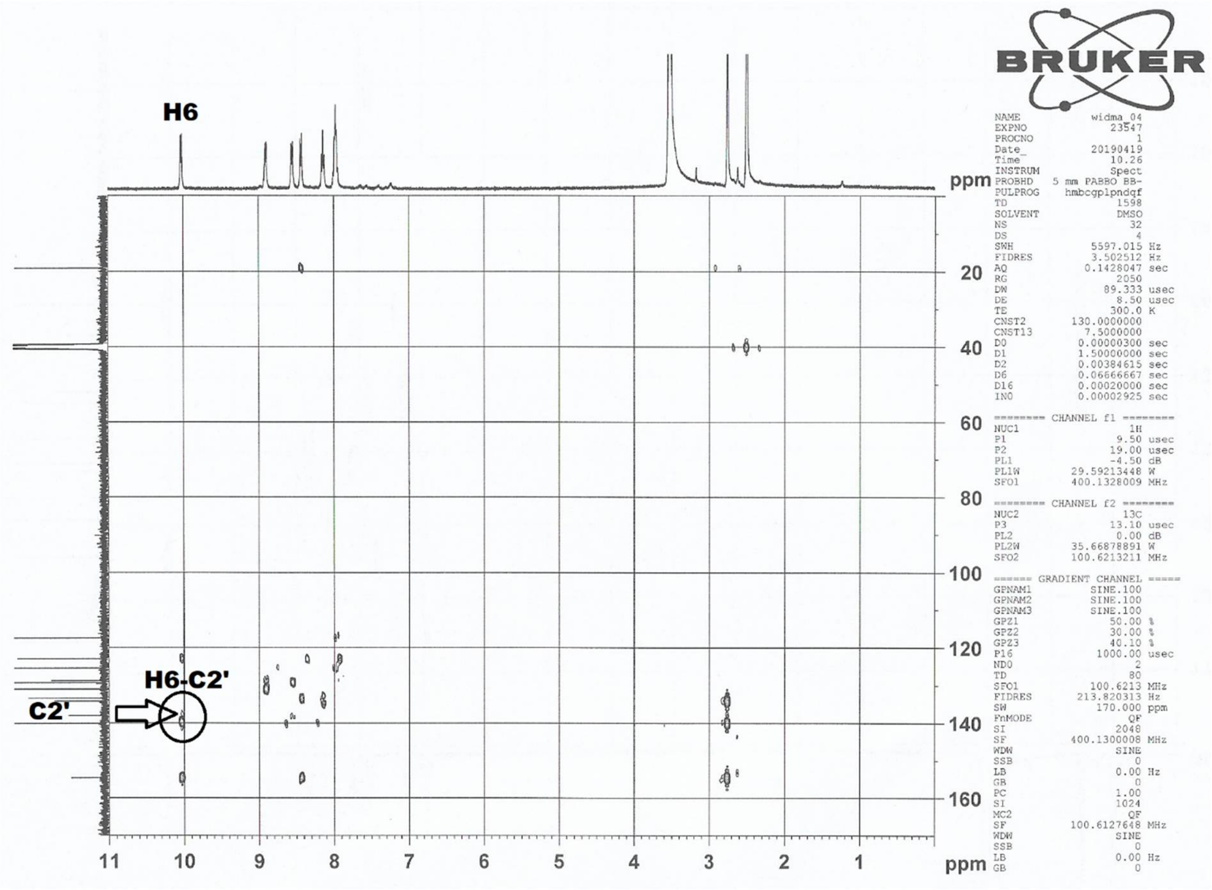

Figure S31. ^{1}H-^{13}C HMBC NMR spectrum of **11b** in DMSO-d$_6$.

Scheme S11. 8-Methylbenzo[4,5]thiazolo[3,2-*a*]pyridin-10-ium tetrachloroaurate(III) **12b.**

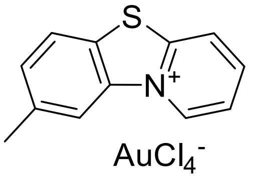

Yield: 66%; yellow powder; m.p. 204-206 °C; IR (ATR) $\tilde{\upsilon}$ 355 cm^{-1}; Anal. Calcd for $C_{12}H_{10}AuCl_4NS$: C, 26.74; H, 1.87; N, 2.60. Found C, 26.82; H, 1.84; N, 2.51.

^{1}H NMR (700 MHz, DMSO-*d*$_6$) δ 10.03 (d, *J* = 6.3 Hz, 1H, N-CH$_{ar}$), 8.91 (m, 1H, CH$_{ar}$), 8.75 (s, 1H, CH$_{ar}$), 8.49 (m, 1H, CH$_{ar}$), 8.37 (d, *J* = 8.4 Hz, 1H, CH$_{ar}$), 8.13 (m, 1H, CH$_{ar}$), 7.77 (m, 1H, CH$_{ar}$), 2.61 (s, 3H, CH$_3$) ppm; ^{13}C NMR (101 MHz, DMSO-*d*$_6$) δ 154.5 (C$_{ar}$), 139.7 (CH$_{ar}$), 139.0 (C$_{ar}$), 136.8 (C$_{ar}$), 134.7 (CH$_{ar}$), 131.7 (CH$_{ar}$), 126.1 (C$_{ar}$), 124.4 (CH$_{ar}$), 124.2 (CH$_{ar}$), 122.2 (CH$_{ar}$), 116.4 (CH$_{ar}$), 21.2 (CH$_3$) ppm; ^{15}N NMR (71 MHz, DMSO-*d*$_6$) δ -169.2 ppm.

^{1}H and ^{13}C NMR assignments are in Tab. 1-2 in the main text.

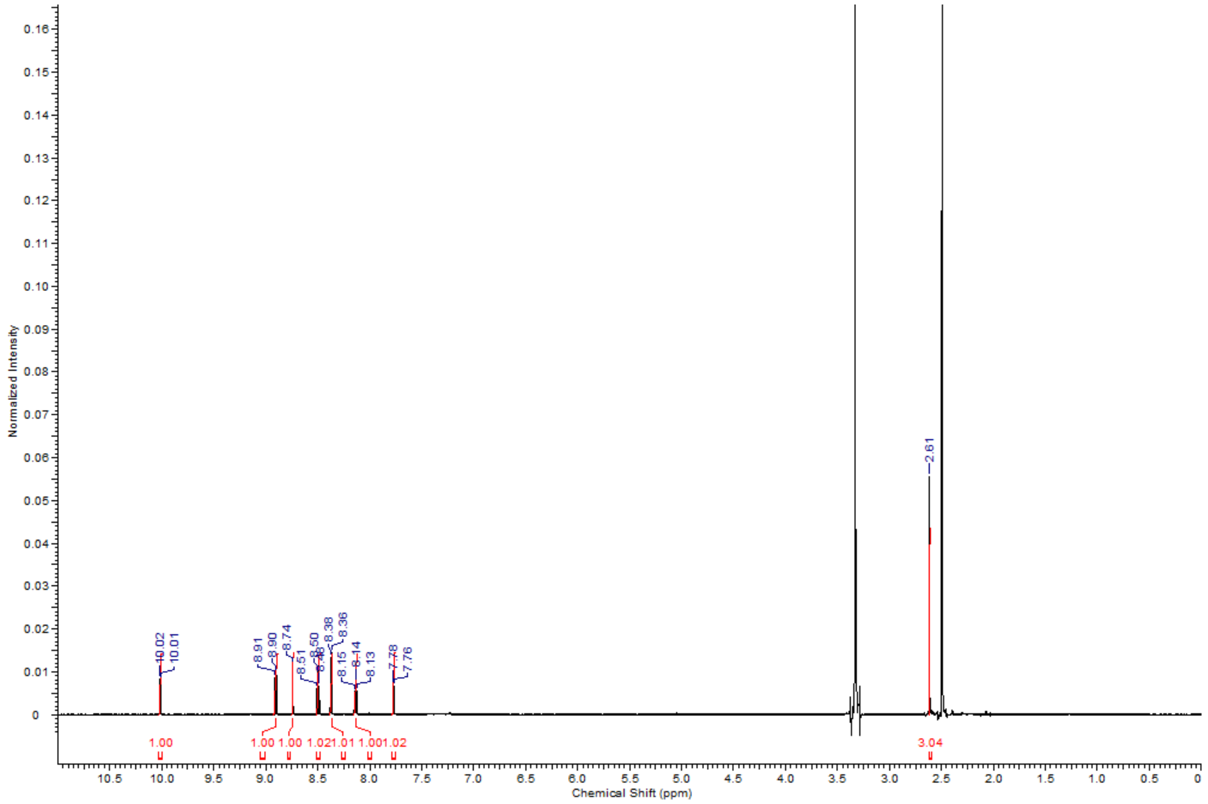

Figure S32. ^{1}H NMR spectrum of **12b** in DMSO-d_6.

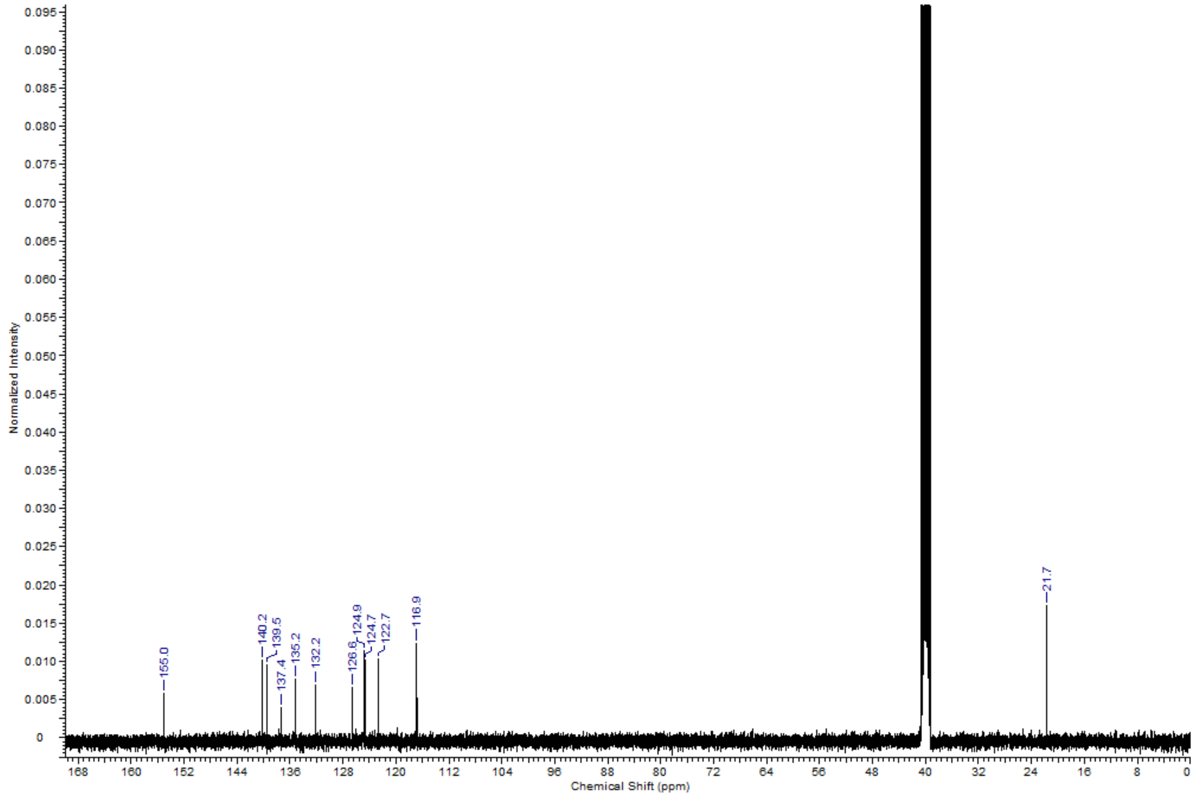

Figure S33. ^{13}C NMR spectrum of **12b** in DMSO-d_6.

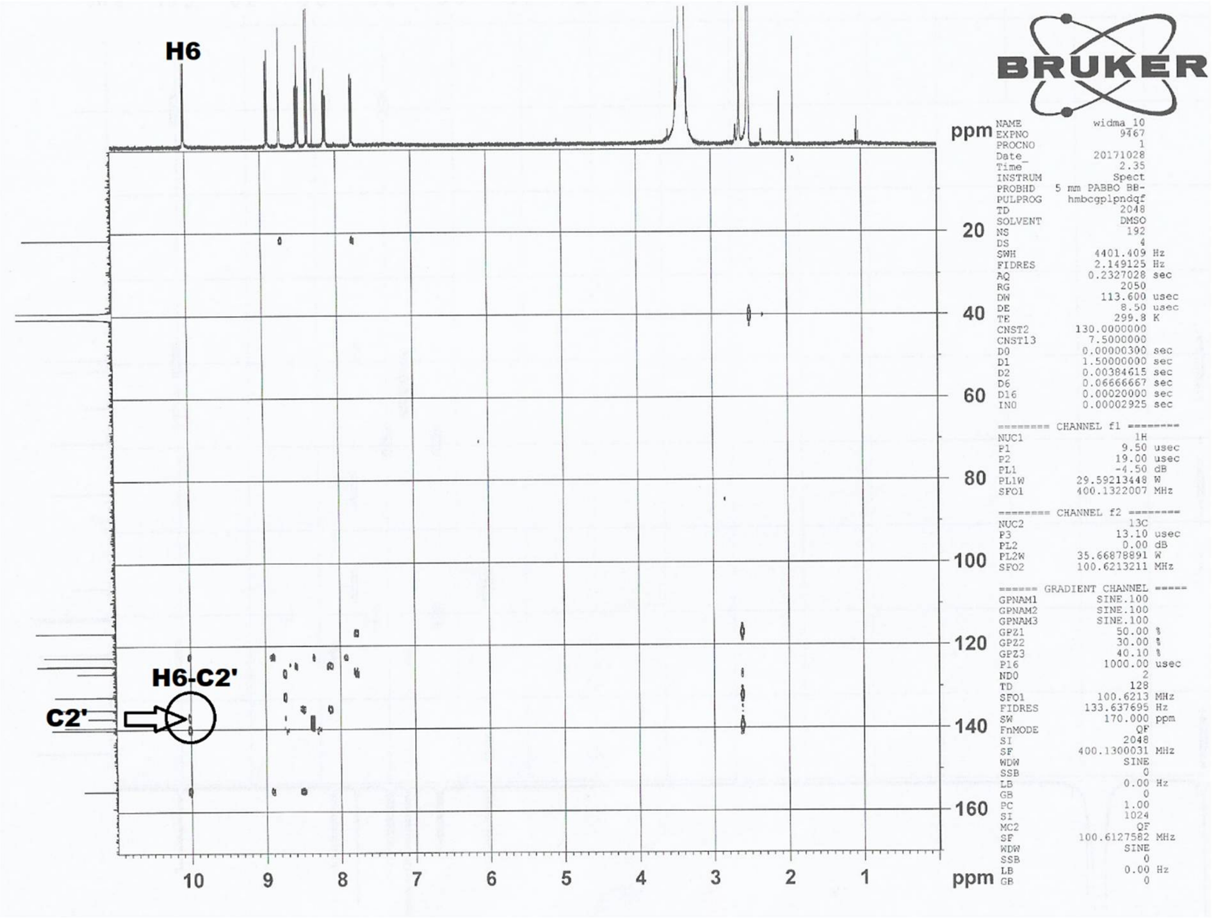

Figure S34. ^{1}H-^{13}C HMBC NMR spectrum of **12b** in DMSO-d_6.

Scheme S12. 8-(*tert*-Butyl)benzo[4,5]thiazolo[3,2-*a*]pyridin-10-ium tetrachloroaurate(III) **13b.**

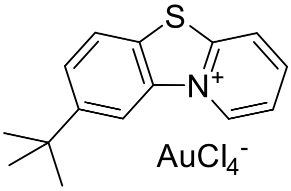

Yield: 75%; yellow powder; m.p. 178-180 °C; IR (ATR) $\tilde{\upsilon}$ 355 cm^{-1}; Anal. Calcd for $C_{15}H_{16}AuCl_4NS$: C, 31.00; H, 2.78; N, 2.41. Found C, 31.50; H, 2.99; N, 2.56.
^{1}H NMR (700 MHz, DMSO-d_6) δ 10.26 (d, *J* = 7.0 Hz, 1H, N-CH_{ar}), 8.91 (m, 1H, CH_{ar}), 8.84 (d, *J* = 2.1 Hz, 1H, CH_{ar}), 8.51 (m, 1H, CH_{ar}), 8.42 (d, *J* = 8.4 Hz, 1H, CH_{ar}), 8.15 (m, 1H, CH_{ar}), 8.03 (dd, *J* = 8.4, 1.4 Hz, 1H, CH_{ar}), 1.46 (s, 9H, $C(CH_3)_3$); ^{13}C NMR (101 MHz, DMSO-d_6) δ 154.6 (C_{ar}), 152.4 (C_{ar}), 139.7 (CH_{ar}), 136.9 (C_{ar}), 135.2 (CH_{ar}), 128.3 (CH_{ar}), 126.3 (C_{ar}), 124.3 (CH_{ar}), 124.2 (CH_{ar}), 122.0 (CH_{ar}), 113.2 (CH_{ar}), 35.5 ($\underline{C}(CH_3)_3$), 31.1 ($C(\underline{C}H_3)_3$) ppm; ^{15}N NMR (71 MHz, DMSO-d_6) δ -168.4 ppm.

^{1}H and ^{13}C NMR assignments are in Tab. 1-2 in the main text.

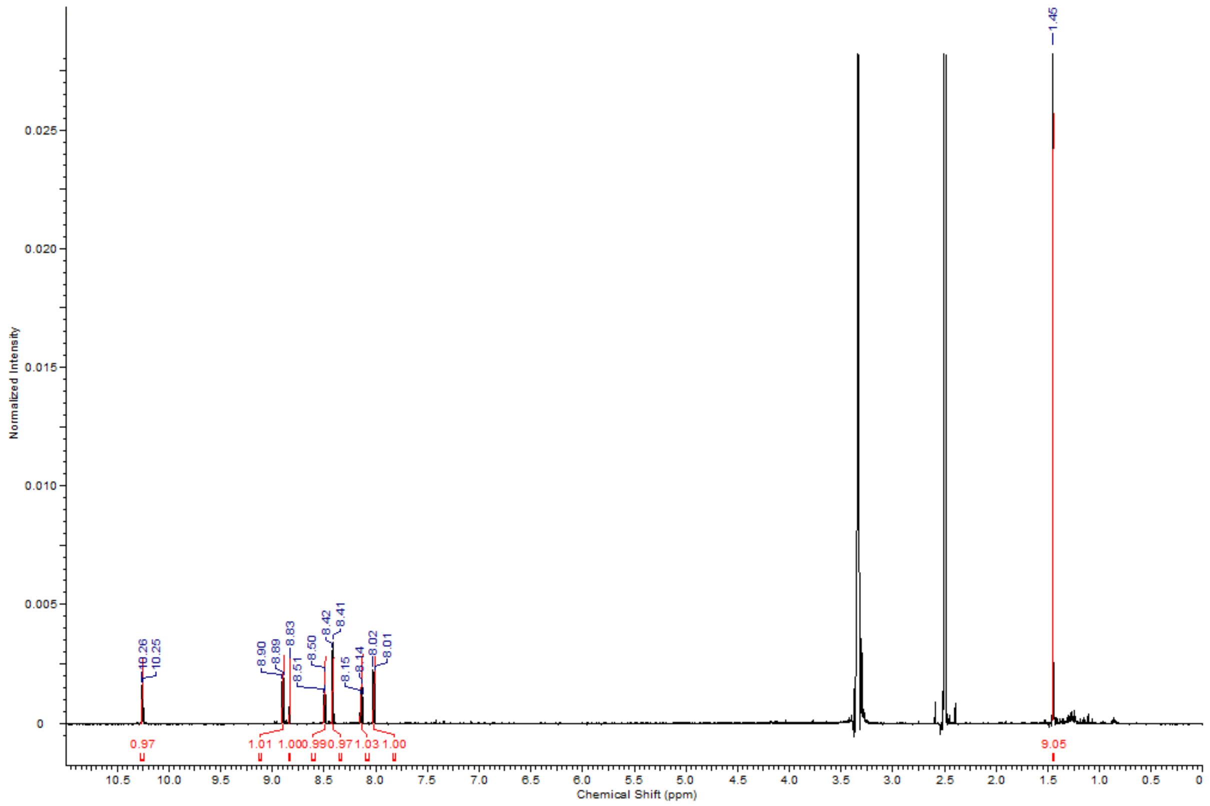

Figure S35. ^{1}H NMR spectrum of **13b** in DMSO-d6.

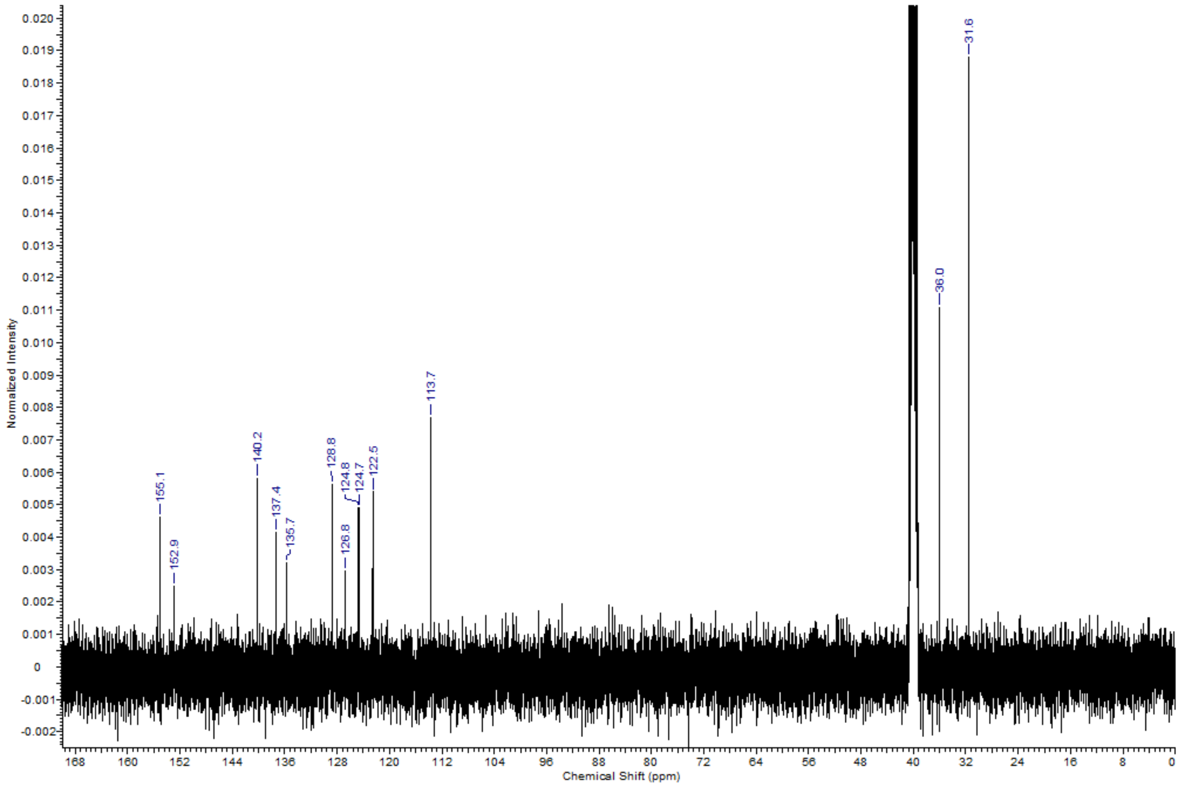

Figure S36. ^{13}C NMR spectrum of **13b** in DMSO-d6.

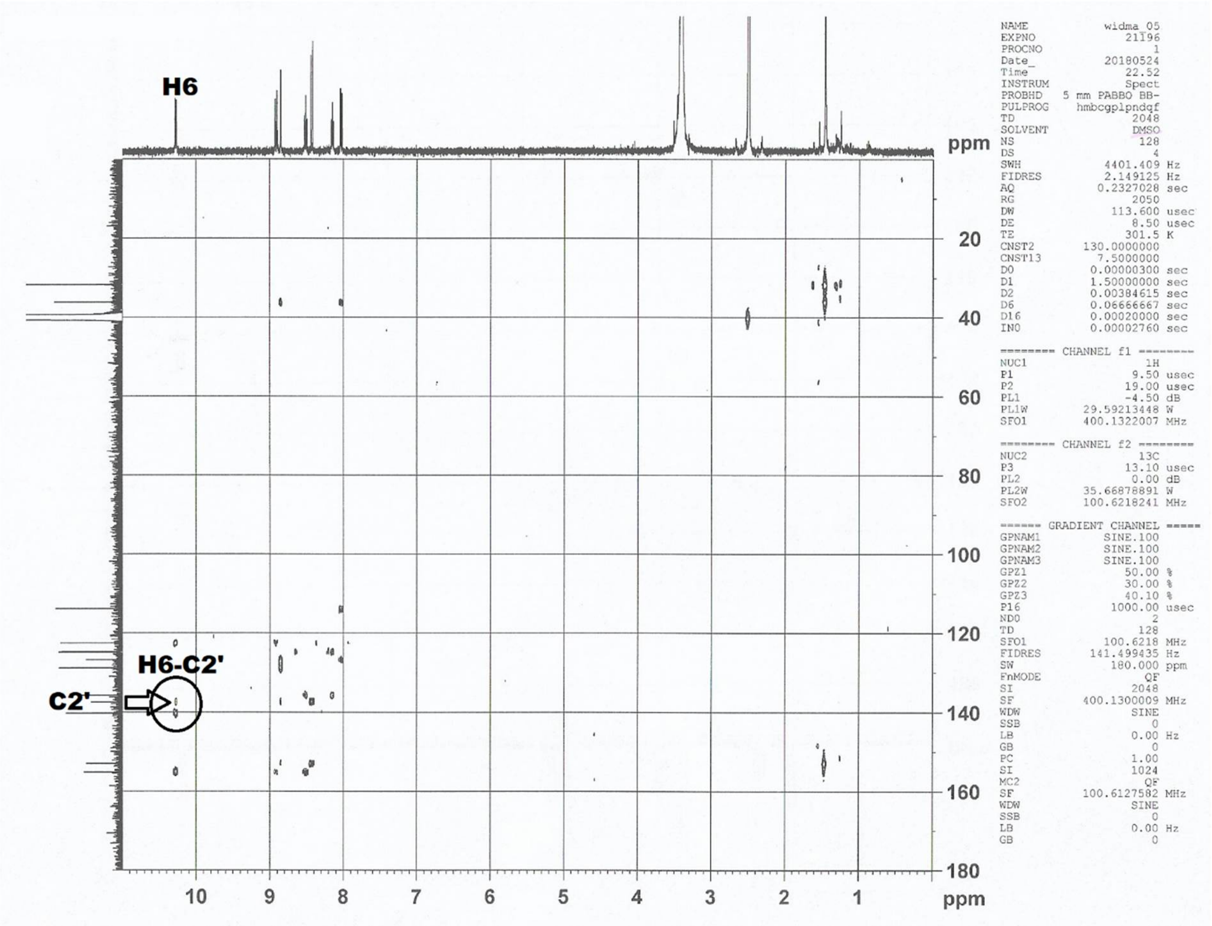

Figure S37. ^{1}H-^{13}C HMBC NMR spectrum of **13b** in DMSO-d$_6$.

Scheme S13. 8-Bromobenzo[4,5]thiazolo[3,2-a]pyridin-10-ium tetrachloroaurate(III) **14b.**

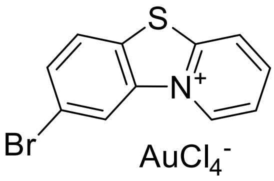

Yield: 53%; yellow powder; m.p. 227-229 °C; IR (ATR) $\bar{\upsilon}$ 350 cm^{-1}; Anal. Calcd for $C_{11}H_7AuBrCl_4N_2S$: C, 21.88; H, 1.17; N, 2.32. Found C, 22.02; H, 2.11; N, 2.52.

^{1}H NMR (700 MHz, DMSO-*d*$_6$) δ 10.08 (d, *J* = 7.0 Hz, 1H, N-CH$_{ar}$), 9.27 (d, *J* = 2.1 Hz, 1H, CH$_{ar}$), 8.93 (d, *J* = 9.1 Hz, 1H, CH$_{ar}$), 8.54 (m, 1H, CH$_{ar}$), 8.45 (d, *J* = 9.1 Hz, 1H, CH$_{ar}$), 8.16 (m, 1H, CH$_{ar}$), 8.12 (dd, *J* = 8.4, 2.1 Hz, 1H, CH$_{ar}$) ppm; ^{13}C NMR (101 MHz, DMSO-*d*$_6$) δ 155.0 (C$_{ar}$), 140.6 (CH$_{ar}$), 138.0 (C$_{ar}$), 135.4 (CH$_{ar}$), 133.2 (CH$_{ar}$), 128.6 (C$_{ar}$), 126.5 (CH$_{ar}$), 124.3 (CH$_{ar}$), 122.4 (CH$_{ar}$), 121.3 (C$_{ar}$), 119.9 (CH$_{ar}$) ppm; ^{15}N NMR (71 MHz, DMSO-*d*$_6$) δ -169.8 ppm.

^{1}H and ^{13}C NMR assignments are in Tab. 1-2 in the main text.

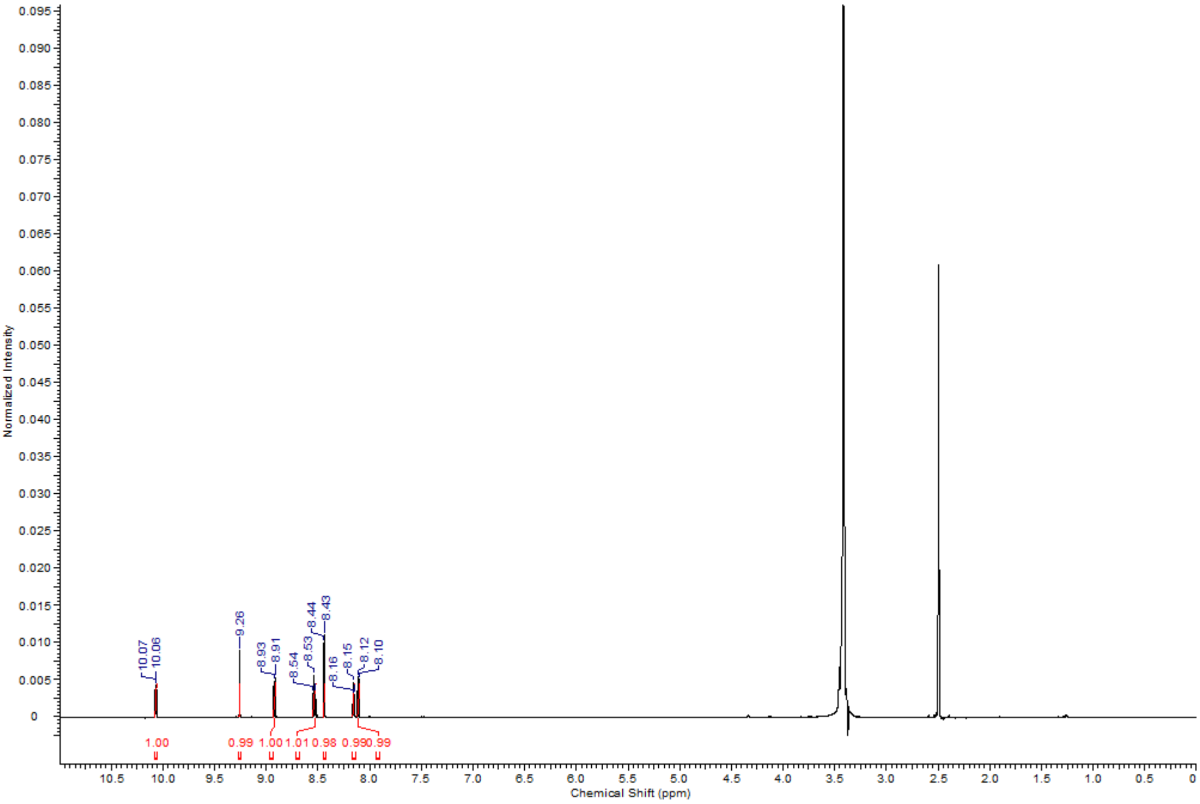

Figure S38. ^{1}H NMR spectrum of **14b** in DMSO-d_6.

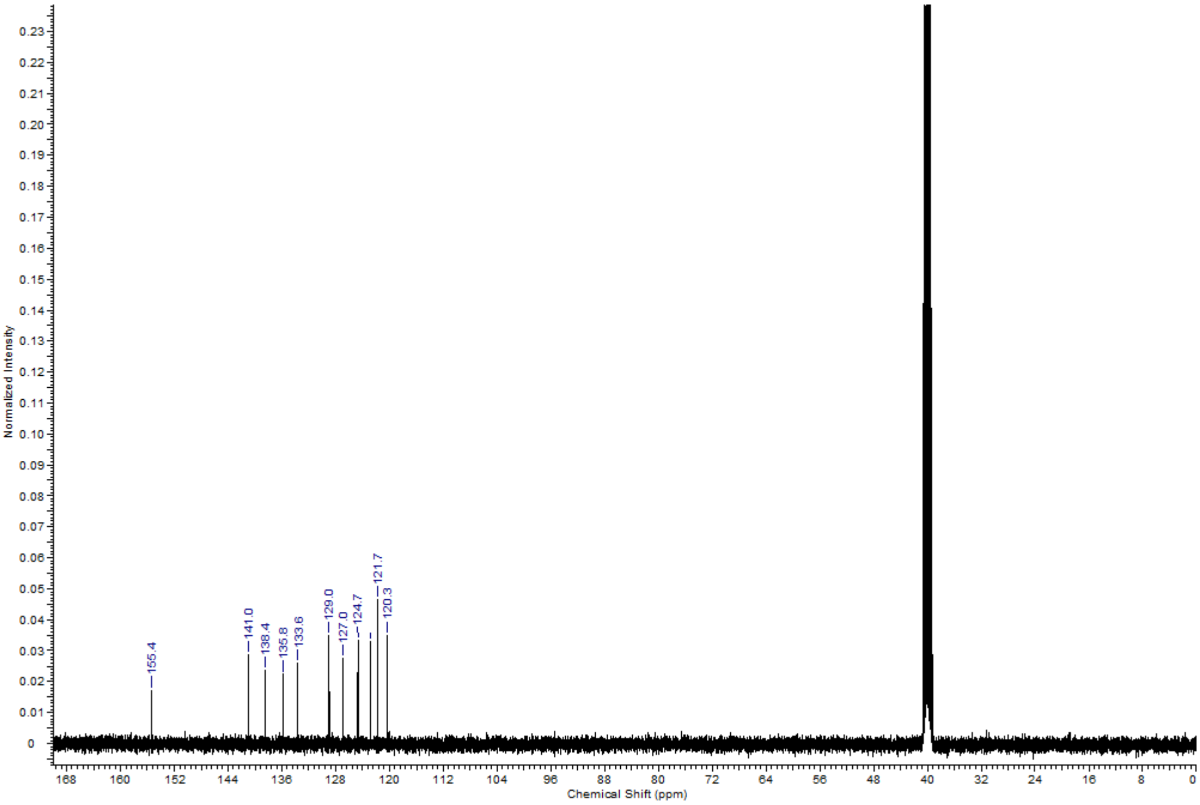

Figure S39. ^{13}C NMR spectrum of **14b** in DMSO-d_6.

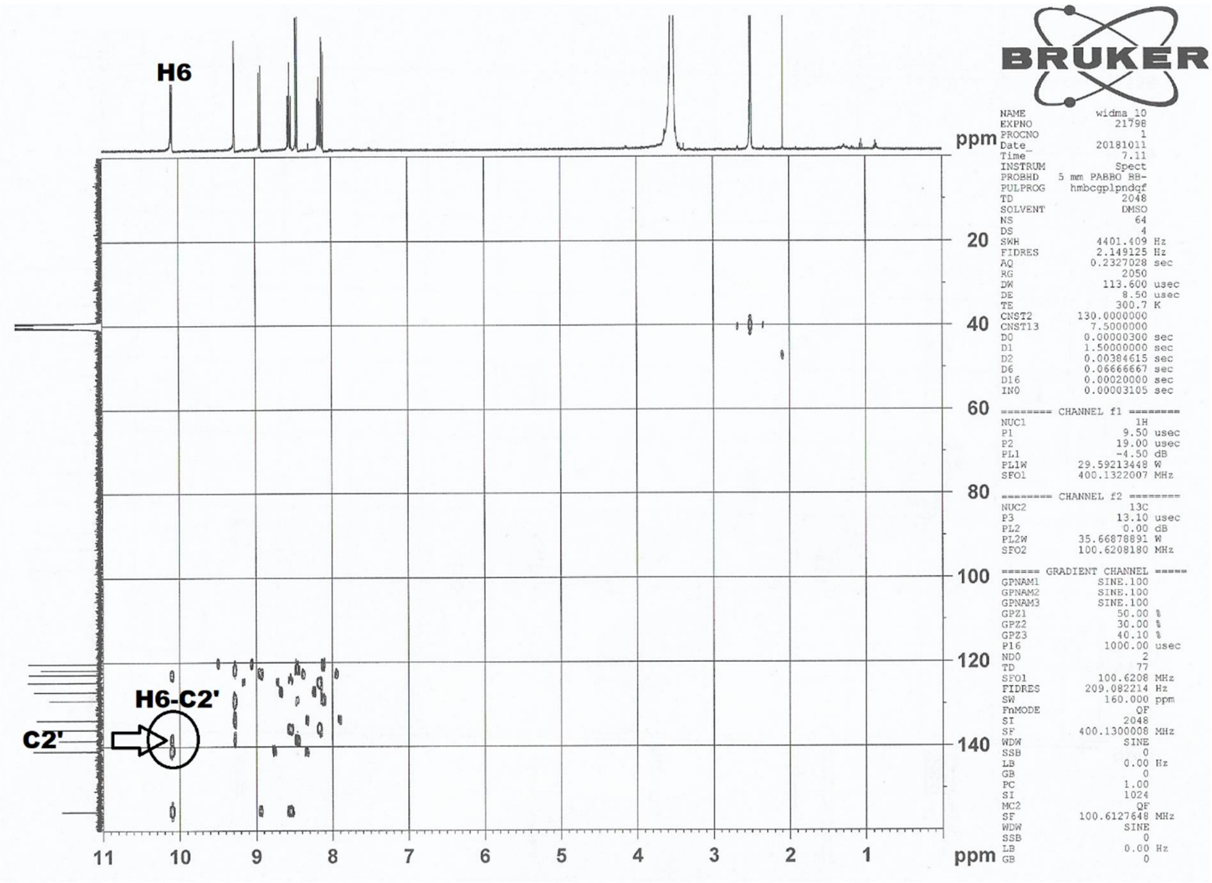

Figure S40. ^{1}H-^{13}C HMBC NMR spectrum of **14b** in DMSO-d_6.

Scheme S14. 8-Nitrobenzo[4,5]thiazolo[3,2-a]pyridin-10-ium tetrachloroaurate(III) **15b.**

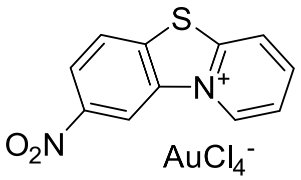

Yield: 65%; yellow powder; m.p. 228-230 °C; IR (ATR) $\tilde{\upsilon}$ 351 cm^{-1}; Anal. Calcd for $C_{11}H_7AuCl_4N_2O_2S$: C, 23.18; H, 1.24; N, 4.91. Found C, 23.15; H, 1.24; N, 4.91.

^{1}H NMR (700 MHz, DMSO-*d*$_6$) δ 10.40 (d, *J* = 7.0 Hz, 1H, N-CH_{ar}), 9.89 (d, *J* = 2.1 Hz, 1H, CH_{ar}), 9.00 (d, *J* = 8.4 Hz, 1H, CH_{ar}), 8.76 (m, 1H, CH_{ar}), 8.75 (m, 1H, CH_{ar}), 8.64 (m, 1H, CH_{ar}), 8.24 (m, 1H, CH_{ar}) ppm; ^{13}C NMR (101 MHz, DMSO-*d*$_6$) δ 155.8 (C_{ar}), 147.1 (C_{ar}), 141.5 (CH_{ar}), 137.3 (C_{ar}), 136.2 (CH_{ar}), 135.9 (C_{ar}), 126.2 (CH_{ar}), 124.5 (CH_{ar}), 124.4 (CH_{ar}), 122.7 (CH_{ar}), 113.1 (CH_{ar}) ppm; ^{15}N NMR (71 MHz, DMSO-*d*$_6$) δ -168.7, -11.1 ppm.

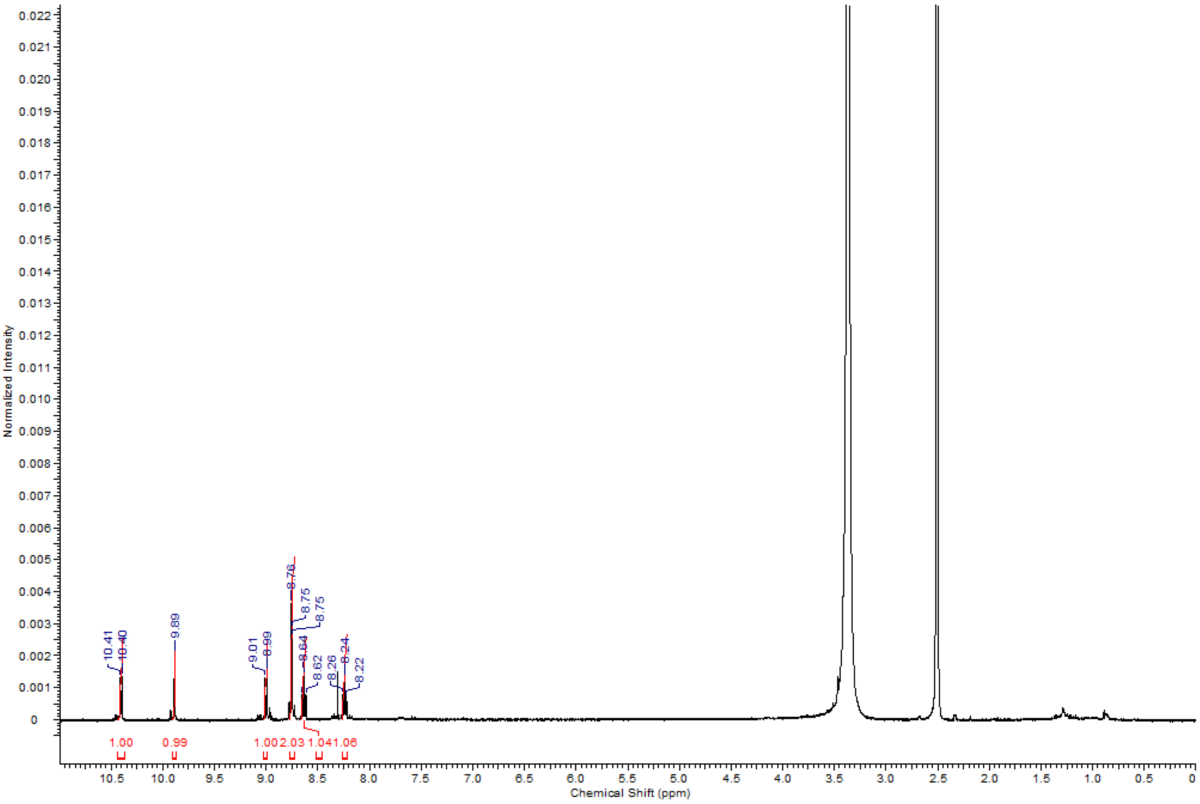

Figure S41. ^{1}H NMR spectrum of **15b** in DMSO-d_6.

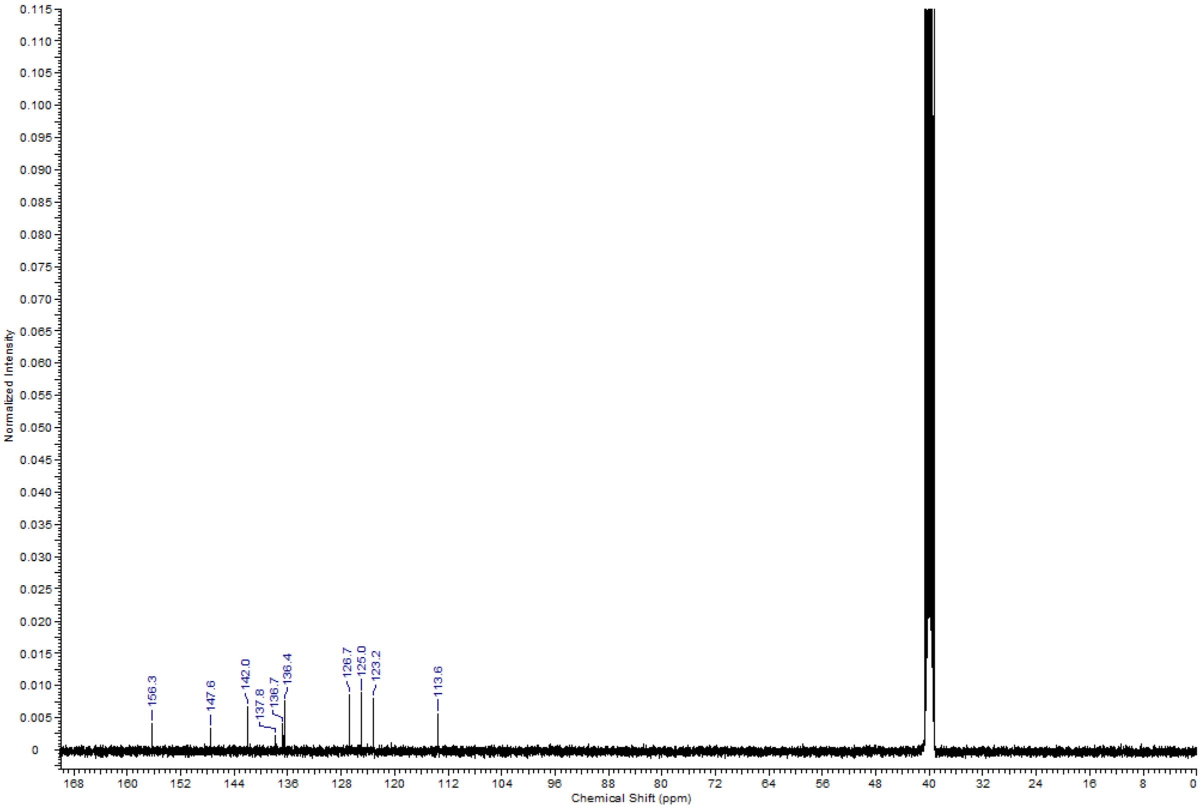

Figure S42. ^{13}C NMR spectrum of **15b** in DMSO-d_6.

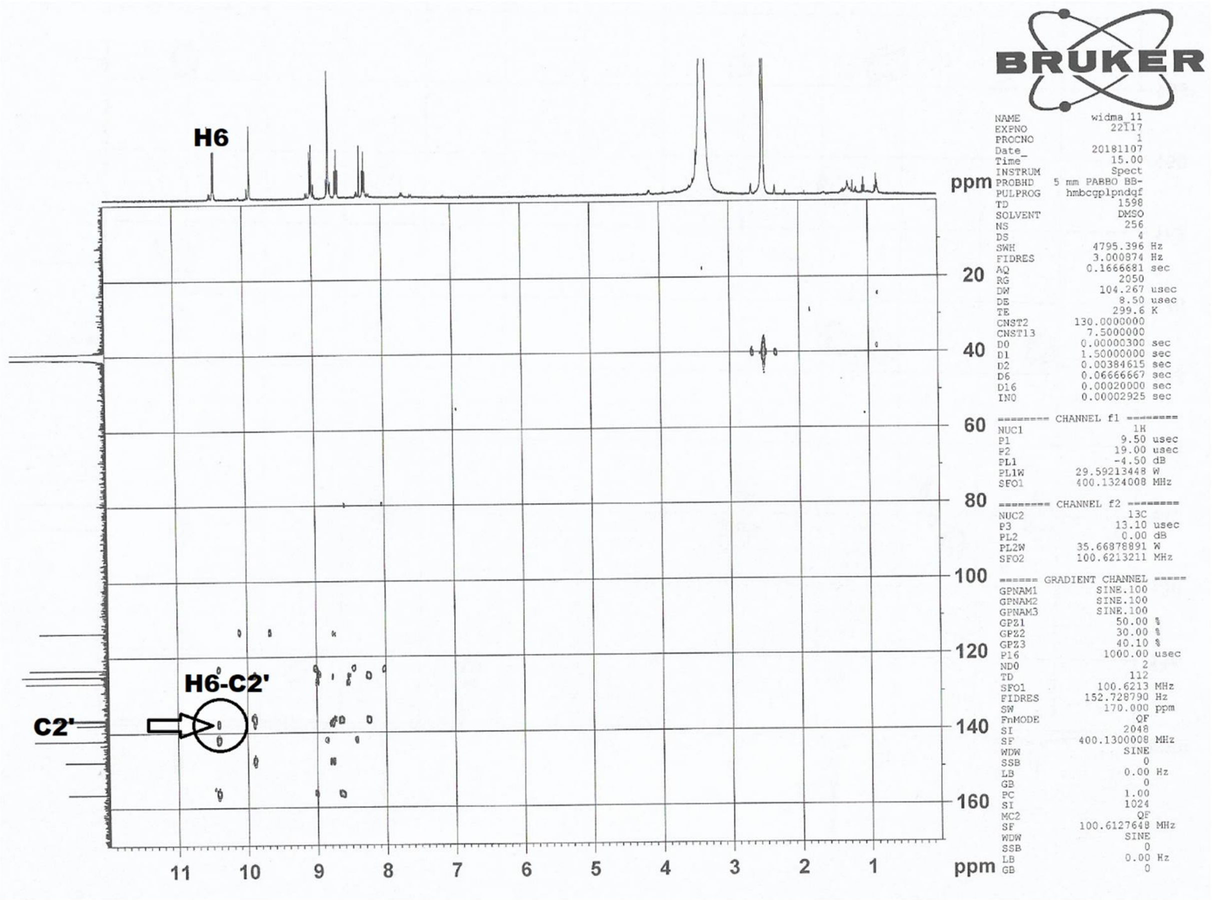

Figure S43. ^{1}H-^{13}C HMBC NMR spectrum of **15b** in DMSO-d_6.

S2. H, ^{13}C and ^{15}N chemical shifts of salts 8b-15b and parent heterocycles 8-15

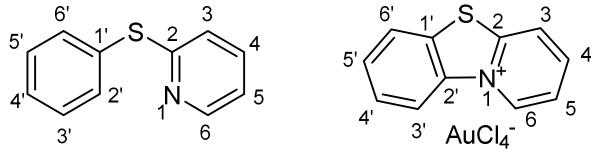

8 **8b**

Scheme S15. Numbering of heterocyclic **8** and cation **8b** rings.

Table S7. ^{1}H NMR chemical shifts for cations in the tetrachloroaurate(III) salts **8b**-**15b** (in DMSO-d$_6$; δ_{cat}^{1H}, ppm) and the relevant differences compared to the parent heterocycles **8**-**15** ($\Delta^{1H} = \delta_{cat}^{1H} - \delta_{het}^{1H}$, in parentheses).

Salt	H(3)	H(4)	H(5)	H(6)	H(3′)	H(4′)	H(5′)	H(6′)
**8b** [a]	8.94 (+2.01)	8.53 (+0.91)	8.16 (+1.03)	10.10 (+1.71)	8.88 (+1.41)	7.94 (+0.46)	7.96 (+0.49)	8.52 (+0.96)
**9b** [b]	8.82 (+2.17)	8.41 (+0.88)	7.99 (+0.98)	none[i]	8.90 (+1.41)	7.88 (+0.39)	7.93 (+0.44)	8.53 (+0.95)
**10b** [c]	8.83 (+1.93)	8.41 (+0.92)	none[j]	10.01 (+1.76)	8.83 (+1.38)	7.95 (+0.50)	7.91 (+0.46)	8.49 (+0.98)
**11b** [d]	none[k]	8.42 (+0.89)	8.14 (+1.07)	10.00 (+1.85)	8.88 (+1.51)	7.98 (+0.61)	7.95 (+0.58)	8.54 (+1.09)
**12b** [e]	8.91 (+2.05)	8.49 (+0.88)	8.13 (+1.02)	10.03 (+1.65)	8.75 (+1.45)	none[l]	7.77 (+0.47)	8.37 (+0.91)
**13b** [f]	8.91 (+1.99)	8.51 (+0.88)	8.15 (+1.02)	10.26 (+1.87)	8.84 (+1.33)	none[m]	8.03 (+0.52)	8.42 (+0.91)
**14b** [g]	8.93 (+1.87)	8.54 (+0.87)	8.16 (+0.99)	10.08 (+1.68)	9.27 (+1.61)	none	8.12 (+0.46)	8.45 (+0.95)
**15b** [h]	9.00 (+1.58)	8.64 (+0.84)	8.24 (+0.91)	10.40 (+1.89)	9.89 (+1.67)	none	8.75 (+0.53)	8.76 (+1.07)

[a] *vs* **8** in DMSO-d$_6$: H(3) 6.93, H(4) 7.62, H(5) 7.13, H(6) 8.39, H(2′/6′) 7.56, H(3′/5′) 7.47, H(4′) 7.48 ppm [17]

[b] *vs* **9** in DMSO-d$_6$: H(3) 6.65, H(4) 7.53, H(5) 7.01, H(2′/6′) 7.58, H(3′/5′) 7.49, H(4′) 7.49, CH_3 2.40 ppm

[c] *vs* **10** in DMSO-d$_6$: H(3) 6.90, H(4) 7.49, H(6) 8.25, H(2′/6′) 7.51, H(3′/5′) 7.45, H(4′) 7.45, CH_3 2.22 ppm

[d] *vs* **11** in DMSO-d$_6$: H(4) 7.53, H(5) 7.07, H(6) 8.15, H(2′/6′) 7.45, H(3′/5′) 7.37, H(4′) 7.37, CH_3 2.28 ppm

[e] *vs* **12** in DMSO-d$_6$: H(3) 6.86, H(4) 7.61, H(5) 7.11, H(6) 8.38, H(2′/6′) 7.46, H(3′/5′) 7.30, CH_3 2.35 ppm [17]

[f] *vs* **13** in DMSO-d$_6$: H(3) 6.92, H(4) 7.63, H(5) 7.13, H(6) 8.39, H(2′/6′) 7.51, H(3′/5′) 7.51, $C(CH_3)_3$ 1.31 ppm [17]

[g] *vs* **14** in DMSO-d$_6$: H(3) 7.06, H(4) 7.67, H(5) 7.17, H(6) 8.40, H(2′/6′) 7.50, H(3′/5′) 7.66 ppm [17]

[h] *vs* **15** in DMSO-d$_6$: H(3) 7.42, H(4) 7.80, H(5) 7.33, H(6) 8.51, H(2′/6′) 7.69, H(3′/5′) 8.22 ppm [17]

[i] CH_3 3.40 ppm (+1.00 ppm)

[j] CH_3 2.62 ppm (+0.40 ppm)

[k] CH_3 2.74 ppm (+0.46 ppm)

[l] CH_3 2.61 ppm (+0.26 ppm)

[m] $C(CH_3)_3$ 1.46 ppm (+0.15 ppm)

Table S8. ^{13}C NMR chemical shifts for cations in the tetrachloroaurate(III) salts **8b**-**15b** (in DMSO-d$_6$; δ_{cat}^{13C}, ppm) and the relevant differences compared to the parent heterocycles **8**-**15** ($\Delta^{13C} = \delta_{cat}^{13C} - \delta_{het}^{13C}$, in parentheses).

Salt	C(2)	C(3)	C(4)	C(5)	C(6)	C(1′)	C(2′)	C(3′)	C(4′)	C(5′)	C(6′)

**8b** [a]	154.3 (−5.6)	124.2 (+3.1)	140.0 (+2.7)	122.3 (+1.9)	135.1 (−14.4)	129.1 (−1.1)	136.7 (+2.1)	116.7 (−13.1)	128.5 (−0.7)	130.3 (+0.5)	124.9 (−9.7)
**9b** [b]	155.2 (−4.0)	121.9 (+3.8)	139.2 (+1.6)	125.4 (+5.6)	150.9[i] (−7.3)	129.3 (−1.3)	138.5 (+4.2)	121.6 (−8.2)	128.2 (−0.9)	129.7 (−0.1)	124.6 (−9.7)
**10b** [c]	151.7 (−4.3)	123.3 (+1.7)	141.8 (+3.6)	133.1[j] (+2.8)	133.5 (−16.2)	129.3 (−1.9)	136.5 (+2.5)	116.5 (−13.3)	128.4 (−0.6)	130.2 (+0.4)	124.9 (−9.1)
**11b** [d]	154.1 (−2.4)	133.6[k] (+2.5)	139.5 (+2.0)	122.4 (+1.5)	132.9 (−14.0)	128.2 (−2.3)	137.5 (+3.3)	117.0 (−12.1)	128.7 (+0.4)	130.4 (+1.3)	124.9 (−9.3)
**12b** [e]	154.5 (−6.1)	124.2 (+3.7)	139.7 (+2.4)	122.2 (+2.0)	134.7 (−14.7)	126.1 (−0.3)	136.8 (+1.8)	116.4 (−14.1)	139.0[l] (−0.3)	131.7 (+1.2)	124.4 (−10.6)
**13b** [f]	154.6 (−5.7)	124.2 (+3.5)	139.7 (+2.4)	122.0 (+1.7)	135.2 (−14.3)	126.3 (−0.4)	136.9 (+2.4)	113.2 (−13.5)	152.4[m] (+0.4)	128.3 (+1.6)	124.3 (−10.2)
**14b** [g]	155.0 (−3.7)	124.3 (+2.7)	140.6 (+3.1)	122.4 (+1.6)	135.4 (−14.3)	128.6 (−1.3)	138.0 (+1.7)	119.9 (−12.7)	121.3 (−1.4)	133.2 (+0.6)	126.5 (−9.8)
**15b** [h]	155.8 (+0.2)	124.4 (−0.3)	141.5 (+3.5)	122.7 (+0.3)	136.2 (-14.1)	135.9 (−5.7)	137.3 (+5.2)	113.1 (−11.1)	147.1 (+0.5)	124.5 (+0.3)	126.2 (−5.9)

[a] *vs* **8** in DMSO-d_6: C(2) 159.9, C(3) 121.1, C(4) 137.3, C(5) 120.4, C(6) 149.5, C(1′) 130.2, C(2′/6′) 134.6, C(3′/5′) 129.8, C(4′) 129.2 ppm [17]

[b] *vs* **9** in DMSO-d_6: C(2) 159.2 C(3) 118.1, C(4) 137.6, C(5) 119.8, C(6) 158.2, C(1′) 130.6, C(2′/6′) 134.3, C(3′/5′) 129.8, C(4′) 129.1 ppm, CH_3 23.7 ppm

[c] *vs* **10** in DMSO-d_6: C(2) 156.0 C(3) 121.6, C(4) 138.2, C(5) 130.3, C(6) 149.7, C(1′) 131.2, C(2′/6′) 134.0, C(3′/5′) 129.8, C(4′) 129.0 ppm, CH_3 17.3 ppm

[d] *vs* **11** in DMSO-d_6: C(2) 156.5 C(3) 131.1, C(4) 137.5, C(5) 120.9, C(6) 146.9, C(1′) 130.5, C(2′/6′) 134.2, C(3′/5′) 129.1, C(4′) 128.3 ppm, CH_3 18.3 ppm

[e] *vs* **12** in DMSO-d_6: C(2) 160.6, C(3) 120.5, C(4) 137.3, C(5) 120.2, C(6) 149.4, C(1′) 126.4, C(2′/6′) 135.0, C(3′/5′) 130.5, C(4′) 139.3, CH_3 20.8 ppm [17]

[f] *vs* **13** in DMSO-d_6: C(2) 160.3, C(3) 120.7, C(4) 137.3, C(5) 120.3, C(6) 149.5, C(1′) 126.7, C(2′/6′) 134.5, C(3′/5′) 126.7, C(4′) 152.0, **C**(CH_3) 34.5 ppm, C($\underline{C}H_3$) 30.9 ppm [17]

[g] *vs* **14** in DMSO-d_6: C(2) 158.7, C(3) 121.6, C(4) 137.5, C(5) 120.8, C(6) 149.7, C(1′) 129.9, C(2′/6′) 136.3, C(3′/5′) 132.6, C(4′) 122.7 ppm [17]

[h] *vs* **15** in DMSO-d_6: C(2) 155.6, C(3) 124.7, C(4) 138.0, C(5) 122.4, C(6) 150.3, C(1′) 141.6, C(2′/6′) 132.1, C(3′/5′) 124.2, C(4′) 146.6 ppm [17]

[i] CH_3 24.0 ppm (+0.3 ppm)

[j] CH_3 17.6 ppm (+0.3 ppm)

[k] CH_3 18.6 ppm (+0.3 ppm)

[l] CH_3 21.2 ppm (+0.4 ppm)

[m] **C**(CH_3) = 35.5 ppm (+1.0 ppm), C($\underline{C}H_3$) 31.1 ppm (0.2 ppm)

Table S9. ^{15}N NMR chemical shifts for cations in the tetrachloroaurate(III) salts **8b**-**15b** (in DMSO-d_6; $\delta_{cat}{}^{15N}$, ppm) and the relevant differences compared to the parent heterocycles **8**-**15** ($\Delta^{15N} = \delta_{cat}{}^{15N} - \delta_{het}{}^{15N}$, in parentheses).

Salt	N(1)
**8b** [a]	−168.9 (−96.9)
**9b** [b]	−167.6 (−89.6)
**10b** [c]	−168.1 (−97.5)
**11b** [d]	−168.8 (−96.4)
**12b** [e]	−169.2 (−96.5)
**13b** [f]	−168.4 (−96.0)
**14b** [g]	−169.8 (−98.1)
**15b** [h]	−168.7[i] (−101.9)

[a] *vs* **8** in DMSO-d_6: N(1) -72.0 ppm [17]

[b] *vs* **9** in DMSO-d_6: N(1) -78.0 ppm

[c] *vs* **10** in DMSO-d_6: N(1) -70.6 ppm

[d] *vs* **11** in DMSO-d_6: N(1) -72.4 ppm
[e] *vs* **12** in DMSO-d_6: N(1) -72.7 ppm [17]
[f] *vs* **13** in DMSO-d_6: N(1) -72.4 ppm [17]
[g] *vs* **14** in DMSO-d_6: N(1) -71.7 ppm [17]
[h] *vs* **15** in DMSO-d_6: N(1) -66.8 ppm, NO_2 -9.0 ppm [17]
[i] NO_2 -11.1 ppm (-2.1 ppm)
